# Discovery and validation of genes driving drug‐intake and related behavioral traits in mice

**DOI:** 10.1111/gbb.12875

**Published:** 2024-01-02

**Authors:** Tyler A. Roy, Jason A. Bubier, Price E. Dickson, Troy D. Wilcox, Juliet Ndukum, James W. Clark, Stacey J. Sukoff Rizzo, John C. Crabbe, James M. Denegre, Karen L. Svenson, Robert E. Braun, Vivek Kumar, Stephen A. Murray, Jacqueline K. White, Vivek M. Philip, Elissa J. Chesler

**Affiliations:** ^1^ Center for Addiction Biology The Jackson Laboratory Bar Harbor Maine USA; ^2^ Joan C Edwards School of Medicine Marshall University Huntington West Virginia USA; ^3^ School of Medicine University of Pittsburgh Pittsburgh Pennsylvania USA; ^4^ VA Portland Health Care System Oregon Health & Science University Portland Oregon USA

**Keywords:** addiction, addiction predicative, alcohol, genetic screen, knock‐out, KOMP, methamphetamine, mouse mutants, neurogenetics, nicotine, phenotyping

## Abstract

Substance use disorders are heritable disorders characterized by compulsive drug use, the biological mechanisms for which remain largely unknown. Genetic correlations reveal that predisposing drug‐naïve phenotypes, including anxiety, depression, novelty preference and sensation seeking, are predictive of drug‐use phenotypes, thereby implicating shared genetic mechanisms. High‐throughput behavioral screening in knockout (KO) mice allows efficient discovery of the function of genes. We used this strategy in two rounds of candidate prioritization in which we identified 33 drug‐use candidate genes based upon predisposing drug‐naïve phenotypes and ultimately validated the perturbation of 22 genes as causal drivers of substance intake. We selected 19/221 KO strains (8.5%) that had a difference from control on at least one drug‐naïve predictive behavioral phenotype and determined that 15/19 (~80%) affected the consumption or preference for alcohol, methamphetamine or both. No mutant exhibited a difference in nicotine consumption or preference which was possibly confounded with saccharin. In the second round of prioritization, we employed a multivariate approach to identify outliers and performed validation using methamphetamine two‐bottle choice and ethanol drinking‐in‐the‐dark protocols. We identified 15/401 KO strains (3.7%, which included one gene from the first cohort) that differed most from controls for the predisposing phenotypes. 8 of 15 gene deletions (53%) affected intake or preference for alcohol, methamphetamine or both. Using multivariate and bioinformatic analyses, we observed multiple relations between predisposing behaviors and drug intake, revealing many distinct biobehavioral processes underlying these relationships. The set of mouse models identified in this study can be used to characterize these addiction‐related processes further.

## Graphical abstract

We present major results from a tremendously broad screen of drug‐naïve behavioral traits in mice from the knockout Mouse Project, resulting in the identification of multiple null alleles predicted to cause abnormal drug and alcohol intake. We validated these predictions using oral drug self‐administration. In this study, we show that abnormal drug and alcohol intake phenotypes have nonlinear but significant complex associations with underlying behavioral traits such that extreme effects on various traits can lead to similar outcomes on the same drug intake trait and different outcomes on others.

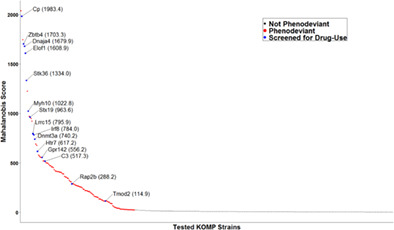

**Abbreviations**
2BC: Two‐bottle choice
B6NJ: C57BL6/NJ
BEC: Blood ethanol concentration
DID: Drinking in the dark
DRG: Drug‐related genes
EtOH: Ethanol
FDR: False discovery rate
GO: Gene ontology
GSEA: Gene set enrichment analysis
GWAS: Genome‐wide association studies
IMPC: International mouse phenotyping consortium
KEGG: Kyoto encyclopedia of genes and genomes
KO: Knockout
KOMP: Knockout mouse project
MA: Methamphetamine
MESH: Medical subject headings
PCA: Principal component analysis
QTL: Quantitative trait loci
SUD: Substance use disorders
TST: Tail suspension test

## 1 INTRODUCTION

Substance use disorders (SUDs) are highly heritable and widely prevalent brain diseases^1^ that manifest themselves both behaviorally and physiologically.^2^, ^3^ Currently, over 20 million people ages 12 and up are suffering from a SUD in the United States,^4^ and drug and alcohol use costs Americans more than $700 billion and contributes to 570,000 deaths per year.^2^, ^5^

Despite extensive efforts to identify and characterize mechanisms driving substance use, few pharmacotherapeutic treatments exist.^3^ This may be due, at least partly, to a historical emphasis on the deep characterization of a few well‐known biological mechanisms influencing substance use rather than on the discovery of novel and perhaps unexpected genetic mechanisms influencing substance use. Due to the conservation of many aspects of the addiction‐related reward circuitry across species,^6^, ^7^ it is possible to leverage the exquisite resources of mouse genetics to discover new biological mechanisms of addiction risk behaviors.^6^, ^7^

Genetic and genomic screens have been previously employed in mutant mouse strains to identify novel addiction risk mutations. A major challenge in these studies is that they require a separate drug‐exposed cohort of mice to avoid the effects of drug exposure on subsequent physiology and behaviors.^8^ In more recent high‐throughput, discovery‐based approaches conducted by the International Mouse Phenotyping Consortium (IMPC), large‐scale screens which employ a single unified test battery were found to efficiently characterize behavioral and physiological phenotypes of single‐gene C57BL/6NJ (B6NJ) KO strains.^9^ The targeted genes were selected by prioritizing genes for which no mutant alleles existed or were nominated by domain experts.^10^, ^11^, ^12^ We suggest that the large‐scale behavioral screen, that includes drug use predictive phenotypes as part of the test batteries might efficiently identify subsets of mutants to further test for drug exposure phenotypes.

Many risk factors for and consequences of drug use and SUDs are associated with other predisposing drug‐naïve phenotypes, personality traits and co‐occurring psychological conditions in humans, including anxiety, depression, impulsivity and novelty‐seeking.^13^, ^14^, ^15^, ^16^ Using mouse behavioral tests, it is possible to precisely model many aspects of these predisposing or co‐occurring traits.^16^, ^17^, ^18^ Previous rodent studies have shown that predisposing drug‐naïve phenotypes, which can be assayed using approach‐avoidance tasks, historically utilized “behavioral despair” assays and novelty‐seeking tasks, can be used to predict future drug‐related behavioral phenotypes, such as conditioned place preference, sensitization and self‐administration.^19^, ^20^, ^21^ Additional studies using inbred mouse populations have revealed shared genetic mechanisms driving predisposing drug‐naïve phenotypes and drug‐related behavioral phenotypes across distinct drug classes.^22^, ^23^ However, despite these efforts, many genes underlying the shared genetic variation among drugs, alcohol and predisposing drug‐naïve phenotypes remain unknown. In the present study, we exploited these relationships, using five behavioral assays which identified 10 drug‐naive phenotypes. We hypothesize these phenotypes will be predictive of drug‐related behaviors. The work was performed in two cohorts with extreme mutants in cohort 1 being selected as having a difference from control on at least 1 of 10 drug‐naïve behavioral phenotype and in cohort 2 the extreme mutants were selected using Mahalanobis distance, a multivariate analysis, of the eight phenotypes (tail suspension was removed from cohort 2). This resulted in the identification of 33 unique genes to test for drug‐related phenotypes. We chose to evaluate whether these strains exhibited altered consumption or preference for alcohol, methamphetamine and nicotine (only cohort 1).

## 2 MATERIALS AND METHODS

### 2.1 Animal care and husbandry

Mice of both sexes were used in all experiments and maintained in a climate‐controlled room under a standard 12:12 light–dark cycle (lights on at 0600 h and off at 1800 h). They were provided free access to food (NIH315K52 chow, Lab Diet 6%/PM Nutrition, St. Louis, MO, USA) and acidified water with vitamin K supplementation unless indicated otherwise. All husbandry, procedures and protocols were approved by The Jackson Laboratory (JAX) Animal Care and Use Committee and were conducted in compliance with the National Institutes of Health Guidelines for Care and Use of Laboratory Animals. All details for housing, experimental design and testing conditions can be found at https://phenome.jax.org/projects/JaxKOMP-LAP/animal?static=True.

We followed JAX's rigorous genetic quality control and mutant gene genotyping programs so that the genetic background and integrity of the mutation were maintained. In addition to the quality control JAX employs to maintain the integrity of the background strains, these quality control measures were also employed to maintain the integrity of the genotypes of strains with identified molecular mutations. For example, all KO strains used in this project were created using B6NJ (RRID: IMSR_JAX:005304) embryonic stem cells such that no flanking DNA differs from controls and mutants. Similarly, all endonuclease‐modified strains used have no flanking DNA, which differs from control strains. In addition, we received all strains for our screens directly from JAX production colonies at wean, ensuring that all strains tested met requirements for rigorous genetic quality control of background and mutations.

### 2.2 Overview of behavioral phenotyping procedures

The Knockout Mouse Project (KOMP) phenotyping center (KOMP2, RRID: SCR_017528) at JAX was established in 2011 to generate and phenotype 833 single‐gene knockout (KO) mouse strains. The KOMP2 pipeline includes measures of physiological, behavioral and biochemical characteristics and the implementation of a standardized battery of analyses to characterize the effects of gene KOs. We performed analyses on measured traits using the R/PhenStat Bioconductor package (v 1.0.0).^24^ PhenStat (RRID: SCR_021317) is built on a linear mixed‐effects model where the date of the test is considered the random effect with sex, genotype and the interaction of sex and genotype information as fixed effects terms. Missing values were ignored.

Using data collected from the KOMP2 pipeline, we undertook two rounds of prioritization of KO strains for phenodeviance on predisposing drug‐naïve phenotypes and selected mice from each round for subsequent drug‐use evaluation, the first in 2014 and the second in 2017. Within each cohort, mice underwent the full battery of tests of biological and behavioral endpoints, including assays such as glucose tolerance, open field and light–dark.^9^ Tests were arranged in a fixed order by perceived stressfulness to minimize potential carry‐over effects (Table S1). In addition, all runs within the phenotyping pipeline were conducted in a sex‐specific manner, for example, each run consisted exclusively of males or females. Protocols for all tests can be found at https://phenome.jax.org/projects/JaxKOMP-LAP/protocol.

### 2.3 Cohort #1: Relationship of predisposing drug‐naive phenotypes to drug‐intake phenotypes: Phenodeviance, two‐bottle choice and principal component analysis

Within the KOMP2 resource, 221 KO strains had undergone behavioral phenotyping at the time of the first screen in 2014. These strains were matched with BNJ controls and tested on a broad behavioral phenotyping pipeline that included as part of the KOMP2 project (Table S1) five behavioral assays (tail suspension https://www.mousephenotype.org/impress/ProcedureInfo?action=list&procID=160&pipeID=7, acoustic startle https://www.mousephenotype.org/impress/ProcedureInfo?action=list&procID=744&pipeID=7, open field https://www.mousephenotype.org/impress/ProcedureInfo?action=list&procID=502&pipeID=7 light/dark https://www.mousephenotype.org/impress/ProcedureInfo?action=list&procID=159&pipeID=7 and hole board https://www.mousephenotype.org/impress/ProcedureInfo?action=list&procID=156&pipeID=7) that define 10 predisposing drug‐naïve phenotypes (Table S2) previously shown to predict drug‐related behaviors in mice.^17^, ^18^, ^25^, ^26^, ^27^ Links to protocols for the tail suspension tests,^27^ acoustic startle,^28^ open field,^21^ light/dark^17^ and hole board^29^ match the SOPs used at the time of testing (2014).

#### 2.3.1 Detecting predisposing drug‐naïve phenodeviance

We rankZ transformed the data from 221 KO strains and analyzed it by the linear mixed model within PhenStat (v 1.0.0).^24^, ^30^ We found 143 significantly phenodeviant strains from B6NJ controls (*p* < 0.05) on at least 1 of the 10 chosen predisposing drug‐naïve phenotypes and identified them as extreme strains. We prioritized strains with multiple significant predisposing phenotypes for further testing for drug‐use phenotypes; however, testing was restricted to strains available at the time of the study. Of the 143 strains (representing 125 genes) that were phenodeviant on at least one of the predisposing traits (Table S3), 19 were selected for further testing because they existed as established live colonies (rather than frozen embryos) and were thus available for test cohort production. The 19 strains from KOs of the following genes all on the C57BL/6NJ background were tested: *Btg2, C1qa, C9, Cfb, Cp, Dnajb3, Dnase1l2, Epb41l4a, Far2, Gipc3*, *Hdac10*, *Hspb2, Htr1a, Il12rb2, Lpar6, Parp8, Pitx3, Pnmt, Rilpl2*. All strains were homozygous for their gene deletions. All mutants were tested relative to sex and age‐matched control B6NJ mice.

#### 2.3.2 Two‐bottle choice assay to evaluate drug‐related phenotypes

Mice from the 19 KO strains selected for the two‐bottle choice (2 BC) protocol were obtained from the JAX Repository and transferred to the JAX housing and phenotyping facility. Mice were group‐housed, with no more than five of the same sex, in duplex polycarbonate cages before testing. Using a 2 BC assay, we then determined drug‐use phenotypes by defining substance use for ethanol (EtOH, *n* = 321), methamphetamine (MA, *n* = 320), or nicotine (*n* = 310) in mice from each of these strains (total 951). Cohort #1: *n* = 2–11 (average 7.25) per drug/sex for KO and *n* = 30–39 (average 33) per drug/sex for control (see Table S4 for each sample sizes and RRID of each KO). There were low numbers for *Pitx3* (2) and *Lpar6* (3) for ethanol phenotyping and *Cfb* (2) for nicotine phenotyping.

At a minimum of 1 day before testing, we rehoused the mice individually in duplex polycarbonate cages with a single Shepherd Shack® and Nestlet® for the duration of testing. We kept the single housing time minimal to reduce the effects of social isolation.^31^ The 2 BC protocol was adapted from one previously published^32^ to test three different drugs at varying concentrations: EtOH (3%, 6%, 12% and 15%), nicotine (10, 20, 40 and 80 mg/L) and MA (10, 20, 40 and 80 mg/L). All three drugs were diluted in sterilized acidified (pH 2.5–3) water. The nicotine solution also contained 20 g/L saccharin sodium salt hydrate to mitigate the bitter taste. For each drug, mice were exposed to both a tube of water and a tube of the drug at the indicated concentration. Each concentration was tested for 2 days before switching to the next concentration which was two times the prior concentration. Individual mice were exposed to only one drug for testing. Six data points were collected for each mouse, the weight of the water bottle and drug bottle at each of three different concentrations. From these data we calculated measures of drug preference and drug consumption. Drug preference was defined as the volume of drug consumed/total fluid volume consumed (drug + water), whereas drug consumption is defined as the amount of drug consumed (mL of drug consumed × g/mL drug)/kg body weight. We also analyzed water intake and per total fluid intake as these measures facilitate the interpretation of drug preference and consumption outcomes. Water intake is the total volume of water ingested over the specified time frame, whereas total fluid intake is the total volume of water plus drug solution consumed over the specified time frame. Due to strain availability at the time of testing, we tested 16 of the 19 strains with all three drugs; EtOH data is missing for one strain [*C9*], while nicotine and MA data are missing for two strains [*Lpar6* and *Pnmt*].

We then applied a repeated‐measures ANOVA to each of the 2 BC phenotypes to assess the strain × sex × concentration effects and evaluate strain and strain × sex effects. After fitting each model, we obtained the least‐squares mean difference between each KO relative to the B6NJ controls. We tested for higher‐order effects and worked our way down, thus we can detect a significant interaction effect without significant main effects. We used a threshold of false discovery rate (FDR) < 0.05 to test for significance of effects in the model and a strain was determined significant if strain was included anywhere in the model.

#### 2.3.3 Principal component analysis to define relationships among phenotypes

To investigate the underlying shared correlation structure across drug naïve and drug‐use behaviors, we conducted a principal component analysis (PCA).^33^ All genotype difference estimates were subjected to a Van der Weerden (RankZ) transformation and PCA in R (V 3.4.4) using factoextra _1.0.5, fviz_pca_biplot (RRID:SCR_016692).^34^ We extracted the first two principal components (PCs) to assess the relationships among the 10‐predisposing drug‐naïve phenotypes, the six drug‐use 2 BC traits (consumption, preference x three drugs) and the six liquid consumption 2 BC traits (water, total liquid intake × three drugs). We then performed a PCA biplot clustering using the effect sizes across predisposing drug‐naïve phenotypes and 2 BC traits. Because PCA can only be conducted on complete data sets and because *C9, Lpar6* and *Pnmt* data were incomplete, we only analyzed 16 of the 19 KO strains by PCA.

### 2.4 Cohort #2: Relationship of predisposing drug‐naive phenotypes to drug‐use phenotypes: Multivariate outlier detection, two‐bottle choice and drinking in the dark assay

We took advantage of the ongoing KOMP program for our second test cohort. At the time we identified our second validation cohort, a total of 401 KO strains had undergone behavioral phenotyping. These 401 strains included all 221 of the strains that were included in the first cohort selection (Table S5). Mice from these strains were matched with temporally local B6NJ controls and tested on four of the five behavioral assays (acoustic startle, open field, light/dark and hole board) that define 8 of the 10 predisposing drug‐naïve phenotypes that were used in Cohort #1. The tail suspension assay, including measures of time immobile and latency to immobility phenotypes, was dropped from KOMP2 testing and was excluded from the strain identification as this missing data would have greatly reduced the number of strains with sufficient data for the Cohort #2 analysis.

#### 2.4.1 Mahalanobis distance to identify predisposing drug‐naïve phenotypes

In this cohort, we first used the R/Phenstat Bioconductor package (v 1.0.0)^24^ for modeling the association between trait and genotype. We then performed a rankZ transformation and input the transformed genotype effect estimates to Mahalanobis distance calculations. We used the Mahalanobis distance to identify which of the 401 KO strains were phenodeviant across the eight predisposing phenotypes. We chose this approach because our initial cohort revealed non‐uniform, multidimensional relations underlying the drug use and their predisposing drug‐naïve phenotypes. Mahalanobis identifies multivariate outliers strains by calculating the distance from the centroid, representative of control strain values, in a multidimensional space.^35^ The centroid is defined as the intersection of the mean of the variables being assessed. The Mahalanobis distance follows a chi‐squared (𝜒^2^) distribution, a common gamma distribution used in inferential statistics to evaluate statistical significance.^35^ This was used to create one score representing the combined phenodeviance across all eight predisposing phenotypes. Phenodevience was defined as a combined Mahalanobis score that was significantly extreme based on 𝜒^2^; using this criterion, we identified 123 of the 401 strains as significantly phenodeviant from the B6NJ controls.

Of these 123 phenodeviant strains identified, our goal was to rederive and test further the most extreme 25 strains as defined by their highest scores. Due to the availability of sperm, the success of in vitro fertilization, and the ability to produce viable cohorts from each strain, we tested 13 of these most extreme phenodeviant strains. We also included *Tmod2* and *Rap2b* KO strains, which were phenodeviant as determined by the Mahalanobis distance calculations but not in the top 25 most phenodeviant, due to expert recommendation. The 15 strains (16 including *Cp* which was tested again), all on a C57BL/6NJ background were KO for the following genes: *Elof1, Stk36, Myh10, Dnmt3a, Cp, Zbtb4, Dnaja4, Irf8*, *Htr, Gpr142, C3, Stx19, Lrrc15, Rap2b, Tmod2*. These strains were obtained from the JAX Breeding and Rederivation Services and transferred to the JAX housing and phenotyping facility, where they were bred to testable cohort sizes through pair and trio mating. Viable homozygous null strains were bred using −/− × −/− breeding pairs or trios (1 M, 2F), whereas lethal homozygous null strains *Elof1, Myh10, Dnmt3a* and *Rap2* were bred using +/− × +/− breeding pairs or trios.

#### 2.4.2 Methamphetamine two‐bottle choice

We tested mice for drug‐use phenotypes in Cohort #2 using the MA 2 BC since we wanted to continue rapidly screening mice for MA‐use phenotypes. We tested mice for MA use phenotypes using the same protocol described for Cohort #1, including the same four concentrations of MA (10, 20, 40 and 80 mg/L). For MA in Cohort #2 *n* = 3–9 (mean 7.4) per sex for KO and *n* = 19.5 per sex for control, the individual number of mice tested for each KO strain is indicated in Table S6 and S7, and 275 were tested in total. We did not test for nicotine oral self‐administration because we failed to find any effects using our nicotine protocol in Cohort #1.

#### 2.4.3 Ethanol drinking in the dark protocol

We used the EtOH drinking‐in‐the‐dark (DID) assay to identify mice for the more translationally relevant binge drinking phenotype. Although preference drinking (EtOH 2 BC) is a widely used and valid partial model for alcohol use,^36^, ^37^ the DID protocol has improved relevance as a model for binge‐like drinking.^38^, ^39^, ^40^ Furthermore, it has been well documented that there is significant comorbidity of alcohol and MA use^32^ however the newer evidence suggests that binge drinking has a significantly higher comorbidity rate and is a better predictor for MA use than moderate drinking.^41^, ^42^ We, therefore, chose to use these two protocols to phenotype our extreme single‐gene KO strains for EtOH binge drinking effects and strong multi‐drug effects that have translational relevance to human patterns of MA and alcohol use.

We tested 268 adult (8–24 week old) mice using the previously published EtOH DID protocol.^38^, ^39^, ^40^ This protocol has been refined to induce mice to drink to levels of intoxication (~100 mg/dL).^43^, ^44^ For Cohort #2 DID: *n* = 5–8 (average 7.7) per sex for KO and *n* = 18 per sex for control. The actual number of mice tested for each KO strain is indicated in Table S7. The EtOH DID protocol is a four‐day, limited‐access protocol in which EtOH is available during a time in the circadian cycle when mice are behaviorally active (nocturnal) to induce binge drinking behaviors that lead to intoxication on the final day. To determine if a KO strain was considered significantly different than controls, we applied a repeated‐measures ANOVA to EtOH consumption across the 4 days of the DID protocol to assess the strain × sex effects, in addition to assessing strain effects. Following the model fit, we obtained the least squares mean difference between each KO relative to the B6NJ control. We used a threshold of FDR <0.05 to determine the significance of terms in the model. Of particular interest were strain, and strain × sex effects.

#### 2.4.4 Blood collection and blood ethanol concentration analysis

We collected a minimum of 50 μL of blood into a micro container (VWR, Radnor, PA USA cat.# VT365956) immediately following ethanol removal on the final day of the DID protocol. We centrifuged blood samples at 13,300 RPM for 11 min and pipetted serum into separate Eppendorf tubes on dry ice within 1 h of collection. We transferred the serum samples to a − 80°C freezer for storage within 3 h of collection. We determined blood ethanol concentration (BEC) using an enzymatic rate method^45^ with the Ethyl Alcohol Assay (Beckman Coulter, Brea CA, USA) run on a Beckman DXC biochemical analyzer (RRID:SCR_019633). We analyzed BEC using a two‐way ANOVA and evaluated significant differences between each KO relative to the B6NJ control using Tukey's Honestly Significant Difference test.

### 2.5 Functional annotation of candidate genes

To evaluate whether and how the genes identified in our knockout analysis might be involved in addiction‐related traits, we searched for genetic and genomic evidence to identify plausible biological mechanisms in which the genes could have affected drug‐use phenotypes. To accomplish this, we performed a functional annotation of all the statistically significant genes in cohort 2 using GeneWeaver (RRID:SCR_003009).^46^ We also conducted a systematic search of the genes which altered 2 BC phenotypes to determine whether they were represented in previous curated genomic data sets from studies of humans, mice and rats. Among the data resources used in the analysis were the following: (a) Medical Subject Headings (MeSH) (RRID:SCR_004750)^47^ related to drugs or addiction, (b) Gene Ontology (GO) (RRID:SCR_006447)^48^, ^49^ terms related to drugs or addiction, (c) Quantitative Trait Loci (QTL)^50^ gene sets related to drugs or addiction, (d) Kyoto Encyclopedia of Genes and Genomes (KEGG) (RRID:SCR_001120)^51^, ^52^, ^53^ pathways related to addiction and alcoholism, (e) Neuroinformatics Framework Drug‐Related Genes (DRG) (RRID:SCR_003330)^54^ and (f) Genome‐Wide Association Studies (GWAS)^55^ of alcohol and substance use related traits. In addition, we performed a Gene Set Enrichment Analysis (GSEA, RRID:SCR_003199, accession date February 4, 2020 and August 5, 2020^56^ on the genes from cohorts 1 and 2.

## 3 RESULTS

### 3.1 Detecting predisposing drug‐naïve phenodeviance in Cohort #1

**FIGURE 1:**
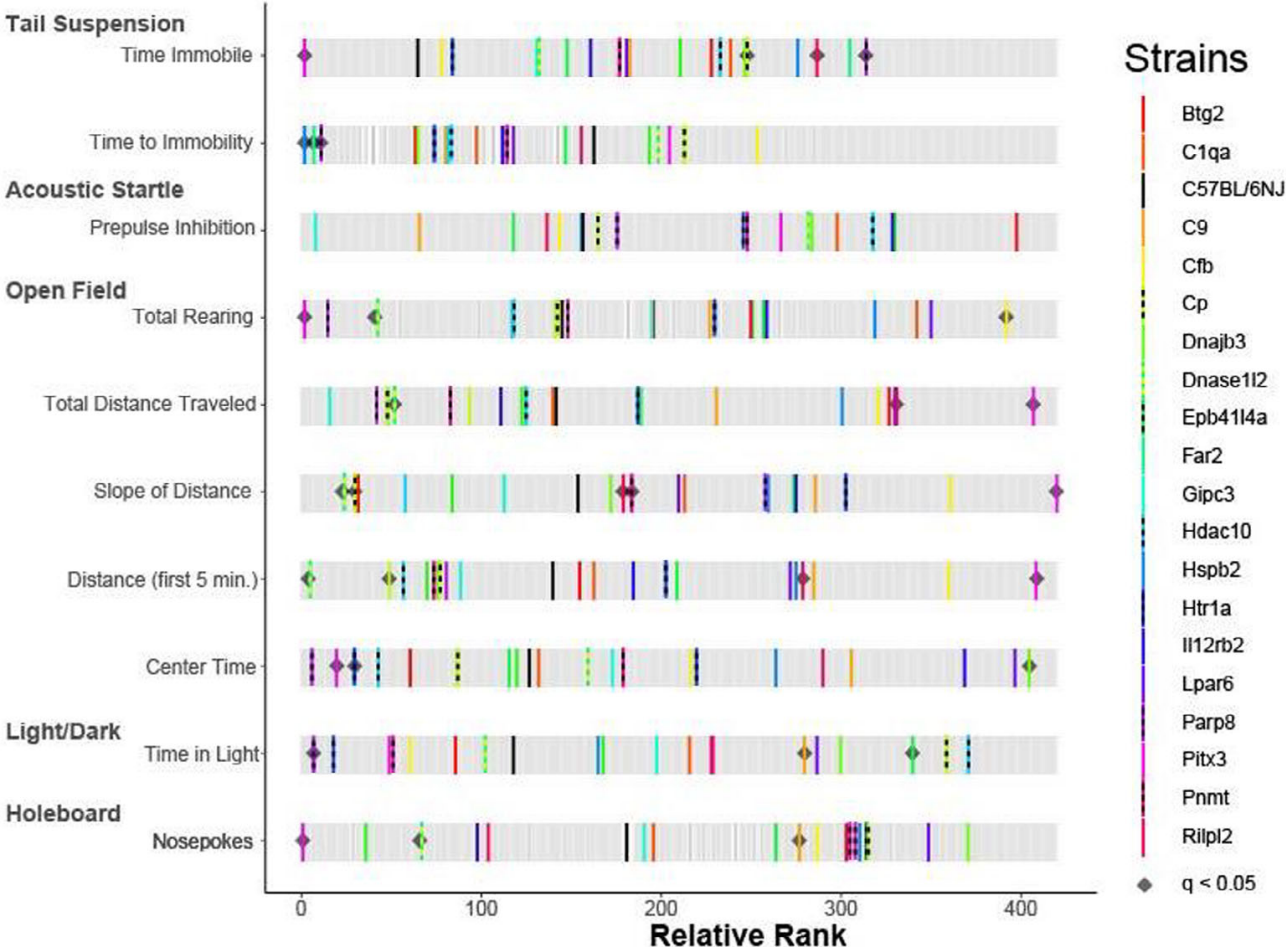
Phenotypic variation in predisposing drug‐naïve phenotypes from the 19 phenodeviant strains identified in Cohort #1. Of the 221 strains tested in 2014, 143 strains were phenodeviant in at least one predisposing, drug‐naïve phenotype and 19 were established colonies and tested further for drug‐use phenotypes using the two‐bottle choice assay. The Rank Z graph displays where the 19 strains fall in the range of the 221 strains measured for each of the 10 predisposing, drug‐naïve phenotypes. Thick black bars represent C57BL6/NJ controls. Colored bars represent strains identified as phenodeviant and predictive of addiction risk phenotypes. Gray bars represent the ranked genotype effects for each measure calculated across all KO strains tested in Cohort #1. Black diamonds indicate KO strains from initial screening which remained significantly phenodeviant when accounting for multiple testing corrections (q < 0.05).

Of the 221 KO strains tested, 143 (64.7%) were phenodeviant as defined by at least 1 of the 10 predisposing drug‐naïve phenotypes being significantly different (*p* < 0.05) from B6NJ controls. Of these 143 phenodeviant strains, we further analyzed 19 for drug‐intake phenotypes (the remaining 124 strains were no longer available for testing as they had been cryopreserved and the live strain no longer maintained). The rankZ results of the 10‐predisposing drug‐naïve phenotypes obtained from the battery of five behavioral tests for these 19 strains are shown in Figure 1 and in Table S3, and samples sizes of KOs tested by KOMP in Table S8. Although the 19 KO strains chosen for drug‐exposure phenotyping were limited to available live colonies, they nonetheless represented the breadth of phenodeviance observed across the 143 strains and most predisposing drug‐naïve traits, with the exception of PPI, for which no statistically phenodeviant strains were available and TST for which only strains with low latency to immobility were tested.

### 3.2 Two‐bottle choice assay to determine drug‐use phenotypes

**FIGURE 2:**
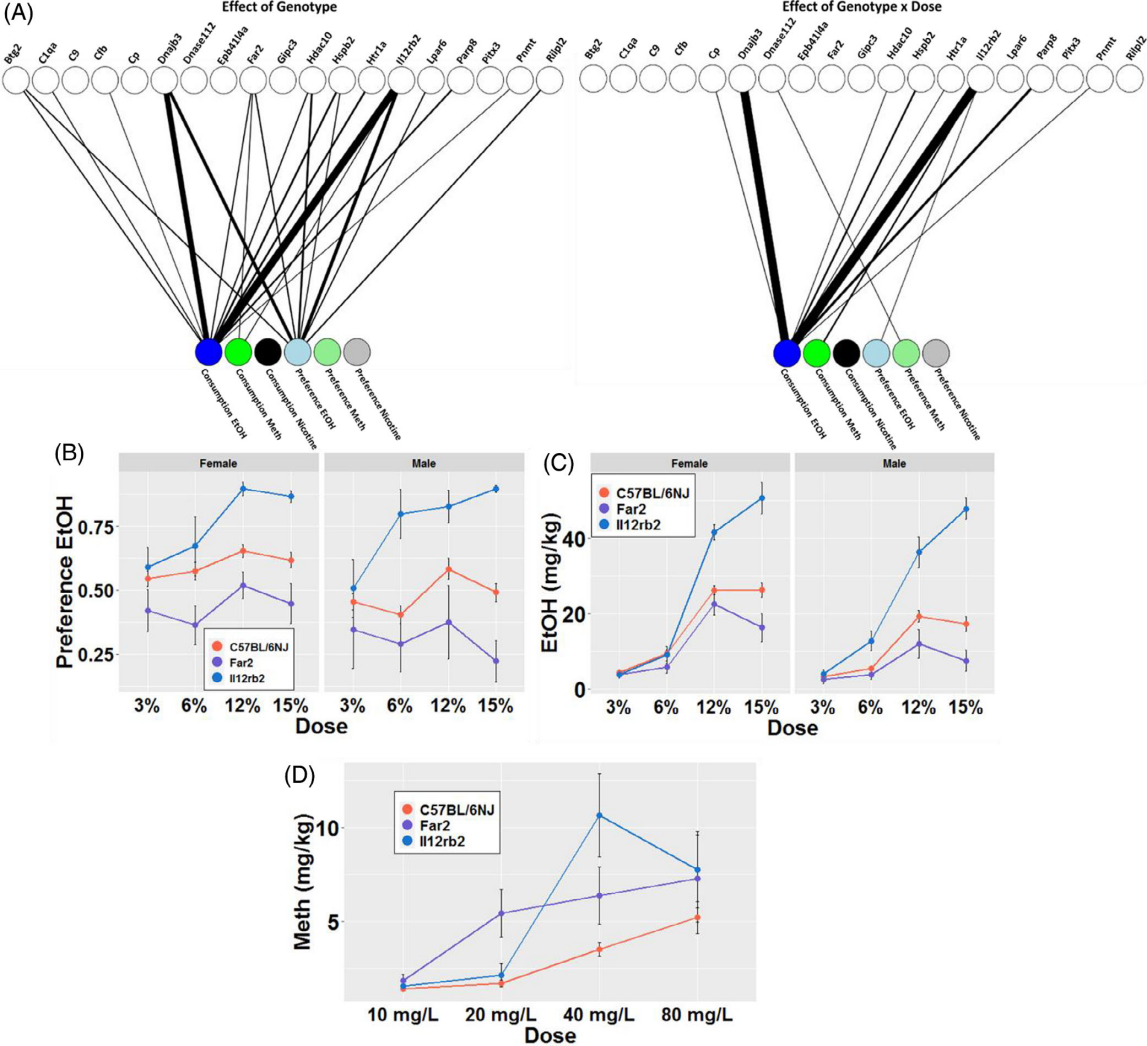
Representative data of the shared associations of gene deletion mutations with alcohol, methamphetamine and nicotine use phenotypes. (A) Bipartite graphs depicting significant effects of strain and strain × concentration for consumption and preference of alcohol (EtOH), nicotine and methamphetamine (MA). Significant associations are represented by the thickness of the edge connecting the two nodes. Edge weights are inversely proportional to the—log10 *p*‐value of the association. (B) Dose–response curve depicting the effect of *Il12rb* and *Far2* deletions on EtOH preference. (C) Dose–response curve depicting the effect of *Il12rb* and *Far2* deletions on EtOH consumption. (D) Dose–response curves depicting the effect of *Il12rb* and *Far2* deletion on MA consumption. Data are shown as mean ± SD (FDR <0.05). Full data for each mutant is available in the supplement.

We tested these 19 single‐gene KO strains using the 2 BC test for drug consumption and preference for EtOH, nicotine and MA for a total of six drug‐use phenotypes (Table S4). The results of the three‐drug exposure experiments are presented in Tables S9–S11, and statistical tests in Table S12. Based on significant effects by strain or strain × concentration, we found that 15 of the 19 strains showed at least one significant drug‐use effect. We observed 14/18 strains tested for ethanol (*Cp* wasn't tested) and 3/17 strains tested for MA/nicotine (*Pnmt* and *Lpar6* were not tested) showing significant differences from controls. Two genes, *Il12rb* and *Far2* each showed significant differences from controls for three of the six phenotypes, the most of all 19 strains tested. In contrast, *Epb41l4a, Pitx3, Gipc3* and *C9* showed no significant differences from controls in any of the six drug‐use phenotypes tested (Figure 2A), with the caveat that *C9* was not tested on ethanol.

Oral EtOH‐use phenotypes (consumption and preference) showed the highest numbers of significant associations; five strains showed overall strain effects, and eight showed strain × concentration effects for at least one of the two EtOH phenotypes (Figure 2A). We further found a wide range of outcomes exemplified by *Il12rb2* and *Far2*. Deletion of *Il12rb2* resulted in an increase in both EtOH preference (F_strain (1,90)_ = 35.7, FDR = 8.63E−07) and consumption (F_strain (1,90)_ = 88.48, FDR = 9.28E−14) compared with B6NJ controls (Figure 2B). In contrast, deletion of *Far2* resulted in a decrease in both EtOH preference (F_strain (1,87)_ = 12.31, FDR = 3.40E−3) and consumption (F_strain (1,87)_ = 9.3, FDR = 6.40E−3) as compared with controls (Figure 2C). Across the strains, females (F_sex (1,318)_ = 18.16, P = 2.68E−05) exhibited higher levels of preference and consumption for EtOH than males (F_sex (1,318)_ = 34.03, P = 1.34E−08), however only one strain showed a significant strain x sex interaction *Hdac10* for total ethanol consumption (F_strain x sex (1,90)_ = 10.41, FDR = 0.033).

For MA, in which we tested 17 of the 19 single‐gene KO strains, we found that three strains (*Il12rb2*, *Far2* and *Dnase1l2*) showed significantly altered preference or consumption of MA (Figure 2A). Unlike for EtOH outcomes, we did not detect sex effects for MA phenotypes. Interestingly, while *Il12rb2* and *Far2* deletions showed increased and decreased responses, respectively, for oral EtOH self‐administration phenotypes compared with controls (Figure 2D), *Il12rb2* and *Far2* deletions both led to significantly increased consumption of MA (*Il12rb2*, F_strain (1,71)_ = 9.88, FDR = 2.19E−02; *Far2*, F_strain (1,69)_= 10.59, FDR = 2.19E−02) (Figure 2C).

Using the 2 BC screening, we were unable to detect any KO strains with significantly altered oral nicotine self‐administration phenotypes (Figure 2A). This could be due to many factors including the aversive taste of oral nicotine, a confounding effect of saccharin with the nicotine, or other experimental parameters related to the substance, which has been less well studied in laboratory mice than ethanol for example.

### 3.3 Principal component analysis to define relationships among phenotypes

**FIGURE 3:**
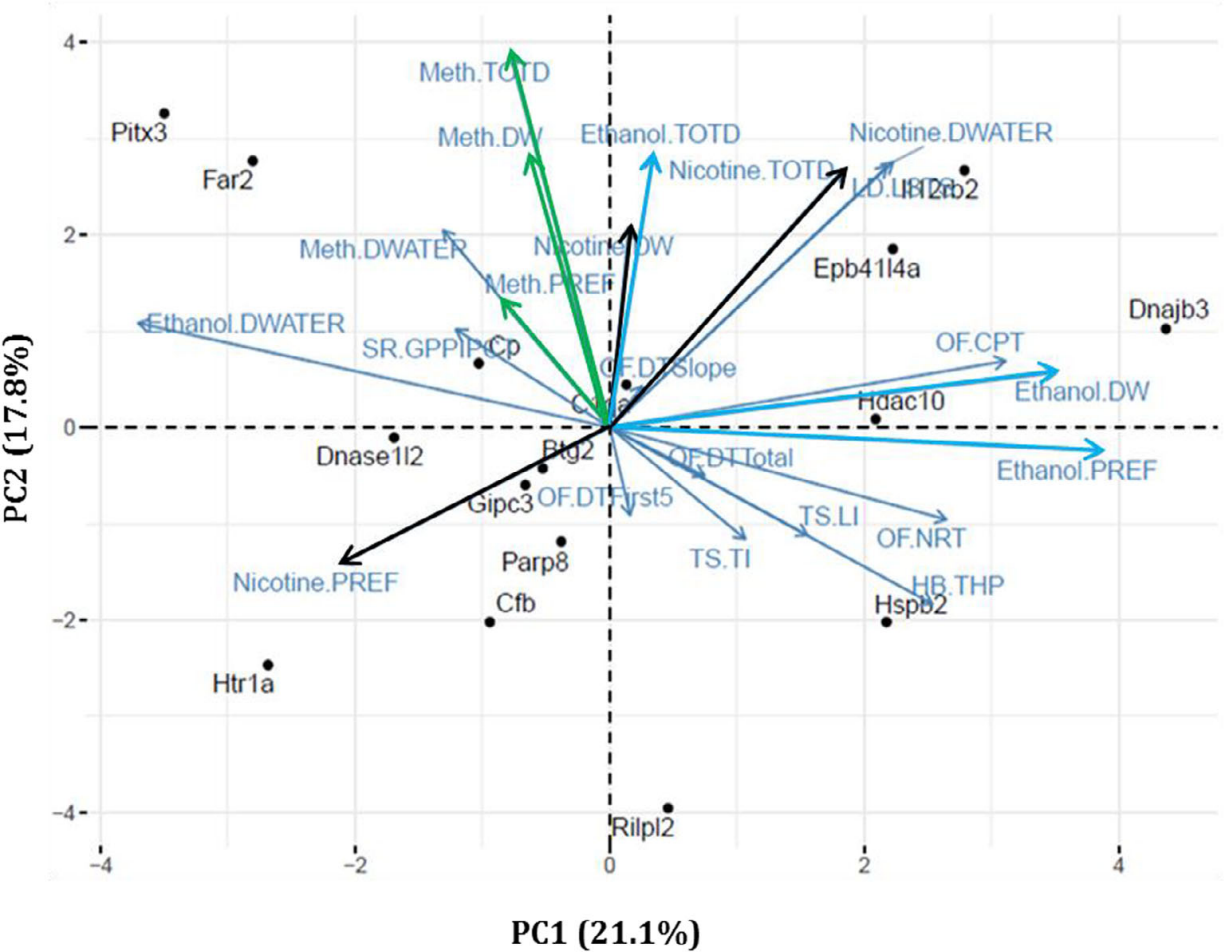
Shared relationships among predisposing drug‐naïve behaviors with drug‐use and liquid intake phenotypes. Principal components analysis was used to assess shared variance among predisposing drug‐naive behavioral traits and drug‐use and liquid intake phenotypes. Each point represents a KO strain, while arrows represent each of the analyzed traits. Analysis was conducted on all 16 strains tested on all behavioral phenotyping measures. Nicotine is black, dark blue is EtOH and green is MA. Light blue refers to water consumption or baseline behaviors. *Far2* and *Il12rb* from Figure 2 have been circled to highlight them. Ethanol‐DW, ethanol consumed; Ethanol‐DWATER, water drinking ethanol; Ethanol‐PREF, preference ethanol; Ethanol‐TOTD, total drinking ethanol; HB, hole board; LD‐LSTS, light/dark time spent in light; Meth‐ DWATER, water drinking meth; Meth‐DW, meth consumed; Meth‐PREF, preference meth; Meth‐TOTD, total drinking meth; Nicotine‐ DWATER, water drinking nicotine; Nicotine‐DW, nicotine consumed; Nicotine‐PREF, preference nicotine; Nicotine‐TOTD, total drinking nicotine; OF‐CPT, center permanence time; OF‐DTFirst5, distance traveled first 5 min; OF‐DTSlope, distance traveled slope; OF‐DTTotal, distance traveled total; OF‐NRT, number of rears total; SR‐GPPIPCT, amplitude percent PPI global; THP, total hole pokes; TS‐LI, latency to immobility; TS‐TI, time immobile.

PCA revealed relationships within and between the 10 predisposing drug‐naïve phenotypes, six drug self‐administration traits (consumption and preference × three drugs) and six liquid intake traits (water‐drinking and total fluid intake × three drugs), a total of 22 phenotypic traits (Table S13). We included liquid intake traits in this analysis to account for variation in total fluid consumption unrelated to the drug. We then performed bi‐plot clustering using the effect sizes across all 22 traits for the 16 KO strains tested for all three drugs (Figure 3).^57^ The PCA reveals that principal components one and two account for 21.1% and 17.8% of the variance respectively, together accounting for ~39% of the variation observed in our 16 strains across the 22 measures. PC1 differentiates KO strains with ethanol drinking from those that display non‐ethanol drinking phenotypes. PC2 relates ethanol‐drinking KO strains to drug‐naïve behavioral profiles of high or low exploration.

The correlations within and between any of the predisposing drug‐naïve phenotypes and drug‐use phenotypes can be assessed using the angle which separates any two vectors (Figure 3). Vectors that fall close to one another (where the angle approaches 0°) are strongly positively correlated; vectors which fall 180° apart are strongly negatively correlated; and vectors which fall 90° apart are independent of one another.^58^ Using these relationships, we can use the PC1 axis to classify our tested strains. For example, we can separate which strains had increased EtOH consuming/preferring phenotypes from those with decreased EtOH consuming/preferring phenotypes. Along the PC2 axis, we found clustering of predisposing drug‐naïve phenotypes, which can be used to divide our strains into different baseline behavioral profiles, that is, “low anxiety” or “exploratory” profiles. In addition, along PC2, we see a close relationship that separates our MA‐consuming strains from our non‐consuming strains. Scores for each strain are obtained by multiplying the PC loadings by the strain means, allowing strains to be plotted in the two‐dimensional space. Strains with high absolute scores on both ends of PC1, such as *Il12rb2* and *Far2*, have strong opposing increased and decreased EtOH preferring/consumption phenotypes, respectively. Additionally, *Il12rb2* and *Far2*, whose variation is similarly explained by PC2, show that while these two strains manifest opposing EtOH phenotypes, both manifest strongly increased MA consumption phenotypes.

### 3.4 Phenotypic deviance based on Mahalanobis distance for predisposing drug‐naïve phenotypes in Cohort #2

**FIGURE 4:**
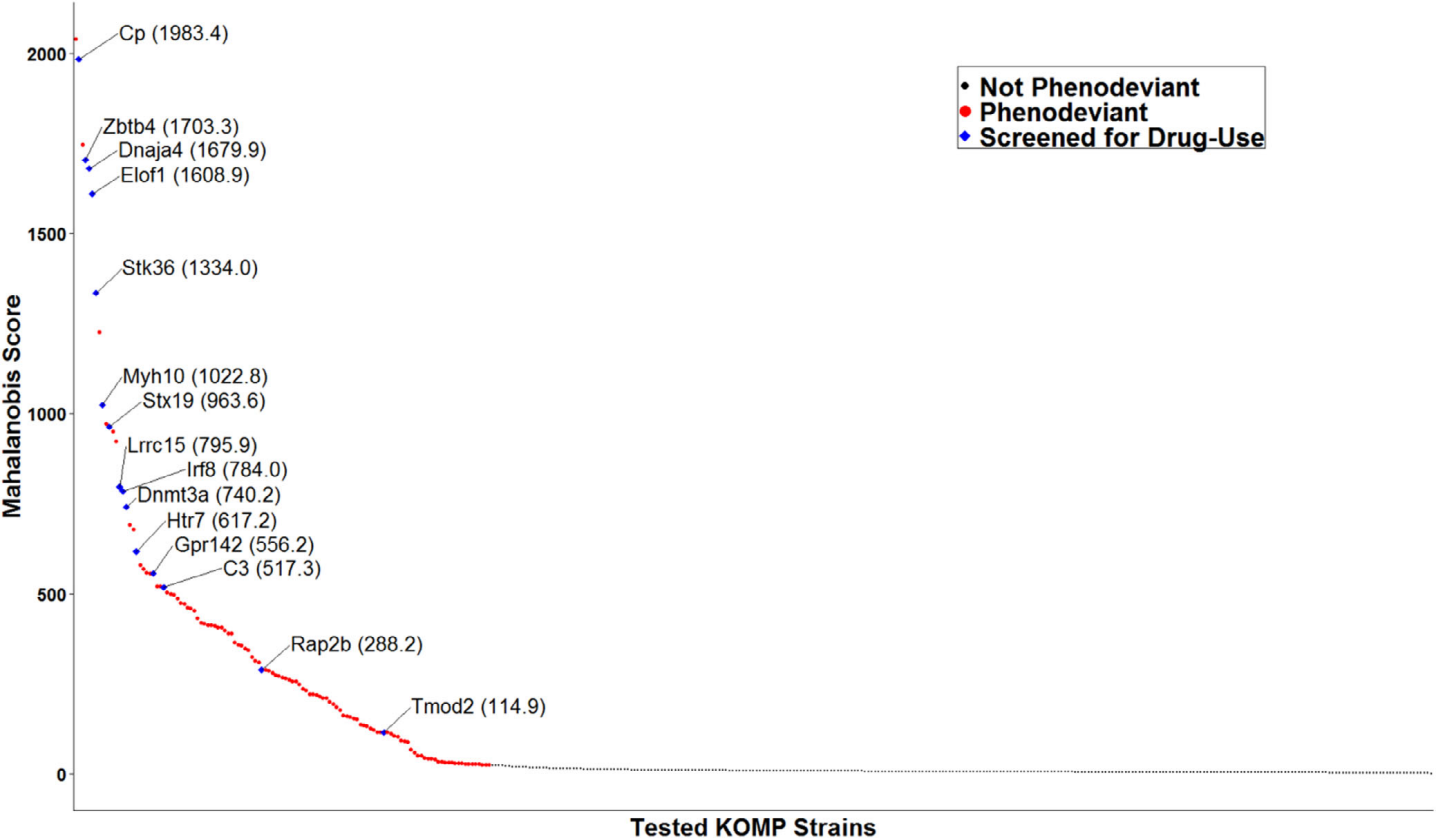
Multidimensional assessment of phenodeviance in drug‐naïve behaviors. Of the 401 strains tested, the 123 KO strains, each indicated as a colored circle in the plot, showed a statistically significant difference from matched C57BL6/NJ controls using Mahalanobis score (FDR <0.05). Red circles represent single gene KO strains that were not rederived; blue points represent strains that were rederived. All blue points are identified by their gene abbreviations and Mahalanobis scores.

We calculated overall phenodeviance using Mahalanobis distances^35^ of effect sizes relative to B6NJ controls. Using this calculation, we represented the phenodeviance from the B6NJ controls across all eight measured predisposing drug‐naïve phenotypes as a single score (Figure 4). Higher scores represent greater overall phenodeviance from controls across all predisposing drug‐naïve phenotypes. In 2017, 401 strains had completed the phenotyping pipeline and were included in the analysis. Results from this analysis indicate that of the 401 single‐gene KO strains tested, 123 strains were significantly phenodeviant, with scores ranging from 24.1 to 2038.9. This range suggests that even within the significantly phenodeviant strains, strains with exponentially greater predisposing drug‐naïve phenotypes and risk factors existed, making them the most likely to manifest drug‐use phenotypes and potentially have multi‐drug effects. Seven of the 19 strains identified as phenodeviant in cohort 1 remained phenodeviant under the larger cohort analyzed by Mahalanobis distance calculation. In total 55 of the phenodeviant strains from cohort one were found to be phenodeviant in cohort 2 (Table S5). We then prioritized the top 25 strains with high Mahalanobis scores (≥517.3) that were also available for rederivation or could be directly obtained to screen for drug‐use phenotypes. Of these, 15 significantly phenodeviant KO strains were successfully rederived or obtained and bred for testing, 13 of which scored in the top quartile of Mahalanobis scores (Figure 4). The gene *Cp* was tested again in cohort 2 after being tested in cohort 1. We also included the *Tmod2* and *Rap2b* KO strains, which were phenodeviant as determined by the Mahalanobis distance calculations but not in the top 25 available strains that were most phenodeviant.

#### 3.4.1 Two‐bottle choice assay to determine methamphetamine‐use phenotypes

**FIGURE 5:**
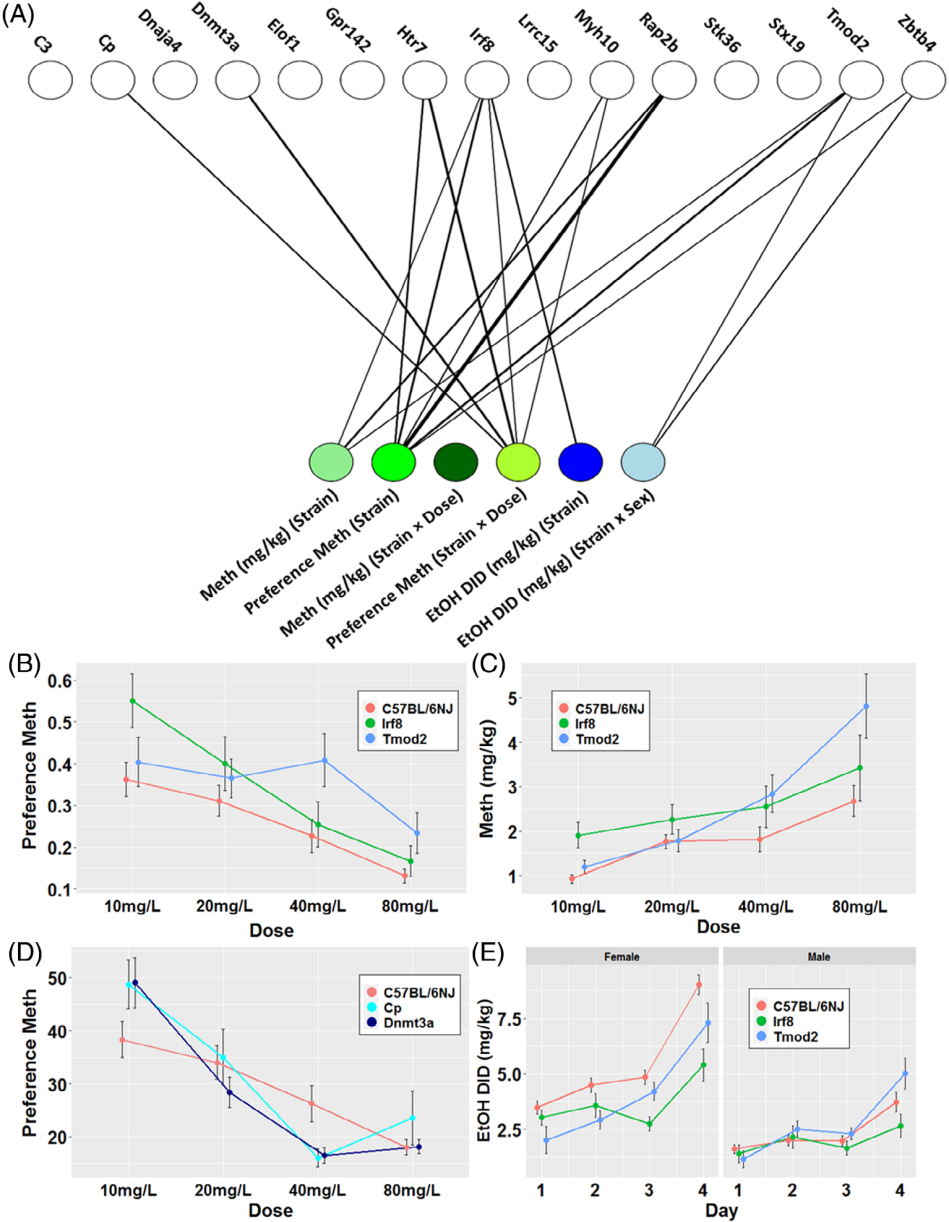
Single‐gene KOs resulting in significant drug‐use effects cohort 2. (A) Bipartite graph displays significant hits across the three measured phenotypes. The graph depicts significant effect of strain with the three green nodes and effects of strain by concentration with the three blue nodes. Significant associations are represented by the thickness of the edge connecting the two nodes. Edge weights are inversely proportional to the—log10 p‐value of the association. (B) Dose–response curve depicting the effect of *Irf8* and *Tmod2* deletions on MA preference. (C) Dose–response curve depicting the effect of *Irf8* and *Tmod2* deletions on MA consumption. Data are shown as mean ± SD for *n* = 16 in each KO group. (D) Dose–response curve depicting the effect of *Cp* and *Dnmt3a* deletions on MA preference. (E) Dose–response curves depicting the effect of *Irf8* and *Tmod2* deletion on EtOH DID consumption. Data are shown as mean ± SD for *n* = 16 in each KO group.

We tested 15 of the strains identified as phenodeviant by Malanhobis testing in Cohort #2 for MA preference and determined that eight strains had significant MA preference phenotypes revealed by bipartite analysis (Figure 5A, Table S6, S14, S15), either manifested through a main effect of strain or strain × concentration (FDR <0.05). Of these eight strains with MA preference phenotypes, only one (*Cp*) was also present in the 19 phenodeviant strains identified by PCA in Cohort #1. Focusing on strains that showed phenotypes in multiple drugs, strains *Irf8, Tmod2* and *Zbtb4* all exhibited significantly increased preference for MA (F_strain (1,51)_ = 12.34, FDR = 4.70E−03), (F_strain (1,51)_ = 15.23, FDR = 2.10E−03), and (F_strain (1,51)_ F = 6.92, FDR = 0.03) respectively, compared with the control strain (Figure 5B). The *Irf8* strain showed a significant interaction of strain × concentration and exhibited significantly increased preference to initial lower concentration of 10 mg/L and 20 mg/L increasing to 55.1 ± 6.5% and 40.0 ± 6.4%, from 31.51 ± 3.3% and 26.64% ±3.0%, respectively and did not display differences from control strains at higher concentrations (F_strain×concentration (3,140)_ = 3.69, FDR = 4.05E−02). Additionally, the *Irf8 and Tmod2* and strains consumed more MA over the 2 BC protocol than the control strains does (F_strain (1,51)_ = 7.42, FDR = 4.40E−02 and F_strain (1,51)_ = 8.92, FDR = 3.24E−02, respectively) (Figure 5C, Table S6, S14, S15).

For the control and most KO strains evaluated, preference for MA trended down as the concentration increased, with 10 mg/L being the concentration with the highest preference compared with water. The control strain had its highest average MA preference, 36.25% ± 4.0%, for the initial 10 mg/L concentration (Figure 5B) and consumed higher percentages of water over all. At the initial 10 mg/L concentration, *Irf8* consumed a higher percentage of MA, 55.1 ± 6.5%, than water. In general, *Irf8* had a higher preference for MA than controls, but differences were largely exhibited in the initial two concentrations (Figure 5B). While most strains had their greatest preference for MA at the lowest concentration of 10 mg/L, similar to controls, we observed a shift in the dose–response curve for *Tmod2*. The *Tmod2* strain had similar preference levels to the initial concentration as controls but had a peak for MA preference at the 40 mg/L concentration (40.9 ± 6.4%) (Figure 5B). Further studies will be needed to determine to what drives the increased preference for methamphetamine.^59^

In our initial prioritization of the KO strains tested by the MA 2 BC assay in Cohort #1, three of the 19 strains (*Il12rb2*, *Far2* and *Dnase1l2*) had altered consumption or preference attributed to the main effects of strain or strain × concentration (FDR <0.05). Sex had no significant main or interaction effect on either MA consumption or preference phenotypes. In the second prioritization, two strains with deletion of genes with distinct biological functions (*Dnmt3a* and *Cp*) resulted in very similar alterations to MA preference in a concentration‐dependent manner. Initially, both *Dnmt3a* and *Cp* exhibited an increased preference for MA at the starting concentration compared with controls (49.1 ± 5.6% and 49.0 ± 5.4%, respectively). We did not observe any differences from controls at 20 mg/L concentration. However, at the higher concentration of 40 mg/L, both strains showed a decreased preference for MA compared with controls (9.7 ± 1.3% for *Dnmt3a* and 10.2 ± 2.0% for *Cp* vs.17.1 ± 2.7% for controls). These results suggest that the deletion of these two genes alters the preference for higher concentrations of MA. While the initial preference was non‐aversive and equal to the percentage of water consumed, it became more rapidly aversive as concentration increased compared with controls (Figure 5D).

#### 3.4.2 Drinking in the dark to determine ethanol‐use phenotypes

Analysis of DID results indicate that three of the 15 phenodeviant strains in Cohort #2 displayed significantly altered EtOH consumption (*Irf8, Tmod2* and *Zbt64*, Table S7, S16). EtOH DID consumption was strongly influenced by sex in all strains (F_sex (1,297)_ = 271.3, *p* < 2.22E−16), but there were no strain × sex interactions. *Irf8* (Figure 5E) had significantly altered EtOH DID consumption across the 4 days of access (F_strain (1,48)_ = 13.38, FDR = 9.48E−03). Additionally, for two strains, *Tmod2* (Figure 5E) and *Zbtb4* (not depicted), EtOH DID consumption was influenced by strain and sex (F _strain×sex (1,47)_ = 8.39, FDR = 4.27E−02) and (F_strain×sex (1,48)_ = 11.72, FDR = 1.91E−02), respectively. Like what was observed in the EtOH DID consumption phenotype, BEC was also influenced by sex in all strains (F_sex (1,264)_ = 33.01, *p* = 2.52E−08). Of the 15 phenodeviant strains chosen for testing in the DID paradigm, no strain resulted in significantly different BEC from the control strain (Table S7).

#### 3.4.3 Genes with drug‐specific or multi‐drug effects

Using a multidimensional assessment of phenodeviance across predisposing drug‐naïve phenotypes in Cohort #2, we identified 15 single‐gene KO strains that we tested for altered patterns of drug‐use phenotypes. Results from MA 2 BC from the second cohort in combination with EtOH DID revealed that eight out of 15 (53.3%) of our identified single‐gene deletions resulted in an altered drug self‐administration phenotype. Three (38%) of these strains (*Irf8, Tmod2* and *Zbt64*) had multi‐drug effects across both drugs (Figure 5A). An interesting observation is that with the second cohort for which we had the ability to rederive more extreme phenodeviant strains, did identify slightly more genes with multi‐drug effects from 12.5% (2/16) to 20% (3/15), but it was only a very small increase in overall percentage. Finally, our overall hit rate for genes that showed both predisposing drug‐naïve phenotypes and drug self‐administration phenotypes in Cohort #1 (79%, 15/19) was higher than the 53.3% observed in cohort 2.

#### 3.4.4 Functional annotation of candidate genes reveals diverse mechanisms of involvement in addiction related phenotypes

Although few if any of the genes we evaluated were recognized as addiction related genes in the literature at the time of testing, 22 genes we identified with both predisposing drug‐naïve phenotypes and drug‐use phenotypes were supported by additional evidence from at least one of the searched databases establishing a prior connection to drug‐related studies, either through expression data, QTL mapping, or connections to drug‐related biological mechanisms (Table S17). In addition to the functional annotation of drug‐related gene sets in GeneWeaver, we assessed the genes with significant effects for overlapping representation in biological pathways. Using GSEA, a systematic search of canonical, KEGG and GO biological or cellular pathways revealed that there was no statistically significant enrichment of any terms within our set of genes displaying drug related phenotypes. However, 12 different GO Biological Process terms did contain multiple genes (Table S18), with GO:0006351 transcription, DNA‐templated containing the most genes (four: *Btg2, Dnase1l2, Hdac10, Pitx3*).

## 4 DISCUSSION

Overall, our data indicate the utility of leveraging the known complex relationships among predisposing drug‐naïve phenotypes and their drug‐related addiction risk phenotypes. In this project, we used and refined our understanding of these relationships in combination with the high‐throughput JAX‐KOMP2 program to identify 33 plausible single‐gene KO strains predictive of drug‐use phenotypes. Of those 33 plausible candidates, 22 (67%) of the single gene KOs significantly altered at least one drug‐use phenotype. Following screening for drug‐use phenotypes, we validated all significant genes through functional annotation for plausible connections and/or mechanisms through which they potentially could have altered drug‐use phenotypes. Further analysis through GSEA indicated no overlapping pathways among our candidate genes that could have possibly affected drug‐use phenotypes, suggesting that these novel candidate genes could represent multiple diverse pathways for roles in drug use.

The strategy we used to identify drug‐use candidate genes using predisposing drug‐naïve phenotypes was successful and circumvented the effects of drug exposure on subsequent physiological testing in the screening program, allowing us to discriminate risk from consequences of drug exposure. An approach that uses a drug‐naïve screen is efficient, but it will necessarily miss those genes with drug‐use effects that are not manifested in predisposing drug‐naïve phenotypes. Nevertheless, through this study, combined with publicly available data, multiple novel candidate genes, high‐throughput testing using multiple drugs and functional annotation of multiple genomic databases, we have identified 22 new drug‐use genes amenable for detailed characterization in viable mutant mice.

The number of genes associated with drug phenotypes from each cohort was different with 15/19 (~80%) of the genes selected in cohort 1 having a drug exposure phenotype and 8/16 (~50%) in cohort 2. With each cohort being selected differently and based on a differing number of behavioral predictors (10 in cohort 1, eight in cohort 2) and finally being tested on a different number of drugs with different assays for alcohol, it is difficult to determine the meaning of this differential hit rate. Of the 19 genes identified to be phenodeviant in cohort 1, using the prioritization of cohort 2, 7 of the 19 were considered phenodeviant under the second cohort's prioritization criteria. Nicotine was only used, with saccharine in cohort 1 and was associated with no KO strains. It is tempting to speculate that the tail suspension test which was included in that first cohort but not in the second cohort was the cause of the higher hit rate although only three of the genes (*Far2*, *Pitx3* and *Pnmt*), had been significant on either of the two TST measures and Pitx3 had no drug‐related associations.

Our hit rate above 50% for drug‐related effects of gene deletion across both cohorts is remarkable given our reliance on detection of phenotypic deviance on 4–5 simple behavioral tasks. It would have been interesting to select a cohort of mice with little or no basic behavioral phenodeviance from the control or that were phenodeviant for a trait not generally thought to be associated with drug use, such as bone composition to see how many such strains have drug preference or consumption phenotypes. This would provide valuable information on whether selecting for the behavioral phenodeviance specifically enriches identification of mutations that impact drug related phenotypes.

It should be noted that throughout the work we never detected a strain effect on BEC in the DID paradigm (it was not measured in the 2 BC). KOs such as *Irf8*, showed a decreased intake, did not display a significantly different BEC which would suggest an altered (slowed) metabolism of ethanol. Similarly male *Tmod2* and *Zbtb4* mice consumed more ethanol in the DID but did not show an elevated BEC, again suggesting that the metabolism of ethanol was altered (increased) in these mutants.

The initial screening identified 15 novel drug‐use gene candidates leveraging data from predisposing drug‐naïve phenotypes, corroborating previous studies that found shared genetic components underlying predisposing drug‐naïve phenotypes and subsequently drug‐use phenotypes.^22^, ^23^ Interestingly, in contrast to findings in the literature,^8^, ^60^, ^61^, ^62^ the relationships we found were not uniform connections between drug‐use phenotypes and their predisposing drug‐naïve phenotypes. Our results indicated more complex and multidimensional relationships that we analyzed further using PCA. In this analysis, strains with significant drug‐use phenotypes were found in all four quadrants of the graph (Figure 3), each representing a different baseline behavioral profile predictive of different drug phenotypes. Two ethanol‐preferring strains that exemplify different baseline behavioral profiles were *Il12rb2* (found among strains with risk‐taking/low‐avoidance behaviors) and *Hspb2* (found among strains with high exploratory/high activity behaviors). Although both KO strains showed an ethanol‐preferring phenotype, the different behavioral profiles segregated along PC2, also correlating with an MA consumption phenotype. The 2 BC choice data reveals that *Il12rb2* KO mice have a significant MA consumption phenotype, whereas *Hspb2* KO mice do not. Thus, results from the PCA revealed the diverse multidimensional nature of the relations underlying the many predisposing behaviors and their predicted drug‐use phenotypes. Rather than reflecting a uniform predictive relationship between each behavioral phenotype and its predisposing effect on drug intake,^8^, ^60^, ^61^, ^62^ these findings indicate a complex interaction of all the predisposing behaviors and their effects on drug‐use phenotypes across different drugs, and that many biological mechanisms support the distinct relations among baseline behaviors and drug‐use phenotypes. They corroborate and extend to psychostimulants, the previous work of Blednov and colleagues which indicated that distinct mutations, albeit on heterogeneous backgrounds, disrupt multiple physiological systems associated with ethanol consumption.^23^ The lack of overlapping pathway membership observed for the detected genes further reveals the tremendous breadth of variation that can result in addiction‐related phenotypes and the potential for sizeable individual variation in mechanisms of addiction vulnerability among those with SUD. Through deeper exploration of these relationships, we can better understand the specific relationships among biological pathways and behavioral processes that lead to heterogeneous behavioral and genetic mechanisms of addiction and substance use.

Much of the historical focus in addiction research has been on studying genetic components underlying drug‐specific effects through alteration of drug‐specific metabolism or drug receptors in the reward pathway.^22^, ^63^, ^64^, ^65^, ^66^, ^67^ These genetic components can play crucial roles in the development of treatments for drug‐specific SUDs. Interestingly, an analysis of functional associations using GeneWeaver and GSEA revealed that the 13 genes have diverse functions and expression patterns with no annotated pathway overlap or any enrichment for similar GO terminology. These results suggest that these genes may each represent independent biological pathways and mechanisms involved in vulnerability to EtOH use and warrant further characterization. For example, *Il12rb2* and *Far2*, the two genes that showed multi‐drug effects (i.e., significant alteration to both EtOH and MA), have diverse biological functions and expression patterns and no enrichment for similar GO terms. *Il12rb2* (interleukin 12 receptor subunit beta 2) is a subunit of the interleukin 12 receptor complex involved in IL12‐dependent signaling and functions in Th1 cell differentiation. It is highly expressed in the pancreas, placenta, skeletal muscle, NK cells and multiple brain regions. In contrast, *Far2* (fatty acyl‐CoA reductase 2) is a member of the short‐chain dehydrogenase/reductase superfamily that functions in fatty acid metabolism. It is highly expressed in intestinal tissue, white blood cells, epididymis and multiple brain regions.

We identified 22 genes not previously connected to drug use, which significantly affected both predisposing drug‐naïve and drug‐self‐administration phenotypes when knocked out. Functional annotation of these genes revealed that the only significant overlap was between *Htr1a* and *Htr7*, which are both part of the canonical pathway for REACTOME_SEROTONIN_RECEPTORS (M6034). Additionally, an extensive literature search revealed direct connections between *Il12rb2* and *Irf8* as part of the cytokine‐mediated pro‐inflammatory immune response^68^ of the central nervous system, with *Irf8* known to induce *Il12* expression.^69^ The few numbers of connections observed between identified genes suggest that, for the most part, all these novel gene candidates potentially represent distinct mechanisms for drug‐use vulnerability.

Although our primary goal was to elucidate gene‐specific effects on predisposing addiction behaviors, we were also interested in the interaction of genotype and sex. Our results corroborate findings from previous studies, which found that sex differences did not have significant effects on MA use^65^, ^67^ but did significantly influence EtOH‐use,^70^, ^71^, ^72^ with female mice consuming higher levels of EtOH than male mice. The only significant strain × sex interaction we observed was in our EtOH DID protocol, where *Tmod2* and *Zbtb4* had significant strain × sex interactions as indicated by decreased consumption for the female strains but no difference in male consumption compared with controls. These results suggest that these genes could be differentially regulated in each sex and their deletion results in more similar drug‐use phenotypes between the sexes. The findings of strain × sex differences in responses of *Tmod2* and *Zbtb4* to EtOH are particularly interesting because these two strains also showed significantly altered MA intake but no effect of sex or strain × sex. Additionally for *Tmod2*, previous studies conducted using strains from the BXD recombinant inbred mice strain panel found sex differences in gene expression in various locations throughout the reward pathway following drug exposure.^21^ Together, these findings suggest that these genes could potentially be regulated in a strain × sex × drug manner.

Addiction is a multi‐phased process, and the genetic mechanisms associated with sustained drug‐use may be independent from that of the transition from initial use to addiction. Further characterization of genes involved in addiction‐related behavior and associated pathways could elucidate their distinct roles in the process of transitioning from recreational use to addiction. Our findings suggest that evaluating single‐gene KO mice using a broad neurobehavioral screen allows the continued identification of novel addiction risk genes. In this project, we detected multiple genes affecting drug‐use phenotypes through diverse biological pathways. Of the many diverse pathways represented by our identified drug‐use genes, we highlighted the potential role of the neuroimmune and cytokine responses in altering drug use which connected three of our novel drug‐use genes with the strongest effects across drugs. Each of these genes would only account for small proportions of the genetic variation and would often be missed using GWAS. The continual screening of KO mice for predisposing drug‐naïve phenotypes can lead to the discovery of previously undetected addiction risk genes across the breadth of pathways involved in these devastating conditions.

## CONFLICT OF INTEREST STATEMENT

Authors have no conflicts of interest to declare.

## Supporting information


**Supplemental Table 1. The JAX KOMP2 Phenotyping Pipeline.** Full details are available in Meehan et al.^9^ and at the International Mouse Phenotyping Consortium website (https://www.mousephenotype.org/) Briefly, mice were weighed weekly from four to seven weeks of age. During week eight mice were tested for exploratory behavior in the open fields and assessed from dysmorphology using the Smithe Kleine Beechman, Harwell, Imperial College, Royal London Hospital phenotype assessment (SHIRPA)^75^ protocol and finally tested for their grip strength. In week nine mice were tested for anxiety‐like behavior using the light–dark box and for exploratory behavior with the holeboard apparatus. In week ten they were tested for acoustic startle by measuring prepulse inhibition. In week eleven they were tested for behavioral response to the tail suspension, rotarod and assessed by EKG. Week 12 brought the glucose tolerance test, followed by a urinalysis to detected Albumin, Creatine, Mg2+ and glucose in week 13. In week 14 the body composition was measured by dual x‐ray absorptiometry, and eye dysmorphology measured by the slit lamp and ophthalmoscope. Sleep was assessed using a home cage sleep monitoring system in week 15 which was followed in week 16 by hearing assessment by auditory brainstem response and vision by electroretinography. Week 17 was used to study seizure susceptibility by measuring the electroconvulsive seizure threshold. Mice were all necropsied at week 18 which included clinical chemistry assays, organ weight, gross pathology, splenic flow cytometry and histological block banking.
**Supplemental Table 2.** A table of each of the behavioral tests and the rationale for each of the collected measures. References are in supplemental references.
**Supplemental Table 3.** A table of the gene perturbations that had been phenotyped by the KOMP phenotyping effort at the time of selecting cohort 1. The list represents at the gene level, which loci were associated with what number of significant behavioral traits and the p‐value of those traits are in the following ten column headers. **Holeboard Genotype P‐value**‐ The total number of nose‐pokes into the sixteen holes in the hole board testing arena during the single twenty‐minute testing session. **Startle Genotype P‐value** Percentage of baseline startle response when lower‐intensity ‘prepulse’ sounds precede a louder ‘pulse’ sound. **TST1 Genotype P‐value** The total amount of time a mouse is immobile while suspended by its tail during the five minute testing session. **TST2 Genotype P‐valu**e The total amount of time a mouse is actively moving while suspended by its tail before it becomes immobile during the five minute testing session. **LD1 Genotype P‐value** The total amount of time, represented as a percentage of total testing time, during which the mouse spent on the light side of the two‐chambered light dark apparatus. **OF1 Genotype P‐value** The total amount of times a mouse rears or jumps during the twenty minute testing session in the open field arena **OF2 Genotype P‐value** Total distance traveled during the twenty minute testing session in the open field arena. **OF3 Genotype P‐ value** The slope of the best fit line measuring the change in distance traveled over the twenty minute test which is broken into four five‐minute time bins **OF4 Genotype P‐value** Total distance traveled during the first 5 minutes of the twenty minute test in the open arena. **OF5 Genotype P‐value** The total amount of time spent in the center 40% of total surface area in the open arena during twenty minute testing.
**Supplemental Table 4.** Table of mice tested in cohort 1 on the three different drugs. Table columns include the official allele name, the gene symbol, the RRID for ordering the mice and the number of males and females of each tested on each drug.
**Supplemental Table 5.** List of all 401 KOMP stains that had been tested at the time of cohort 2 selection. Table includes gene symbol, zygosity tested, if it was phenodeviant on cohort 1, the number of traits it was phenodeviant on in cohort 1, its Mahalanobis Distance Rank in cohort 2, if it was deemed pheondeviant in cohort 2 and if it was tested in cohort 2.
**Supplemental Table 6.** Data for MA preference and consumption in cohort 2. The sample size of each strain and sex tested at each concentration and the mean ± standard error for each measure collected or calculated. Amount of drug consumed, Amount of water consumed, the weight of drug consumes, the preference as a decimal, the total drinking in g/kg and the total water consumed in concentration of ethanol. n.d. represent no data collected.
**Supplemental Table 7.** Results of the DID assays for the mice tested in cohort 2. Table contains official strain nomenclature, Gene Symbol and RRID for strain ordering. The sample size tested and the average plus/minus the standard error of the mean are reported. BEC for each strain are indicated and the F‐statistic and p‐value for strain differences.
**Supplemental Table 8.** Sample sizes of for each of the mutant strains for each of the KOMP phenotyping assays at the time of data analysis.
**Supplemental Table 9.** The table contains the sample size of each strain and sex tested and the mean ± standard error for each concentration of ethanol. n.d. represent no data collected.
**Supplemental Table 10.** The table contains the sample size of each strain and sex tested and the mean ± standard error for each concentration of methamphetamine. n.d. represent no data collected.
**Supplemental Table 11.** The table contains the sample size of each strain and sex tested and the mean ± standard error for each concentration of nicotine. n.d. represent no data collected.
**Supplemental Table 12.** The statistics for each of the three drugs tested. The first four numeric columns represent the p‐value with p < 0.05 is highlight in pink. In the middle is a column relative showing direction of change in the mutant compared with the control using arrow and the final four numeric columns contain the F‐statistics and degrees of freedom for each test. Abbreviations: DrugWeightEthanol = The amount of EtOH consumed (g/kg). DrugWeightMeth = The amount of MA consumed (mg/kg). DrugWeightNicotine = The amount of Nicotine consumed mg/kg. PreferenceEthanol = The preference for EtOH. PreferenceMeth = The preference for MA. PreferenceNicotine = The preference for Nicotine. Total.DrinkingEthanol = The total volume of EtOH and water consumed. Total.DrinkingMeth = The total volume of MA and water consumed. Total.DrinkingNicotine = The total volume of Nicotine and water consumed.
**Supplemental 13** The five resulting dimensions from the PCA analysis of the 22 phenotypic traits. The terms plotted in Figure 3 are in one column and their definitions in another column.
**Supplemental Table 14.** Statistical analysis of MA preference data from cohort 2.
**Supplemental Table 15.** Statistical analysis of MA consumption data from cohort 2.
**Supplemental Table 16.** Statistical results of the DID data with p < 0.05 highlighted in pink. The final column shows the direction of change in relation to the control strain.
**Supplemental Table 17.** Additional supporting evidence for a role of each of the 22 genes identified in these studies in drug‐related pathways or mechanisms.
**Supplemental Table 18.** The GO Biological Process Mappings containing multiple genes within the set of 22 genes with drug related phenotypes. The terms ID, name and genes associated with it are indicated.

## Data Availability

All data sets are available from the Mouse Phenome Database.
