# Supplementary material for: Discovery and validation of genes driving drug‐intake and related behavioral traits in mice
Source: Genes Brain Behav. 2024 Jan 2;23(1):e12875. doi: 10.1111/gbb.12875 (PMC10780947; doi:10.1111/gbb.12875)
Supplement: Supplementary file 1 — Supplemental Table 1. The JAX KOMP2 Phenotyping Pipeline. Full details are available in Meehan et al.9 and at the International Mouse Phenotyping Consortium website (https://www.mousephenotype.org/) Briefly, mice were weighed weekly from four to seven weeks of age. During week eight mice were tested for exploratory behavior in the open fields and assessed from dysmorphology using the Smithe Kleine Beechman, Harwell, Imperial College, Royal London Hospital phenotype assessment (SHIRPA)75 protocol and finally tested for their grip strength. In week nine mice were tested for anxiety‐like behavior using the light–dark box and for exploratory behavior with the holeboard apparatus. In week ten they were tested for acoustic startle by measuring prepulse inhibition. In week eleven they were tested for behavioral response to the tail suspension, rotarod and assessed by EKG. Week 12 brought the glucose tolerance test, followed by a urinalysis to detected Albumin, Creatine, Mg2+ and glucose in week 13. In week 14 the body composition was measured by dual x‐ray absorptiometry, and eye dysmorphology measured by the slit lamp and ophthalmoscope. Sleep was assessed using a home cage sleep monitoring system in week 15 which was followed in week 16 by hearing assessment by auditory brainstem response and vision by electroretinography. Week 17 was used to study seizure susceptibility by measuring the electroconvulsive seizure threshold. Mice were all necropsied at week 18 which included clinical chemistry assays, organ weight, gross pathology, splenic flow cytometry and histological block banking. Supplemental Table 2. A table of each of the behavioral tests and the rationale for each of the collected measures. References are in supplemental references. Supplemental Table 3. A table of the gene perturbations that had been phenotyped by the KOMP phenotyping effort at the time of selecting cohort 1. The list represents at the gene level, which loci were associated with what number of s [file GBB-23-e12875-s001.pdf]

Supplemental Table 1

JAX KOMP2 Pipeline

Age wks				Wt
4-7	Mice (controls and KO) received; Weigh weekly			Wt
8	Open Field	Dysmorphology & SHIRPA	Grip Strength	Wt
9	Light/Dark	Holeboard		Wt
10	Acoustic Startle/ PPI			Wt
11	Tail Suspension	EKG	Rotarod	Wt
12	Glucose Tolerance Test			Wt
13	Urinalysis (Alb, Cre, Mg^{2+}, Glu)			Wt
14	Body Composition X-ray	Eye Dysmorphology Surgery: Slit Lamp, Opthalmoscope		Wt
15	Sleep			Wt
16	Auditory Brainstem Response	Electroretinography		Wt
17	Electroconvulsive Seizure Threshold			Wt
18	Terminal: (8/8) Hematology, Clinical Chemistries, Insulin, Heart Weight<br>Necropsy (2/2) Gross Pathology, Block Banking, Histopathology			Wt

Supplemental Table 2

Test	Phenotype	Rationale
Open Field	**Total Rearing**	The total amount of times a mouse rears or jumps during the twenty minute testing session in the open field arena. This endpoint serves as a measure for anxiety[1,2] which is positively genetically correlated with substance use in mice.[3] Decreased total rearing is an index for increased anxiety.
	**Total Distance Travelled**	Total distance travelled traveled during the twenty minute testing session in the open field arena. Distance traveled serves as a measure of anxiety[1,2] which is positively genetically correlated with substance use in mice.[3] Decreased total distance travelled is an index of increased anxiety.
	**Slope of Distance Travelled**	Slope of the best fit line measuring the change in distance traveled over the twenty minute test which is broken into four five-minute time bins. This measure is calculated to show habituation to the novelty which can be used to measure anxiety or exploratory/risk taking phenotypes.[4] These behaviors are genetically correlated with substance use in mice.[3] Increased slope (increased habituation) is an index for decreased anxiety.
	**Distance First Five Minutes**	Total distance travelled traveled during the first 5 minutes of the twenty minute test in the open arena. Distance traveled in the first five minutes of open field serves as a measure of novelty reactivity and anxiety[5] which has been shown to be predictive of initiation of drug use and progression to compulsive drug use[6] High distance travelled reflects high novelty reactivity and low anxiety.
	**Center Time**	The total amount of time spent in the center 40% of total surface area in the open arena during twenty minute testing. Time spent in the center servers as a measure for anxiety [1,6]and is positively genetically correlated with substance use in mice[3]. High time in the center reflects low anxiety.

**Light/Dark**	**Time in Light**	The total amount of time, represented as a percentage of total testing time, during which the mouse spent on the light side of the two-chambered light dark apparatus during the single twenty minute testing session. Time in light is an index of anxiety [7] and is positively genetically correlated with substance use in mice.[3] Less time in light relative to controls, this indicates HIGH anxiety. More time in light than controls, this reflects excessive risk-taking, a form of impulsivity.
**HoleBoard**	**Holepokes**	The total number of nose-pokes into the sixteen holes in the hole board testing arena during the single twenty-minute testing session. Nose-pokes in a hole board is one of several genetically distinct indexes of novelty seeking[8] which are positive predictors of substance use in mice. High nose-pokes reflect high novelty seeking.
**Acoustic Startle**	**Percent Prepulse Inhibition (%PPI)**	Percentage of baseline startle response when lower-intensity 'prepulse' sounds precede a louder 'pulse' sound. Reduced PPI is a Research Diagnostic Criteria (RDOC) and endophenotype for multiple neuropsychiatric disorders[9] including panic disorder (anxiety) which is positively genetically correlated with substance use in mice [3]. Reduced %PPI is an index for increased anxiety.
**Tail Suspension**	**Time Immobile**	The total amount of time a mouse is immobile while suspended by its tail during the five-minute testing session. Time immobile is recognized as an animal model for efficacy of antidepressants[9] which has been shown to increase chances of drug acquisition and maintenance.[10] While there may be controversy over the validity of this model as a depression-like behavior, many researchers still use time immobile due to its predictive validity.[11,12] A greater time immobile is considered a measure higher levels of depression-like behavior.

	**Latency to Immobility**	The total amount of time a mouse is actively moving while suspended by its tail before it becomes immobile during the five minute testing session. Latency to immobility is recognized as an animal model for the study of depression[9] which has been shown to predict increased drug acquisition and maintenance.[10] Shorter latency to immobility reflects greater depression-like behavior.

Supplemental Table 3

Gene Tested	Zygosity	Number of Phenodeviant Traits	Holeboard Genotype Pvalue	Startle Genotype Pvalue	TST1 Genotype Pvalue	TST2 Genotype Pvalue	LD1 Genotype Pvalue	OF1 Genotype Pvalue	OF2 Genotype Pvalue	OF3 Genotype Pvalue	OF4 Genotype Pvalue	OF5 Genotype Pvalue
Abcg2	HET		NA	NA	0.385	0.297	NA	NA	NA	NA	NA	NA
Adar	HOM	2	0.623	0.915	0.566	0.886	0.141	0.018	0.845	0.113	0.007	0.054
Adora2b	HOM, HET	1	0.282	0.032	0.927	0.566	0.065	0.527	0.149	0.964	0.726	0.795
Adora2b	HOM, HET	1	0.280	0.133	0.234	0.999	0.141	0.470	0.528	0.607	0.609	0.263
Ahrr	HOM	2	0.893	0.512	0.894	0.966	0.174	0.688	0.961	0.338	0.805	0.346
Ahrr	HET	2	0.404	0.496	0.352	0.774	0.489	0.036	0.808	0.174	0.031	0.898
AI464131	HOM		0.986	0.937	0.198	0.170	0.598	NA	NA	NA	NA	NA
Arhgef10	HOM		0.541	0.391	NA	NA	0.397	0.773	0.882	0.260	0.442	0.236
Arid3a	HET	1	0.568	0.629	0.009	0.715	0.288	NA	NA	NA	NA	NA
Arrb2	HOM	5	0.233	0.030	0.058	0.786	0.006	0.034	0.081	0.052	0.043	0.013
Arrb2	HET	5	0.472	0.181	0.298	0.795	0.374	0.020	0.000	0.848	0.331	0.000
Arrdc1	HOM	4	0.780	0.004	0.561	0.741	0.840	0.062	0.202	0.000	0.002	0.018
Arrdc1	HET	4	NA	NA	NA	NA	NA	0.076	0.429	0.025	0.702	0.564
Arsk	HOM	3	0.159	0.031	0.033	0.902	0.059	0.646	0.063	0.447	0.072	0.015
Arsk	HET	3	NA	NA	0.388	0.815	0.930	0.070	0.000	0.596	0.009	0.092
Asb10	HOM		0.714	0.694	0.769	0.540	0.540	0.429	0.788	0.435	0.787	0.058
Asf1a	HET		0.238	0.458	0.379	0.592	0.133	NA	NA	NA	NA	NA
Atr	HET	2	0.863	0.679	0.022	0.480	0.455	0.664	0.005	0.664	0.480	0.814
Atrip	HET	1	0.116	0.837	0.001	0.862	0.218	0.230	0.724	0.185	0.068	0.442
Bach1	HET	4	0.295	0.140	0.436	0.746	0.290	0.014	0.044	0.677	0.040	0.041
Bloc1s2	HET		0.636	0.384	0.915	0.836	NA	NA	NA	NA	NA	NA
Brms1	HET	1	0.022	0.113	0.696	0.857	NA	NA	NA	NA	NA	NA
Btg2	HOM	2	0.059	0.066	0.382	0.708	0.755	0.013	0.464	0.012	0.330	0.725
Bzw2	HOM	6	0.001	0.759	0.003	0.363	0.849	0.000	0.000	0.000	0.064	0.007
C1qa	HOM	1	0.923	0.679	0.786	0.765	0.025	NA	NA	NA	NA	NA
C1qtnf5	HOM	4	0.022	0.030	0.084	0.614	0.998	0.172	0.980	0.010	0.013	0.238
C1qtnf5	HET	4	0.397	0.261	0.005	0.946	0.335	0.027	0.682	0.304	0.078	0.061
C3	HOM		0.382	0.230	0.409	0.674	0.186	0.303	0.971	0.064	0.051	0.735
C9	HOM		0.003	0.007	0.157	0.894	NA	NA	NA	NA	NA	NA
Cacna1s	HET	1	0.045	0.382	0.409	0.664	NA	NA	NA	NA	NA	NA
Cacna2d3	HET		0.181	0.189	0.513	0.930	NA	NA	NA	NA	NA	NA
Car7	HET	1	0.005	0.339	0.143	0.688	NA	NA	NA	NA	NA	NA
Cast	HOM		0.984	0.679	0.104	0.699	NA	NA	NA	NA	NA	NA
Cbln3	HOM		NA	0.216	0.247	0.794	0.924	0.957	0.765	0.014	0.364	0.033
Cbln3	HET		NA	0.260	0.680	0.801	0.303	NA	NA	NA	NA	NA
Ccdc120	HOM	2	0.948	0.383	0.381	0.842	0.312	0.078	0.026	0.351	0.132	0.014
Cd33	HOM	1	0.305	0.675	0.355	0.748	0.005	0.416	0.184	0.208	0.387	0.134
Cdc20	HET		0.029	0.116	0.270	0.386	0.133	NA	NA	NA	NA	NA
Cdk15	HOM		0.699	0.982	0.641	0.473	NA	NA	NA	NA	NA	NA
Ceacam16	HOM	1	0.190	0.312	0.122	0.566	0.538	0.323	0.923	0.020	0.084	0.921

Cenpo	HET	3	0.987	0.934	NA	NA	0.084	0.765	0.000	0.030	0.282	0.000
Cers5	HOM		0.342	0.993	NA	NA	NA	NA	NA	NA	NA	NA
Ces4a	HOM		0.250	0.881	0.877	0.624	NA	NA	NA	NA	NA	NA
Cfb	HOM	2	NA	NA	NA	NA	NA	0.029	0.176	0.350	0.111	0.000
Cgn	HET		0.628	0.165	0.298	0.842	NA	NA	NA	NA	NA	NA
Chn1	HOM	8	0.079	0.012	0.001	0.302	0.009	0.000	0.009	0.000	0.025	0.001
Cinp	HET		0.963	0.286	0.351	0.853	0.025	0.003	0.703	0.003	0.000	0.809
Cited4	HOM		0.198	0.979	0.172	0.527	0.025	0.773	0.010	0.398	0.640	0.339
Cldn13	HOM	3	0.656	0.516	0.365	0.632	NA	NA	NA	NA	NA	NA
Cldn19	HOM	1	0.045	0.637	0.296	0.830	NA	NA	NA	NA	NA	NA
Cml2	HOM		0.204	0.070	0.862	0.691	0.264	0.264	0.568	0.959	0.430	0.587
Col18a1	HOM	3	0.099	0.968	0.556	0.946	0.991	0.000	0.668	0.290	0.000	0.000
Cox7c	HET	1	0.537	0.641	0.826	0.897	0.000	0.078	0.343	0.144	0.084	0.110
Cp	HOM	5	0.660	0.739	NA	NA	0.000	0.215	0.021	0.000	0.001	0.023
Cr1l	HET		0.788	0.324	0.522	0.817	NA	0.949	0.100	0.642	0.669	0.918
Crym	HOM	5	0.001	0.470	0.138	0.753	0.008	0.009	0.796	0.002	0.016	0.235
Cwc27	HET	1	0.013	0.988	NA	NA	NA	0.249	0.803	0.511	0.296	0.160
Cyb5d1	HOM	5	0.625	0.000	0.508	0.495	0.002	0.000	0.156	0.000	0.009	0.087
Cyb5d2	HOM		0.283	0.331	0.179	0.400	NA	NA	NA	NA	NA	NA
Dbn1	HET	1	0.394	0.298	0.226	0.794	0.638	0.010	0.704	0.207	0.107	0.147
Dcps	HET		0.057	0.502	0.382	0.737	NA	NA	NA	NA	NA	NA
Dhx30	HET	2	0.005	0.715	0.758	0.537	0.010	0.348	0.808	0.411	0.485	0.272
Dixdc1	HOM	3	0.217	0.014	0.626	0.952	0.169	0.113	0.932	0.040	0.012	0.186
Dixdc1	HET	3	0.842	0.307	0.299	0.591	0.114	NA	NA	NA	NA	NA
Dmap1	HET		NA	0.152	NA	NA	NA	NA	NA	NA	NA	NA
Dnaja1	HET	3	NA	NA	NA	NA	NA	0.003	0.343	0.023	0.001	0.517
Dnajb12	HET	3	0.847	0.011	0.097	0.742	0.091	0.033	0.875	0.080	0.006	0.814
Dnajb3	HOM	5	0.002	0.995	0.667	0.715	0.007	0.754	0.000	0.049	0.088	0.002
Dnajb7	HOM		0.653	0.892	NA	NA	0.947	0.112	0.086	0.894	0.785	0.196
Dnajc14	HOM	4	0.634	0.992	0.865	0.620	0.509	0.001	0.404	0.014	0.000	0.016
Dnajc16	HET		0.556	0.677	0.909	0.831	0.216	0.386	0.495	0.215	0.517	0.117
Dnajc28	HET	3	0.753	0.355	0.100	0.924	0.355	0.286	0.000	0.072	0.036	0.000
Dnajc28	HET	3	NA	0.130	0.093	0.577	0.503	0.000	0.148	0.053	0.001	0.000
Dnajc5g	HET	2	0.028	0.674	0.119	0.770	0.927	0.246	0.261	0.001	0.126	0.116
Dnajc5g	HET	2	NA	NA	NA	NA	0.209	0.004	0.047	0.002	0.000	0.043
Dnajc9	HET	2	0.077	0.456	0.048	0.914	0.015	0.324	0.410	0.191	0.111	0.053
Dnase1l2	HOM	3	0.044	0.182	0.659	0.462	0.138	0.041	0.582	0.131	0.015	0.701
Dnmt3a	HET	3	0.555	0.031	0.027	0.985	0.135	0.896	0.452	0.024	0.187	0.486
Epb4.1l4a	HOM	1	0.835	0.081	NA	NA	0.000	0.235	0.949	0.231	0.777	0.214
Ermp1	HOM		0.161	0.149	0.113	0.722	NA	NA	NA	NA	NA	NA
Exosc8	HET	4	0.905	0.116	0.034	0.997	0.003	0.757	0.772	0.003	0.038	0.695
Fam161a	HOM	5	0.000	0.858	0.000	0.701	0.809	0.001	0.745	0.012	0.864	0.015
Far2	HOM	6	0.007	0.841	0.002	0.337	0.633	0.039	0.431	0.000	0.000	0.029
Foxo3	HOM	7	0.000	0.013	0.004	0.367	0.430	0.008	0.042	0.388	0.010	0.006
Ghr	HET	4	0.133	0.237	0.010	0.904	0.904	0.021	0.038	0.900	0.074	0.033
Ghrhr	HOM	4	0.272	0.015	0.002	0.733	0.421	0.303	0.961	0.012	0.080	0.000
Ghrhr	HET	4	0.161	0.282	0.020	0.877	0.953	0.001	0.695	0.491	0.003	0.002

Ghsr	HOM		0.123	0.787	0.594	0.858	0.895	0.063	0.543	0.817	0.336	0.244
Gimap6	HOM		0.855	0.901	0.345	0.977	NA	NA	NA	NA	NA	NA
Gipc3	HOM	3	0.378	0.168	0.155	0.259	0.857	0.323	0.007	0.003	0.007	0.896
Glycam1	HOM	5	0.655	0.039	0.034	0.824	0.472	0.000	0.395	0.849	0.009	0.000
Glycam1	HET	5	0.921	0.017	0.127	0.863	0.572	0.395	0.855	0.009	0.025	0.021
Gpnmb	HOM	2	0.002	0.667	0.298	0.857	0.991	0.377	0.109	0.011	0.146	0.492
Gpnmb	HET	2	0.097	0.885	0.090	0.855	0.230	0.026	0.345	0.300	0.915	0.029
H1fx	HOM	3	0.935	0.714	0.747	0.767	0.672	0.015	0.011	0.462	0.034	0.188
H1fx	HET	3	0.010	0.558	0.122	0.813	0.444	0.046	0.335	0.074	0.243	0.316
Hdac10	HOM	3	0.621	0.787	NA	NA	0.000	0.042	0.064	0.397	0.355	0.000
Hsf1	HET	1	0.601	0.849	0.663	0.183	0.243	0.008	0.615	0.635	0.373	0.428
Hsf4	HOM		0.946	0.105	0.236	0.840	0.289	NA	NA	NA	NA	NA
Hspa5	HET	2	0.023	0.234	NA	NA	0.550	0.203	0.017	0.093	0.470	0.088
Hspb1	HOM		0.471	0.472	NA	NA	NA	NA	NA	NA	NA	NA
Hspb2	HOM	3	0.084	NA	0.336	0.284	0.383	0.000	0.327	0.425	0.000	0.000
Hspb3	HOM	4	0.590	0.291	0.691	0.859	0.682	0.113	0.019	0.012	0.002	0.002
Htr1a	HOM	5	0.706	0.059	NA	NA	0.243	0.000	0.010	0.000	0.000	0.008
Htr1b	HET	3	0.558	0.973	0.082	0.773	0.714	0.000	0.018	0.068	0.000	0.110
Htr3b	HOM	2	0.107	0.502	0.726	0.686	0.083	0.074	0.022	0.498	0.319	0.000
Htr3b	HET	2	0.526	0.563	0.119	0.963	0.087	0.142	0.010	0.326	0.832	0.166
Ift88	HET	1	0.105	0.819	0.021	0.477	NA	NA	NA	NA	NA	NA
Igsf11	HOM	4	0.002	0.509	0.757	0.343	0.054	0.000	0.132	0.407	0.001	0.000
Il12rb2	HOM	1	0.644	0.154	NA	NA	0.000	NA	NA	NA	NA	NA
Il24	HOM		0.584	0.948	0.440	0.931	0.363	0.288	0.820	0.068	0.114	0.822
Irf8	HOM	6	0.602	0.034	NA	NA	0.409	0.000	0.005	0.000	0.000	0.000
Itga8	HET		0.132	0.541	0.488	0.551	NA	NA	NA	NA	NA	NA
Jam3	HET	2	0.324	0.012	NA	NA	0.399	0.062	0.013	0.519	0.057	0.272
Kcnh3	HOM	4	0.141	0.208	0.302	0.924	0.813	0.000	0.000	0.044	0.000	0.065
Kcnh3	HET	4	0.696	0.753	0.131	0.902	0.045	0.000	0.000	0.011	0.000	0.596
Kif1b	HET	1	0.016	0.174	0.952	0.149	NA	NA	NA	NA	NA	NA
Kif26b	HET	2	0.636	0.561	0.817	0.562	0.000	0.002	0.739	0.574	0.092	0.107
Krt17	HET		0.645	0.249	0.175	0.101	NA	NA	NA	NA	NA	NA
Lima1	HOM	1	NA	NA	NA	NA	0.040	NA	NA	NA	NA	NA
Loxl1	HOM	1	0.568	0.103	0.550	0.941	0.468	0.755	0.292	0.667	0.305	0.608
Loxl1	HET	1	0.119	0.532	0.049	0.852	0.383	0.675	0.835	0.781	0.734	0.957
Lpar6	HOM	1	0.109	0.595	0.882	0.456	0.009	NA	NA	NA	NA	NA
Lrp1	HET	1	0.017	0.753	0.186	0.866	NA	NA	NA	NA	NA	NA
Lrrc15	HOM		0.697	0.322	NA	NA	NA	NA	NA	NA	NA	NA
Macrod2	HOM	4	NA	0.597	0.117	0.943	0.113	0.000	0.079	0.018	0.001	0.000
Macrod2	HET	4	0.948	0.566	0.470	0.949	0.240	0.001	0.080	0.073	0.480	0.000
Mdk	HET		0.131	0.914	0.087	0.699	NA	NA	NA	NA	NA	NA
Med11	HET		0.297	0.379	0.401	0.990	0.244	0.394	0.572	0.905	0.487	0.034
Mmp8	HOM	2	0.603	0.454	0.979	0.576	0.670	0.034	0.662	0.114	0.034	0.275
Mrps25	HET	2	0.301	0.979	0.038	0.347	0.643	0.194	0.000	0.149	0.199	0.202
Msx1	HET		0.247	0.939	0.178	0.514	0.001	0.003	0.772	0.001	0.000	0.112
Myo7b	HOM		0.518	0.494	NA	NA	NA	NA	NA	NA	NA	NA
Nat1	HOM		0.594	0.262	0.281	0.488	NA	NA	NA	NA	NA	NA

Ncald	HOM	5	0.514	0.322	0.000	0.724	0.257	0.000	0.001	0.000	0.000	0.192
Nefh	HOM		0.433	0.470	0.464	0.793	0.421	0.530	0.064	0.477	0.703	0.914
Nemf	HET	2	0.806	0.304	NA	NA	0.001	0.219	0.312	0.014	0.079	0.166
Nes	HOM		0.499	0.173	0.919	0.739	NA	NA	NA	NA	NA	NA
Nrcam	HOM	2	0.733	0.267	0.953	0.079	0.120	0.000	0.054	0.400	0.006	0.093
Nrcam	HET	2	0.013	0.461	0.817	0.920	0.214	0.150	0.728	0.022	0.987	0.020
Nsf	HET	4	0.089	0.649	0.060	0.968	0.009	0.040	0.247	0.008	0.005	0.196
Nxn	HET		0.199	0.288	0.607	0.847	NA	NA	NA	NA	NA	NA
Ocstamp	HOM	4	0.441	0.129	0.180	0.873	0.713	0.000	0.563	0.003	0.000	0.005
Osm	HOM		0.314	0.401	0.542	0.833	0.086	0.620	0.215	0.880	0.587	0.378
Ovol1	HET	1	0.003	0.931	0.830	0.491	NA	NA	NA	NA	NA	NA
Palb2	HET		NA	0.074	NA	NA	0.331	NA	NA	NA	NA	NA
Parp16	HOM	1	0.565	0.064	0.673	0.198	0.108	0.354	0.001	0.385	0.308	0.714
Parp8	HOM	1	0.829	0.099	0.000	0.862	NA	NA	NA	NA	NA	NA
Pax7	HET		0.844	0.643	0.153	0.946	NA	NA	NA	NA	NA	NA
Pcsk5	HET		0.309	0.577	NA	NA	0.497	0.050	0.035	0.009	0.008	0.130
Pibf1	HET		0.187	0.385	0.994	0.800	NA	NA	NA	NA	NA	NA
Pigh	HET	5	0.104	0.705	0.475	0.640	0.048	0.035	0.327	0.019	0.010	0.015
Pitx3	HOM	3	0.000	0.230	0.000	0.152	0.006	NA	NA	NA	NA	NA
Pnmt	HOM	1	0.014	0.452	NA	NA	NA	NA	NA	NA	NA	NA
Polr2h	HET		0.868	0.338	NA	NA	NA	NA	NA	NA	NA	NA
Ppp1r9b	HOM	7	0.000	0.141	0.000	0.993	0.215	0.000	0.000	0.000	0.009	0.000
Prep	HET	2	0.619	0.339	0.157	0.757	0.000	0.678	0.075	0.057	0.298	0.043
Prkab1	HOM	2	0.884	0.369	0.221	0.779	0.392	0.745	0.034	0.514	0.878	0.020
Prkab1	HET	2	0.625	0.153	0.997	0.925	0.054	0.276	0.007	0.007	0.004	0.000
Prokr1	HOM		0.110	0.962	0.383	0.713	0.967	0.654	0.004	0.183	0.092	0.151
Prokr1	HET		0.954	0.384	0.912	0.931	0.329	0.235	0.524	0.451	0.258	0.073
Prom2	HOM	5	0.024	0.309	0.061	0.995	0.079	0.000	0.518	0.029	0.000	0.003
Prom2	HET	5	0.826	0.773	0.218	0.980	0.629	0.002	0.939	0.214	0.108	0.460
Psen1	HET		0.536	0.142	0.636	0.904	NA	NA	NA	NA	NA	NA
Pycr1	HOM		0.084	0.639	0.194	0.060	NA	NA	NA	NA	NA	NA
Rab27b	HOM	1	0.012	0.825	0.153	0.566	NA	NA	NA	NA	NA	NA
Rab36	HOM		0.515	0.908	0.349	0.632	NA	NA	NA	NA	NA	NA
Rab40c	HET	1	0.015	0.235	NA	NA	NA	NA	NA	NA	NA	NA
Rab43	HOM	1	0.046	0.267	NA	NA	NA	NA	NA	NA	NA	NA
Rab5a	HOM	1	0.009	0.180	NA	NA	NA	NA	NA	NA	NA	NA
Rad21l	HOM	1	NA	NA	NA	NA	0.009	NA	NA	NA	NA	NA
Rasa3	HET	1	0.018	0.270	0.714	0.784	NA	NA	NA	NA	NA	NA
Rdx	HET		0.071	0.786	0.017	0.875	0.527	NA	NA	NA	NA	NA
Resp18	HOM, HET	1	0.228	0.124	NA	NA	0.057	0.767	0.643	0.256	0.770	0.976
Resp18	HOM, HET	1	0.988	0.498	0.577	0.954	0.176	0.422	0.107	0.004	0.053	0.857
Rfc1	HET		0.185	0.646	0.251	0.847	NA	NA	NA	NA	NA	NA
Rgp1	HET	1	0.142	0.140	0.775	0.933	NA	0.197	0.000	0.303	0.105	0.414
Rilpl2	HOM	6	0.483	0.615	0.219	0.702	0.033	0.000	0.005	0.002	0.000	0.003
Rimklb	HOM	5	0.069	0.032	0.909	0.555	NA	0.000	0.001	0.001	0.000	0.614
Rint1	HET	6	0.015	0.699	0.001	0.987	0.045	0.091	0.044	0.000	0.158	0.042
Rnf10	HOM	8	0.010	0.034	0.653	0.710	0.000	0.000	0.000	0.000	0.000	0.000

Rpa1	HET	1	0.524	0.517	0.754	0.876	0.323	0.418	0.031	0.230	0.547	0.435
Sdha	HET		0.226	0.157	0.844	0.559	NA	NA	NA	NA	NA	NA
Setd6	HOM	2	0.017	0.071	0.883	0.819	0.000	NA	NA	NA	NA	NA
Sh3tc2	HOM	3	0.601	0.061	0.823	0.763	0.176	0.259	0.472	0.001	0.045	0.045
Ska2	HET		0.191	0.692	0.497	0.315	NA	NA	NA	NA	NA	NA
Slc25a35	HOM		0.393	0.935	0.931	0.355	NA	NA	NA	NA	NA	NA
Slc6a3	HET	5	0.002	0.332	NA	NA	0.002	0.008	0.119	0.041	0.563	0.004
Sorbs2	HOM	1	0.138	0.016	0.478	0.540	NA	NA	NA	NA	NA	NA
Sox18	HOM		0.329	0.821	0.561	0.673	NA	NA	NA	NA	NA	NA
Sptssa	HET	1	0.488	1.000	0.743	0.777	0.149	0.692	0.003	0.549	0.812	0.371
Sptssb	HOM		0.700	NA	0.469	0.977	NA	NA	NA	NA	NA	NA
Srd5a3	HET		0.739	0.252	0.747	0.785	NA	NA	NA	NA	NA	NA
Stag3	HOM	3	0.289	0.291	NA	NA	0.609	0.090	0.037	0.000	0.000	0.608
Stk16	HET	3	0.937	0.262	0.810	0.778	0.117	0.450	0.034	0.003	0.185	0.000
Stk16	HET	3	0.380	0.433	0.231	0.813	0.626	0.721	0.617	0.023	0.109	0.971
Strn3	HET	6	0.042	0.347	0.947	0.386	0.195	0.000	0.000	0.001	0.000	0.000
Sycp3	HOM		0.053	0.212	0.813	0.609	NA	NA	NA	NA	NA	NA
Thsd1	HOM		0.152	0.411	0.250	0.881	0.199	0.586	0.195	0.399	0.398	0.646
Timp3	HOM	4	0.224	0.730	NA	NA	0.124	0.000	0.152	0.022	0.000	0.000
Tmem136	HOM	3	0.076	0.110	0.921	0.893	0.135	0.278	0.274	0.000	0.002	0.039
Tmem151b	HOM		0.461	0.945	0.236	0.565	NA	NA	NA	NA	NA	NA
Tpgs2	HOM	2	0.000	0.622	0.309	0.468	0.000	0.378	0.059	0.744	0.717	0.832
Tprn	HOM	2	0.104	0.001	0.366	0.290	0.297	0.695	0.001	0.072	0.104	0.848
Trip13	HOM	4	0.502	0.081	0.937	0.969	0.915	0.003	0.507	0.013	0.043	0.000
Trip13	HET	4	0.790	0.675	0.863	0.897	0.409	0.149	0.501	0.575	0.700	0.172
Tspan18	HOM	4	0.030	0.010	0.544	0.816	0.623	0.063	0.009	0.395	0.106	0.000
Ube2c	HET		0.584	0.125	0.193	0.546	NA	NA	NA	NA	NA	NA
Ube2o	HET		0.483	0.836	0.136	0.789	NA	0.475	0.220	0.230	0.610	0.606
Vsig8	HOM		0.955	0.476	NA	NA	NA	NA	NA	NA	NA	NA
Wars2	HET		NA	NA	NA	NA	0.561	0.349	0.221	0.198	0.318	0.690
Wdr12	HET	2	0.068	0.000	0.016	0.937	0.947	NA	NA	NA	NA	NA
Zbtb32	HOM		0.082	0.308	0.302	0.957	NA	NA	NA	NA	NA	NA
Zbtb4	HOM		0.488	0.058	0.804	0.559	NA	NA	NA	NA	NA	NA
Zdhhc11	HOM	4	0.779	0.007	0.233	0.740	NA	0.004	0.084	0.000	0.000	0.096
Zfp346	HET		0.282	0.113	0.792	0.135	NA	NA	NA	NA	NA	NA
Zfp536	HET		0.126	0.254	0.082	0.614	NA	NA	NA	NA	NA	NA
Zmiz2	HET	3	0.000	0.720	NA	NA	0.335	0.545	0.044	0.047	0.111	0.123
Zzef1	HOM		0.259	0.541	NA	NA	NA	0.097	0.142	0.973	0.180	0.395

Supplemental Table 4

			Ethanol			Methamphetamine			Nicotine			
**Gene**	**Knockout Allele**	**RRID**	**Female**	**Male**	**Total**	**Female**	**Male**	**Total**	**Female**	**Male**	**Total**	**Grand Total**
Btg2	**Btg2<tm1b(KOMP)Mbp/2J**	RRID:MMRRC_046828-UCD	**8**	**8**	**16**	**8**	**8**	**16**	**8**	**8**	**16**	**48**
C1qa	**C1qa<tm1b(EUCOMM)Wtsi>/3J**	RRID:MMRRC_046839-UCD	**8**	**7**	**15**	**8**	**8**	**16**	**8**	**8**	**16**	**47**
B6NJ	**C57BL/6NJ**	RRID: IMSR_JAX:005304	**39**	**39**	**78**	**30**	**31**	**61**	**30**	**31**	**61**	**200**
C9	**C9<tm1.1(KOMP)Vlcg>/J**	RRID:MMRRC_046855-UCD	**n.d.**	**n.d.**	**n.d.**	**7**	**7**	**14**	**8**	**8**	**16**	**30**
Cfb	**Cfb<tm1.1(KOMP)Wtsi>/J**	RRID:MMRRC_047078-UCD	**8**	**8**	**16**	**5**	**5**	**10**	**2**	**2**	**4**	**30**
Cp	**Cp<tm1b(KOMP)Wtsi>/J**	RRID:MMRRC_047194-UCD	**8**	**8**	**16**	**11**	**11**	**22**	**5**	**5**	**10**	**48**
Dnajb3	**Dnajb3<tm1.1(KOMP)Vlcg>/J**	RRID:MMRRC_047395-UCD	**8**	**8**	**16**	**5**	**5**	**10**	**7**	**7**	**14**	**40**
Dnase1l2	**Dnase1l2<tm1.1(KOMP)Wtsi>/J**	RRID:MMRRC_047415-UCD	**8**	**8**	**16**	**8**	**8**	**16**	**8**	**7**	**15**	**47**
Epb41l4a	**Epb4.114a<tm1b(KOMP)Mbp>/2J**	RRID:MMRRC_047564-UCD	**8**	**8**	**16**	**8**	**8**	**16**	**8**	**8**	**16**	**48**
Far2	**Far2<tm2b(KOMP)Wtsi>/2J**	RRID:MMRRC_047658-UCD	**8**	**5**	**13**	**8**	**8**	**16**	**8**	**8**	**16**	**45**
Gipc3	**Gipc3<tm1b(KOMP)Wtsi>/J**	RRID:MMRRC_047849-UCD	**8**	**8**	**16**	**8**	**8**	**16**	**8**	**8**	**16**	**48**
Hdac10	**Hdac10<tm1.1(KOMP)Mbp>/J**	RRID:MMRRC_048045-UCD	**8**	**8**	**16**	**7**	**8**	**15**	**8**	**8**	**16**	**47**
Hspb2	**Hspb2<tm1.1(KOMP)Vlcg>/J**	RRID:MMRRC_048111-UCD	**8**	**3**	**11**	**8**	**7**	**15**	**8**	**6**	**14**	**40**
Htr1a	**Htr1a<tm1.1(KOMP)Vlcg>/J**	RRID:MMRRC_048113-UCD	**8**	**8**	**16**	**11**	**8**	**19**	**8**	**8**	**16**	**51**
Il12rb2	**Il12rb2<tm1.1(KOMP)Vlcg>/J**	RRID:MMRRC_048168-UCD	**8**	**8**	**16**	**8**	**8**	**16**	**8**	**8**	**16**	**48**
Lpar6	**Lpar6<tm1.1(KOMP)Vlcg>/J**	RRID:MMRRC_048446-UCD	**3**	**3**	**6**							**6**
Parp8	**Parp8<tm1.1(KOMP)Wtsi>/J**	RRID:MMRRC_048985-UCD	**6**	**6**	**12**	**8**	**8**	**16**	**9**	**7**	**16**	**44**
Pitx3	**Pitx3<tm1.1(KOMP)Vlcg>/J**	RRID:MMRRC_049085-UCD	**2**	**2**	**4**	**5**	**5**	**10**	**8**	**8**	**16**	**30**
Pnmt	**Pnmt<-tm1.1(KOMP)Vlcg>/J**	RRID:MMRRC_049129-UCD	**3**	**3**	**6**							**6**
Rilpl2	**Rilpl2<tm1b(KOMP)Wtsi>/J**	RRID:MMRRC_049454-UCD	**8**	**8**	**16**	**8**	**8**	**16**	**8**	**8**	**16**	**48**
	Grand Total		165	156	321	161	159	320	157	153	310	951

Supplemental Table 5

Gene	Zygosity	Cohort 1 Phenodeviant	Number of Phenodeviant Traits	Cohort 1 Tested	Mahanobus Distance Rank	Cohort 2 Phenodeviant	Cohort 2 Tested
Cp	HOM	Phenodeviant	5	Cohort 1	3	Phenodeviant	Cohort 2
Stk36	HET				2	Phenodeviant	Cohort 2
Dnaja4	HOM				4	Phenodeviant	Cohort 2
Zbtb4	HOM				6	Phenodeviant	Cohort 2
Stx19	HOM				9	Phenodeviant	Cohort 2
Myh10	HET				10	Phenodeviant	Cohort 2
Irf8	HOM	Phenodeviant	6		11	Phenodeviant	Cohort 2
C3	HOM				13	Phenodeviant	Cohort 2
Gpr142	HOM				18	Phenodeviant	Cohort 2
Dnmt3a	HET	Phenodeviant	3		19	Phenodeviant	Cohort 2
Lrrc15	HOM				21	Phenodeviant	Cohort 2
Htr7	HOM				27	Phenodeviant	Cohort 2
Rap2b	HOM				53	Phenodeviant	Cohort 2
Elof1	HET				59	Phenodeviant	Cohort 2
Tmod2	HOM				107	Phenodeviant	Cohort 2
Dnajb3	HOM	Phenodeviant	5	Cohort 1	46	Phenodeviant	
Hspb2	HOM	Phenodeviant	3	Cohort 1	48	Phenodeviant	
Lpar6	HOM	Phenodeviant	1	Cohort 1	61	Phenodeviant	
Il12rb2	HOM	Phenodeviant	1	Cohort 1	68	Phenodeviant	
Pitx3	HOM	Phenodeviant	3	Cohort 1	89	Phenodeviant	
Htr1a	HOM	Phenodeviant	5	Cohort 1	111	Phenodeviant	
Pnmt	HOM	Phenodeviant	1	Cohort 1	165		
Far2	HOM	Phenodeviant	6	Cohort 1	179		
Btg2	HOM	Phenodeviant	2	Cohort 1	189		
Parp8	HOM	Phenodeviant	1	Cohort 1	197		
C9	HOM	Phenodeviant	2	Cohort 1	250		
Gipc3	HOM	Phenodeviant	3	Cohort 1	267		
Rilpl2	HOM	Phenodeviant	6	Cohort 1	308		
C1qa	HOM	Phenodeviant	1	Cohort 1	347		
Epb41l4a	HOM	Phenodeviant	1	Cohort 1	353		
Hdac10	HOM	Phenodeviant	3	Cohort1	258		
Dnase1l2	HOM	Phenodeviant	3	Cohrot 1	339		
Exosc8	HET	Phenodeviant	4		1	Phenodeviant	
Arsk	HOM	Phenodeviant	3		5	Phenodeviant	
Vax1	HET				7	Phenodeviant	
Slc8a3	HOM				8	Phenodeviant	
Rab27b	HOM	Phenodeviant	1		12	Phenodeviant	
Tspan18	HOM	Phenodeviant	4		14	Phenodeviant	
Setd6	HOM	Phenodeviant	2		15	Phenodeviant	
Slc8b1	HOM				16	Phenodeviant	
Jmjd8	HOM				17	Phenodeviant	
Fam186b	HOM				20	Phenodeviant	
Mtf1	HET				22	Phenodeviant	
Mettl16	HET				23	Phenodeviant	
Kif26b	HET	Phenodeviant	2		24	Phenodeviant	
Sdha	HET				25	Phenodeviant	
Myo3b	HOM				26	Phenodeviant	
Cacna2d3	HET				28	Phenodeviant	
Cdc20	HET	Phenodeviant	1		29	Phenodeviant	
Myo7b	HOM				30	Phenodeviant	
Vsig8	HOM				31	Phenodeviant	
Nat1	HOM				32	Phenodeviant	
Rasa3	HET	Phenodeviant	1		33	Phenodeviant	
Zfp689	HOM				34	Phenodeviant	
Ipo11	HET				35	Phenodeviant	

Ttll10	HOM					36	Phenodeviant	
Gnb5	HET					37	Phenodeviant	
Rnf10	HOM	Phenodeviant	8			38	Phenodeviant	
Klk14	HET					39	Phenodeviant	
Rab39	HOM					40	Phenodeviant	
Jam2	HOM					41	Phenodeviant	
Coq6	HET					42	Phenodeviant	
Zdhhc11	HOM	Phenodeviant	4			43	Phenodeviant	
Rab3ip	HOM					44	Phenodeviant	
Gjd4	HOM					45	Phenodeviant	
Rgp1	HET	Phenodeviant	1			47	Phenodeviant	
Prom2	HOM	Phenodeviant	5			49	Phenodeviant	
Sptssa	HET	Phenodeviant	1			50	Phenodeviant	
Mdk	HOM					51	Phenodeviant	
Rab36	HOM					52	Phenodeviant	
Mylip	HOM					54	Phenodeviant	
Asf1a	HET					55	Phenodeviant	
Cyb5d1	HOM	Phenodeviant	5			56	Phenodeviant	
Nemf	HET	Phenodeviant	2			57	Phenodeviant	
Atrip	HET	Phenodeviant	1			58	Phenodeviant	
Ube2m	HET					60	Phenodeviant	
Hyal3	HOM					62	Phenodeviant	
Fastkd5	HOM					63	Phenodeviant	
Nefh	HOM					64	Phenodeviant	
Mrps25	HET	Phenodeviant	2			65	Phenodeviant	
Stx3	HET					66	Phenodeviant	
Snapin	HET					67	Phenodeviant	
Foxo3	HOM	Phenodeviant	7			69	Phenodeviant	
Kif1b	HET	Phenodeviant	1			70	Phenodeviant	
Ahrr	HET		2			71	Phenodeviant	
Ghr	HET	Phenodeviant	4			72	Phenodeviant	
D1Ertd622e	HET					73	Phenodeviant	
Actr8	HET					74	Phenodeviant	
Adora2b	HOM					75	Phenodeviant	
Stx18	HET					76	Phenodeviant	
Cited4	HOM	Phenodeviant	2			77	Phenodeviant	
Cfb	HOM	Phenodeviant	2			78	Phenodeviant	
Mdp1	HOM					79	Phenodeviant	
Hspa5	HET	Phenodeviant	2			80	Phenodeviant	
Agpat1	HET					81	Phenodeviant	
Mrpl51	HET					82	Phenodeviant	
Zmiz2	HET	Phenodeviant	3			83	Phenodeviant	
Ocstamp	HOM	Phenodeviant	4			84	Phenodeviant	
Exoc4	HET					85	Phenodeviant	
Dach1	HET					86	Phenodeviant	
Cdh4	HOM					87	Phenodeviant	
Bbs10	HET					88	Phenodeviant	
Dnajb12	HET	Phenodeviant	3			90	Phenodeviant	
Nmrk2	HOM					91	Phenodeviant	
Nfatc4	HOM					92	Phenodeviant	
Itga8	HET					93	Phenodeviant	
H1fx	HOM	Phenodeviant	3			94	Phenodeviant	
Rab40c	HET	Phenodeviant	1			95	Phenodeviant	
Vcpkmt	HOM					96	Phenodeviant	
Pigh	HET	Phenodeviant	5			97	Phenodeviant	
Ahrr	HOM	Phenodeviant	2			98	Phenodeviant	
Akr1d1	HOM					99	Phenodeviant	
Zfp536	HET					100	Phenodeviant	

Rnf25	HOM				101	Phenodeviant	
Chn1	HOM	Phenodeviant	8		102	Phenodeviant	
Pycr1	HOM				103	Phenodeviant	
Trip13	HET	Phenodeviant	0		104	Phenodeviant	
Ppp1r9b	HOM	Phenodeviant	7		105	Phenodeviant	
Tmem151b	HOM				106	Phenodeviant	
Ncald	HOM	Phenodeviant	5		108	Phenodeviant	
Parp16	HOM	Phenodeviant	1		109	Phenodeviant	
Hc	HOM				110	Phenodeviant	
Cwc27	HOM	Phenodeviant	1		112	Phenodeviant	
Gimap6	HOM				113	Phenodeviant	
Epha10	HOM				114	Phenodeviant	
Dennd2d	HET				115	Phenodeviant	
Orc1	HET				116	Phenodeviant	
Rxfp4	HOM				117	Phenodeviant	
Diaph3	HET				118	Phenodeviant	
Jam3	HET	Phenodeviant	2		119	Phenodeviant	
Postn	HOM				120	Phenodeviant	
Cyb5d2	HOM				121	Phenodeviant	
Dcaf10	HOM				122	Phenodeviant	
Cd33	HOM	Phenodeviant	1		123	Phenodeviant	
Fermt3	HET				124	Phenodeviant	
Bbs4	HET				125	Phenodeviant	
Tppp	HOM				126	Phenodeviant	
Vezt	HET				127	Phenodeviant	
Hfe2	HOM				128	Phenodeviant	
Sycp3	HOM				129	Phenodeviant	
Ap4e1	HOM				130	Phenodeviant	
Zfp42	HET				131	Phenodeviant	
Stx16	HOM				132	Phenodeviant	
Fam217b	HOM				133	Phenodeviant	
Eogt	HOM				134	Phenodeviant	
Cript	HET				135	Phenodeviant	
Wdr12	HET	Phenodeviant	2		136	Phenodeviant	
Dnajc7	HOM				137	Phenodeviant	
Car7	HET	Phenodeviant	1		138	Phenodeviant	
Ghrhr	HOM	Phenodeviant	4		139	Phenodeviant	
Ajap1	HOM				140	Phenodeviant	
Mag	HOM				141	Phenodeviant	
Plk5	HOM				142	Phenodeviant	
Bzw2	HOM	Phenodeviant	6		143	Phenodeviant	
Cacna1s	HET	Phenodeviant	1		144	Phenodeviant	
Nrcam	HET	Phenodeviant	2		145	Phenodeviant	
Ces4a	HOM				146		
Mrgpre	HOM				147		
Rab24	HOM				148		
Sptssb	HOM				149		
Bclaf1	HET				150		
Cldn19	HOM	Phenodeviant	1		151		
Krt9	HOM				152		
Rin3	HOM				153		
Ptms	HET				154		
Sprr3	HOM				155		
Brms1	HET	Phenodeviant	1		156		
Vsig4	HOM				157		
Hsd17b1	HOM				158		
Epb41	HET				159		
Macrod2	HOM	Phenodeviant	4		160		

Lman1l	HOM				161		
Kntc1	HET				162		
Dmap1	HET				163		
Nme6	HET				164		
Dolk	HET				166		
Cast	HOM				167		
Arhgef10	HOM				168		
Ift88	HET	Phenodeviant	1		169		
Prom2	HET	Phenodeviant	5		170		
Trip13	HOM	Phenodeviant	4		171		
Acsm2	HOM				172		
Rimklb	HOM	Phenodeviant	5		173		
Foxi2	HOM				174		
Arrb2	HOM	Phenodeviant	5		175		
Npm3	HOM				176		
Ghsr	HOM				177		
Tpgs2	HOM	Phenodeviant	2		178		
Prpf6	HET				180		
Srd5a3	HET				181		
Sox18	HOM				182		
Klk14	HOM				183		
Arrb1	HOM				184		
Hsf1	HET	Phenodeviant	1		185		
Mpdz	HOM				186		
Spag4	HOM				187		
Igsf11	HOM	Phenodeviant	4		188		
Pkp4	HOM				190		
Ttll6	HOM				191		
Dixdc1	HOM	Phenodeviant	3		192		
Cpb1	HOM				193		
Stag3	HOM	Phenodeviant	3		194		
Dnaja1	HET	Phenodeviant	3		195		
Syn3	HOM				196		
Gpnmb	HOM	Phenodeviant	2		198		
Mdk	HET				199		
Palb2	HET				200		
Hspb1	HOM				201		
Nek11	HOM				202		
Gpnmb	HET	Phenodeviant	2		203		
Krt17	HET				204		
Arid3a	HET	Phenodeviant	1		205		
Il24	HOM				206		
Slc46a1	HET				207		
Adora2b	HET	Phenodeviant	1		208		
Stk16	HOM	Phenodeviant	3		209		
Wee2	HOM				210		
Macrod2	HET	Phenodeviant	4		211		
Kcnh3	HOM	Phenodeviant	4		212		
Adar	HET	Phenodeviant	2		213		
4933427D14Rik	HET				214		
Htr1b	HET	Phenodeviant	3		215		
Akip1	HOM				216		
Espnl	HOM				217		
Lima1	HOM	Phenodeviant	1		218		
Crym	HOM	Phenodeviant	5		219		
Heyl	HOM				220		
Ube2o	HET				221		
Hapln1	HET				222		

Tprn	HOM	Phenodeviant	2		223		
Zwilch	HET				224		
Scg2	HOM				225		
Fam161a	HOM	Phenodeviant	5		226		
Dnajc5g	HOM	Phenodeviant	2		227		
Pibf1	HET				228		
Htr3b	HET	Phenodeviant	2		229		
Vegfb	HOM				230		
Mettl21c	HOM				231		
Fgf9	HET				232		
Dnajb7	HOM				233		
Slc6a3	HET	Phenodeviant	5		234		
Polr2h	HET				235		
Dbn1	HET	Phenodeviant	1		236		
Cdk19	HET				237		
Ermp1	HOM				238		
Clvs1	HOM				239		
Cers5	HOM				240		
C1qtnf5	HOM	Phenodeviant	4		241		
Moxd1	HOM				242		
Mmp8	HOM	Phenodeviant	2		243		
Slc46a3	HOM				244		
Xrcc3	HET				245		
Cenpo	HET	Phenodeviant	3		246		
Iqcj	HOM				247		
Serpinb5	HOM				248		
Rint1	HET	Phenodeviant	6		249		
Slc1a1	HOM				251		
Zfyve26	HOM				252		
Rpa1	HET	Phenodeviant	1		253		
Fxn	HET				254		
Dnajc5g	HET	Phenodeviant	2		255		
Elk1	HOM				256		
Hemgn	HOM				257		
Tmem181a	HOM				259		
Prep	HET	Phenodeviant	2		260		
Rab34	HET				261		
Pacsin2	HOM				262		
Acsf2	HOM				263		
Zbtb32	HOM				264		
Akap11	HOM				265		
Zfp346	HOM				266		
Bach1	HET	Phenodeviant	4		268		
Kcnh3	HET	Phenodeviant	4		269		
Neurl2	HOM				270		
Dnajc9	HET	Phenodeviant	2		271		
Eif4g2	HET				272		
Hsd17b11	HOM				273		
Jup	HET				274		
Mcrs1	HET				275		
Timm22	HET				276		
H1fx	HET	Phenodeviant	3		277		
Slc24a5	HOM				278		
Cdk15	HOM				279		
Loxl1	HOM				280		
Dhx30	HET	Phenodeviant	2		281		
A1cf	HOM				282		
Nat8f2	HOM				283		

Ppp1r35	HET				284		
Glycam1	HET	Phenodeviant	5		285		
Mettl7b	HOM				286		
Adad1	HET				287		
Nsf	HET	Phenodeviant	4		288		
Zfp14	HET				289		
Cox7c	HET	Phenodeviant	1		290		
Pax7	HET				291		
Arsk	HET	Phenodeviant	3		292		
Ifnl3	HOM				293		
Nrn1l	HOM				294		
Rnmtl1	HET				295		
Tbx22	HOM				296		
Prokr1	HOM	Phenodeviant			297		
Il6st	HOM				298		
Zfp219	HOM				299		
Rab43	HOM	Phenodeviant	1		300		
Sorbs2	HOM	Phenodeviant	1		301		
Strn3	HET	Phenodeviant	6		302		
Ddx59	HET				303		
Krt77	HOM				304		
Pcsk4	HOM				305		
Carf	HOM				306		
Bhlhe40	HOM				307		
Cr1l	HET				309		
Rrp8	HET				310		
Chek2	HOM				311		
Cdc34	HET				312		
Glycam1	HOM	Phenodeviant	5		313		
Arf2	HOM				314		
Dcps	HET				315		
Tmem136	HOM	Phenodeviant	3		316		
Pcsk5	HET	Phenodeviant	4		317		
Pkn3	HOM				318		
Arrdc1	HOM	Phenodeviant	4		319		
Rad21l	HOM	Phenodeviant	1		320		
Wars2	HET				321		
Sh3tc2	HOM	Phenodeviant	3		322		
Akr1b8	HOM				323		
Dntt	HOM				324		
Cldn13	HOM				325		
Chtf18	HET				326		
Ska2	HET				327		
Dnajc14	HOM	Phenodeviant	4		328		
Loxl1	HET	Phenodeviant	1		329		
BC100451	HOM				330		
Rab5a	HOM	Phenodeviant	1		331		
Ydjc	HOM				332		
C1qb	HOM				333		
Snx15	HOM				334		
Adck2	HOM				335		
Ccl26	HOM				336		
Rab20	HOM				337		
Zfp961	HOM				338		
Alg2	HET				340		
Cdca5	HET				341		
Atr	HET	Phenodeviant	2		342		
Dixdc1	HET				343		

AI464131	HOM				344			
Ovol1	HET	Phenodeviant	1		345			
Col18a1	HOM	Phenodeviant	3		346			
Ube2c	HET				348			
Abca7	HOM				349			
Ceacam16	HOM	Phenodeviant	1		350			
Nes	HOM				351			
C1qtnf5	HET	Phenodeviant	4		352			
Prkab1	HOM	Phenodeviant	2		354			
Nsun7	HOM				355			
Prkab1	HET	Phenodeviant	2		356			
Pfdn6	HET				357			
Dnajc28	HOM	Phenodeviant	3		358			
Slc25a35	HOM				359			
Timp3	HOM	Phenodeviant	4		360			
Nrcam	HOM	Phenodeviant	2		361			
Arrb2	HET	Phenodeviant	5		362			
Anapc15	HET				363			
Tdrkh	HOM				364			
Htr3b	HOM	Phenodeviant	2		365			
Htr1d	HOM				366			
Smoc2	HOM				367			
Osm	HOM				368			
Hspb3	HOM	Phenodeviant	4		369			
Ghrhr	HET	Phenodeviant	4		370			
Hsf2	HOM				371			
Rrad	HOM				372			
Tbc1d4	HOM				373			
Hsf4	HOM				374			
4921509C19Rik	HOM				375			
Lrp1	HET	Phenodeviant	1		376			
Chp2	HET				377			
Resp18	HET	Phenodeviant	1		378			
Arrdc1	HOM	Phenodeviant	4		379			
Ipp	HOM				380			
Resp18	HOM				381			
Htr1f	HOM				382			
Rfc1	HET				383			
Srp9	HET				384			
Svep1	HET				385			
BC030499	HOM				386			
Dnaaf2	HET				387			
Mfsd10	HOM				388			
Ccdc120	HOM	Phenodeviant	2		389			
Acap1	HOM				390			
Zzef1	HOM				391			
Sgta	HOM				392			
Adad2	HOM				393			
Rhbdl2	HOM				394			
Lipn	HOM				395			
Thsd1	HOM				396			
Epgn	HOM				397			
Asb10	HOM				398			
Fdxacb1	HOM				399			
Stk16	HET	Phenodeviant	3		400			
Cd84	HOM				401			

Supplemental Table 6

Methamphetamine		10 mg/L						20 mg/L					
Female	n=	Amount of Drug Consumed (mL)	Amount of Water Cosnumed (mL)	Drug Weight (g/kg)	Preference (decimal %)	Total Drinking (g/kg bodyweight)	Water Drinking (g/kg body weight)	Amount of Drug Consumed (mL)	Amount of Water Cosnumed (mL)	Drug Weight (g/kg)	Preference (decimal %)	Total Drinking (g/kg bodyweight)	Water Drinking (g/kg body weight)
C57BL/6NJ	19	2.32 ± 0.36	4.05 ± 0.4	0.0021 ± 0.0003	0.37 ± 0.05	294.18 ± 16.64	174.88 ± 17.23	2.11 ± 0.49	4.81 ± 0	0.0037 ± 0.0505	0.31 ± 25.02	312.23 ± 21.38	208.98 ± 1.55
Cp	8	3.48 ± 0.41	3.83 ± 0.59	0.0033 ± 0.0004	0.49 ± 0.06	346.91 ± 21.78	175.58 ± 27.23	1.71 ± 0.46	5.31 ± 0	0.0032 ± 0.0329	0.25 ± 16.02	329.97 ± 17.22	240.47 ± 1.05
Dnaja4	8	3.15 ± 0.61	5.53 ± 0.46	0.0028 ± 0.0005	0.35 ± 0.06	385.61 ± 25.85	234.74 ± 22.69	2.65 ± 0.71	3.54 ± 0	0.0046 ± 0.0649	0.45 ± 30.94	269.32 ± 27.18	145 ± 1.4
Elof1	8	3.07 ± 0.4	3.36 ± 0.43	0.0027 ± 0.0004	0.48 ± 0.07	288.14 ± 15.49	138.59 ± 19.62	1.8 ± 0.67	3.82 ± 0	0.0032 ± 0.0761	0.34 ± 28.36	252.62 ± 30.89	158.13 ± 1.74
Gpr142	8	3.61 ± 1.06	3.51 ± 0.74	0.0034 ± 0.0011	0.49 ± 0.12	327.35 ± 37.23	149.49 ± 27.67	1.06 ± 2.51	5.9 ± 0	0.0046 ± 0.0698	0.29 ± 57.9	388.9 ± 44.76	259.23 ± 1.7
Lrrc15	8	4.07 ± 0.49	4.86 ± 0.26	0.0039 ± 0.0005	0.45 ± 0.03	425.01 ± 26.16	213.87 ± 9.41	1.92 ± 0.37	5.41 ± 0	0.0036 ± 0.034	0.26 ± 13.81	348.17 ± 15.05	238.65 ± 1.49
Rap2b	8	3.89 ± 0.56	4.11 ± 0.64	0.0034 ± 0.0005	0.49 ± 0.05	350.56 ± 47.54	174.63 ± 31.45	2.61 ± 0.82	4.34 ± 0	0.0047 ± 0.076	0.41 ± 33.59	299.98 ± 31.19	175.98 ± 2.49
Stx19	8	2.99 ± 0.3	5.31 ± 0.65	0.0027 ± 0.0003	0.38 ± 0.05	378.61 ± 22.51	229.63 ± 26.95	2.15 ± 0.53	4.84 ± 0	0.0039 ± 0.0544	0.31 ± 26.01	319.81 ± 23.44	211.41 ± 1.1
Zbtb4	8	1.84 ± 0.4	3.77 ± 0.4	0.0018 ± 0.0004	0.32 ± 0.06	274.16 ± 16.95	173.85 ± 18.33	1.9 ± 0.41	4.58 ± 0	0.0037 ± 0.0406	0.3 ± 14.54	314.95 ± 18.87	211.78 ± 1.28
C3	7	3.48 ± 0.61	2.62 ± 0.44	0.0029 ± 0.0006	0.48 ± 0.1	265.52 ± 43.34	123.97 ± 24.55	1.87 ± 0.83	4.85 ± 0	0.0036 ± 0.061	0.31 ± 41.53	326.19 ± 40.68	222.45 ± 1.43
Tmod2	8	2.55 ± 0.53	2.9 ± 0.61	0.0026 ± 0.0006	0.46 ± 0.06	279.35 ± 38.93	141.3 ± 28.91	1.93 ± 0.86	4.43 ± 0	0.004 ± 0.056	0.33 ± 50.32	333.09 ± 47.04	223.29 ± 1.96
Myh10	8	2.43 ± 0.42	4.11 ± 0.79	0.0021 ± 0.0004	0.4 ± 0.08	287.37 ± 29.47	171 ± 35.58	4.06 ± 0.43	2.74 ± 0	0.0072 ± 0.0538	0.59 ± 26.75	298.08 ± 16.24	112.24 ± 1.72
Stk36	8	3.76 ± 0.41	5.13 ± 0.54	0.0032 ± 0.0003	0.43 ± 0.03	385.46 ± 28.17	207.63 ± 21.64	2.31 ± 0.67	4.31 ± 0	0.004 ± 0.0792	0.37 ± 21.49	289.22 ± 27.23	174.52 ± 1.08
Dnmt3a	9	3.13 ± 0.42	3.49 ± 0.54	0.0024 ± 0.0003	0.48 ± 0.07	248.54 ± 10.83	120.45 ± 18.23	1.8 ± 0.33	4.82 ± 0	0.0027 ± 0.0285	0.27 ± 12.37	247.27 ± 10.59	166.94 ± 0.98
Htr7	8	4.57 ± 0.35	2.85 ± 0.37	0.0045 ± 0.0004	0.62 ± 0.04	365.41 ± 24.35	133.57 ± 19.69	2.59 ± 0.43	6.07 ± 0	0.0051 ± 0.061	0.27 ± 45.29	385.44 ± 37.36	242.32 ± 1.86
Irf8	8	4.54 ± 0.93	3.72 ± 0.8	0.0045 ± 0.001	0.54 ± 0.09	409.23 ± 41.33	173.57 ± 36.44	2.52 ± 0.76	3.98 ± 0	0.0049 ± 0.0762	0.42 ± 40.36	321.28 ± 36.1	185.53 ± 1.67

Methamphetamine		10 mg/L						20 mg/L					
Male	n=	Amount of Drug Consumed (mL)	Amount of Water Cosnumed (mL)	Drug Weight (g/kg)	Preference (decimal %)	Total Drinking (g/kg bodyweight)	Water Drinking (g/kg body weight)	Amount of Drug Consumed (mL)	Amount of Water Cosnumed (mL)	Drug Weight (g/kg)	Preference (decimal %)	Total Drinking (g/kg bodyweight)	Water Drinking (g/kg body weight)
C57BL/6NJ	20	2.73 ± 0.49	3.04 ± 0.6	0.0023 ± 0.0004	0.49 ± 0.07	235.15 ± 22.48	114.75 ± 19.65	2.76 ± 0.66	2.89 ± 0.34	0.0045 ± 0.0011	0.45 ± 0.09	232.1 ± 17.48	112.37 ± 14.25
Cp	8	2.73 ± 0.49	3.04 ± 0.6	0.0023 ± 0.0004	0.49 ± 0.07	235.15 ± 22.48	114.75 ± 19.65	2.76 ± 0.66	2.89 ± 0.34	0.0045 ± 0.0011	0.45 ± 0.09	232.1 ± 17.48	112.37 ± 14.25
Dnaja4	4	2.32 ± 0.35	3.85 ± 0.73	0.0017 ± 0.0002	0.39 ± 0.09	236.67 ± 23.14	142.69 ± 29.15	3.26 ± 0.16	3.22 ± 0.67	0.005 ± 0.0003	0.52 ± 0.05	245.86 ± 21.75	115.71 ± 21.09
Elof1	8	2.12 ± 0.39	3.75 ± 0.64	0.0016 ± 0.0003	0.38 ± 0.06	221.94 ± 23.13	132.77 ± 21.23	2.35 ± 0.57	3.62 ± 0.81	0.0035 ± 0.0008	0.41 ± 0.09	225.17 ± 26.62	128.03 ± 28.72
Gpr142	8	2.77 ± 0.79	3.31 ± 0.47	0.002 ± 0.0006	0.45 ± 0.11	216.6 ± 19.39	110.28 ± 13.97	2.93 ± 0.76	3.6 ± 0.91	0.0041 ± 0.001	0.45 ± 0.11	233.25 ± 33.95	121.64 ± 31.95
Lrrc15	8	2.87 ± 0.38	4.46 ± 0.66	0.0023 ± 0.0003	0.4 ± 0.04	286.21 ± 29.33	162.52 ± 22.05	2.33 ± 0.47	4.34 ± 0.43	0.0037 ± 0.0007	0.34 ± 0.06	265.37 ± 17.49	161.45 ± 17.25
Rap2b	8	3.9 ± 0.57	3.64 ± 0.59	0.0025 ± 0.0005	0.47 ± 0.08	235.18 ± 45.33	129.43 ± 21.52	3.26 ± 0.47	3.21 ± 0.51	0.0049 ± 0.0007	0.51 ± 0.04	241.48 ± 33.42	113.87 ± 19.11
Stx19	8	3.19 ± 0.57	4.09 ± 0.68	0.0021 ± 0.0003	0.45 ± 0.07	242.73 ± 23.35	128.72 ± 22.63	2.65 ± 0.42	3.58 ± 0.57	0.0036 ± 0.0006	0.44 ± 0.07	210.63 ± 19.81	111.71 ± 19.4
Zbtb4	8	2.96 ± 0.51	2.83 ± 0.89	0.0023 ± 0.0004	0.56 ± 0.1	229.66 ± 35.28	105.51 ± 32.28	2.39 ± 0.39	4.66 ± 0.49	0.0038 ± 0.0006	0.34 ± 0.04	282.1 ± 21.92	179.98 ± 22.02
C3	8	3.34 ± 0.75	5.12 ± 0.6	0.0024 ± 0.0005	0.37 ± 0.06	306.91 ± 31.08	176.36 ± 20.22	2.05 ± 0.34	4.38 ± 0.8	0.003 ± 0.0005	0.38 ± 0.09	234.68 ± 30.9	151.06 ± 27.6
Tmod2	8	2.45 ± 0.42	4.19 ± 0.65	0.0021 ± 0.0003	0.39 ± 0.08	297.89 ± 22.22	180.43 ± 29.67	1.72 ± 0.28	2.42 ± 0.49	0.0031 ± 0.0005	0.44 ± 0.05	183.97 ± 26.8	102.03 ± 19.81
Myh10	8	3.65 ± 0.61	5.05 ± 0.68	0.0025 ± 0.0004	0.41 ± 0.05	301.01 ± 29.99	166.49 ± 21.18	2.94 ± 0.79	4.55 ± 0.6	0.0041 ± 0.001	0.37 ± 0.08	262.52 ± 24.34	152.63 ± 22.37
Stk36	8	2.65 ± 0.4	4.14 ± 0.34	0.0019 ± 0.0003	0.39 ± 0.05	251.85 ± 11.47	146.85 ± 13	2.57 ± 0.64	3.83 ± 0.65	0.0038 ± 0.0009	0.4 ± 0.09	237.24 ± 14.03	135.85 ± 23.29
Dnmt3a	8	3.48 ± 0.5	3.71 ± 0.72	0.0025 ± 0.0004	0.5 ± 0.06	253.25 ± 23.23	119.66 ± 23.06	2.42 ± 0.48	5.42 ± 0.32	0.0034 ± 0.0007	0.3 ± 0.05	276.78 ± 9.75	176.4 ± 8.89
Htr7	8	3.51 ± 0.63	3.48 ± 0.69	0.0025 ± 0.0005	0.51 ± 0.07	249.79 ± 29.86	117.48 ± 22.35	2.4 ± 0.46	4.63 ± 0.82	0.0034 ± 0.0007	0.37 ± 0.08	252.21 ± 30.85	159.03 ± 29.15
Irf8	8	3.99 ± 0.6	3.21 ± 0.48	0.0031 ± 0.0005	0.55 ± 0.07	282.03 ± 15.81	121.31 ± 20.56	2.61 ± 0.66	3.7 ± 0.7	0.0041 ± 0.001	0.41 ± 0.08	246.01 ± 25.6	136.35 ± 24.98

40 mg/L						80 mg/L					
Amount of Drug Consumed (mL)	Amount of Water Cosnumed (mL)	Drug Weight (g/kg)	Preference (decimal %)	Total Drinking (g/kg bodyweight)	Water Drinking (g/kg body weight)	Amount of Drug Consumed (mL)	Amount of Water Cosnumed (mL)	Drug Weight (g/kg)	Preference (decimal %)	Total Drinking (g/kg bodyweight)	Water Drinking (g/kg body weight)
1.55 ± 0.25	5.41 ± 0.49	0.0058 ± 0.0009	0.25 ± 0.05	323.37 ± 16.56	235.48 ± 21.38	1.23 ± 0.14	6.29 ± 0.33	0.0092 ± 0.0012	0.17 ± 0.02	348.55 ± 13.2	273.18 ± 14.73
1.05 ± 0.13	6.61 ± 0.32	0.0039 ± 0.0005	0.14 ± 0.02	361.94 ± 16.58	302.36 ± 15.87	1.7 ± 0.47	5.67 ± 0.83	0.0128 ± 0.0034	0.26 ± 0.1	349.91 ± 25.41	259.88 ± 38.25
1.4 ± 0.18	6.41 ± 0.36	0.005 ± 0.0007	0.18 ± 0.03	342.98 ± 6.51	266.31 ± 8.45	1.05 ± 0.14	6.19 ± 0.77	0.0074 ± 0.001	0.16 ± 0.03	321.14 ± 32.31	260.93 ± 32.6
1.74 ± 0.29	5.5 ± 0.49	0.0063 ± 0.0011	0.25 ± 0.05	322.78 ± 16.51	222.35 ± 18.66	1.33 ± 0.09	6.63 ± 0.38	0.0096 ± 0.0007	0.17 ± 0.01	354.8 ± 17.5	268.48 ± 13.98
0.64 ± 4.94	4.94 ± 0.91	0.0064 ± 0.002	0.28 ± 0.1	299.8 ± 29.49	206.96 ± 33.77	1.57 ± 0.33	6.24 ± 0.75	0.0116 ± 0.0023	0.21 ± 0.05	357.35 ± 38.41	269.52 ± 38.03
1.49 ± 0.29	6.18 ± 0.85	0.0058 ± 0.0013	0.23 ± 0.08	360.08 ± 25.27	269.04 ± 35.22	1.02 ± 0.22	6.65 ± 0.32	0.0076 ± 0.0016	0.13 ± 0.03	364.28 ± 12.57	293.74 ± 14.63
2.49 ± 0.55	4.89 ± 0.64	0.0089 ± 0.002	0.33 ± 0.07	326.6 ± 33.73	207.22 ± 30.57	2.39 ± 0.66	4.15 ± 0.73	0.0168 ± 0.0051	0.37 ± 0.05	283.4 ± 53.22	169.09 ± 28.8
1.1 ± 0.08	6.37 ± 0.59	0.004 ± 0.0003	0.15 ± 0.02	341.2 ± 28.06	276.83 ± 25.71	1.19 ± 0.19	6.91 ± 0.17	0.0087 ± 0.0013	0.14 ± 0.02	370.37 ± 12.9	300.92 ± 7.45
1.28 ± 0.17	4.33 ± 0.55	0.005 ± 0.0006	0.26 ± 0.06	275.46 ± 24.39	201.39 ± 26.48	1.33 ± 0.19	5.25 ± 0.33	0.0104 ± 0.0014	0.21 ± 0.03	321.19 ± 15.94	243.06 ± 16.49
1.43 ± 0.12	6.13 ± 0.55	0.0055 ± 0.0005	0.2 ± 0.02	364.94 ± 29.97	283.22 ± 31.79	1.41 ± 0.19	5.01 ± 0.83	0.0109 ± 0.0017	0.26 ± 0.05	302.77 ± 35.91	219.53 ± 32.39
1.96 ± 0.24	4.06 ± 0.69	0.0082 ± 0.0011	0.36 ± 0.07	312.39 ± 28.7	200.66 ± 33.94	1.76 ± 0.23	5.34 ± 0.58	0.0149 ± 0.0021	0.26 ± 0.05	369.1 ± 23.7	263.25 ± 25.7
1.72 ± 0.25	5.54 ± 0.47	0.0059 ± 0.0007	0.24 ± 0.04	321.35 ± 23.8	236.99 ± 27.42	0.99 ± 0.1	6.33 ± 0.23	0.007 ± 0.0009	0.14 ± 0.01	324.22 ± 21.03	266.75 ± 18.09
1.08 ± 0.06	6.88 ± 0.55	0.0038 ± 0.0002	0.14 ± 0.01	347.68 ± 24.15	279.55 ± 23.04	1.11 ± 0.12	7.42 ± 0.44	0.0078 ± 0.0009	0.13 ± 0.01	373.34 ± 22.9	301.42 ± 19.07
0.98 ± 0.1	5.28 ± 0.32	0.003 ± 0.0003	0.16 ± 0.01	235.94 ± 16.1	184.04 ± 12.19	1.36 ± 0.09	6.01 ± 0.47	0.0082 ± 0.0007	0.19 ± 0.02	278 ± 18.49	210.73 ± 18.85
1.86 ± 0.24	5.84 ± 0.33	0.0074 ± 0.001	0.24 ± 0.03	377.12 ± 15.3	268.35 ± 11.59	1.4 ± 0.13	6.95 ± 0.21	0.011 ± 0.0011	0.17 ± 0.01	409.15 ± 15.91	321.84 ± 15.12
1.67 ± 0.26	5.5 ± 0.64	0.0066 ± 0.001	0.25 ± 0.06	353.99 ± 22.68	261.61 ± 34.58	1.59 ± 0.35	6.26 ± 0.77	0.0125 ± 0.0027	0.23 ± 0.06	386.92 ± 44.43	290.16 ± 35.26

40 mg/L						80 mg/L					
Amount of Drug Consumed (mL)	Amount of Water Cosnumed (mL)	Drug Weight (g/kg)	Preference (decimal %)	Total Drinking (g/kg bodyweight)	Water Drinking (g/kg body weight)	Amount of Drug Consumed (mL)	Amount of Water Cosnumed (mL)	Drug Weight (g/kg)	Preference (decimal %)	Total Drinking (g/kg bodyweight)	Water Drinking (g/kg body weight)
1.19 ± 0.21	5.25 ± 0.33	0.004 ± 0.0007	0.18 ± 0.03	264.25 ± 14.3	201.51 ± 9.19	1.48 ± 0.22	5.65 ± 0.35	0.0096 ± 0.0013	0.21 ± 0.03	295.31 ± 20.76	221.53 ± 21.43
1.19 ± 0.21	5.25 ± 0.33	0.004 ± 0.0007	0.18 ± 0.03	264.25 ± 14.3	201.51 ± 9.19	1.48 ± 0.22	5.65 ± 0.35	0.0096 ± 0.0013	0.21 ± 0.03	295.31 ± 20.76	221.53 ± 21.43
2.56 ± 0.75	4.59 ± 1.13	0.0076 ± 0.002	0.38 ± 0.13	275.02 ± 25.11	171.85 ± 46.6	1.98 ± 0.46	4.84 ± 0.59	0.0092 ± 0.0039	0.23 ± 0.1	184.23 ± 61.41	175.81 ± 19.86
1.58 ± 0.27	5.65 ± 0.54	0.0049 ± 0.0009	0.22 ± 0.03	277.3 ± 27.1	201.39 ± 20.03	1.47 ± 0.24	5.32 ± 0.63	0.0091 ± 0.0016	0.24 ± 0.06	257.9 ± 15.26	188.27 ± 21.42
1.75 ± 0.41	4.07 ± 0.74	0.005 ± 0.0012	0.33 ± 0.1	207.41 ± 16.34	136.97 ± 26.39	1.46 ± 0.32	4.79 ± 0.65	0.0085 ± 0.0021	0.26 ± 0.08	222.31 ± 15.65	159.86 ± 21.66
1.69 ± 0.38	5.23 ± 0.56	0.0053 ± 0.0011	0.25 ± 0.06	276.21 ± 22.16	194.37 ± 22.19	1.3 ± 0.25	5.93 ± 0.64	0.0083 ± 0.0017	0.19 ± 0.04	288.64 ± 28.02	218.8 ± 24.16
2.05 ± 0.34	4.39 ± 0.64	0.0062 ± 0.0012	0.34 ± 0.07	238.42 ± 14.78	152.1 ± 21.05	2.12 ± 0.32	6.04 ± 0.67	0.0112 ± 0.0024	0.24 ± 0.06	261.58 ± 42.97	210.83 ± 21.71
1.53 ± 0.26	4.85 ± 0.45	0.0042 ± 0.0008	0.24 ± 0.04	216.82 ± 21.88	150.96 ± 15.66	1.71 ± 0.38	5.88 ± 0.45	0.0092 ± 0.0021	0.22 ± 0.04	252.11 ± 15.25	180.43 ± 10.12
2.29 ± 0.33	3.29 ± 0.45	0.0073 ± 0.001	0.42 ± 0.07	223.78 ± 16.94	127.37 ± 18.65	1.88 ± 0.16	4.41 ± 0.46	0.012 ± 0.001	0.31 ± 0.03	250.78 ± 17.64	168.12 ± 16.29
2.06 ± 0.45	4.85 ± 0.72	0.006 ± 0.0013	0.32 ± 0.09	251.31 ± 12.79	165.88 ± 23.51	1.39 ± 0.18	6.13 ± 0.5	0.008 ± 0.001	0.19 ± 0.03	273.05 ± 16.05	211.58 ± 17.25
1.84 ± 0.34	2.35 ± 0.56	0.0067 ± 0.0013	0.47 ± 0.08	185.45 ± 22.03	98.26 ± 21.77	1.64 ± 0.29	5.21 ± 0.76	0.0114 ± 0.002	0.27 ± 0.07	303.98 ± 22.81	221.94 ± 31.75
1.88 ± 0.32	5.66 ± 0.38	0.0052 ± 0.0008	0.24 ± 0.04	263.57 ± 17.65	187.9 ± 12.98	1.94 ± 0.32	4.72 ± 0.63	0.0097 ± 0.0022	0.23 ± 0.05	221.14 ± 37.61	157.1 ± 20.91
1.53 ± 0.31	4.91 ± 0.62	0.0045 ± 0.0009	0.26 ± 0.06	241.05 ± 24.36	175.09 ± 23.56	1.26 ± 0.17	5.66 ± 0.73	0.0075 ± 0.0011	0.22 ± 0.05	258.34 ± 28.65	200.28 ± 25.43
1.23 ± 0.18	5.94 ± 0.36	0.0035 ± 0.0005	0.18 ± 0.03	254.96 ± 14.52	195.76 ± 15.01	1.26 ± 0.13	6.16 ± 0.31	0.007 ± 0.0006	0.17 ± 0.02	262.02 ± 12.56	201.95 ± 11.58
1.96 ± 0.41	5.64 ± 0.81	0.0049 ± 0.0012	0.25 ± 0.09	236.1 ± 38.23	191.25 ± 26.7	1.68 ± 0.28	5.75 ± 0.69	0.0095 ± 0.0015	0.24 ± 0.03	263.87 ± 27.64	195.26 ± 22.54
2.19 ± 0.52	4.9 ± 0.68	0.0069 ± 0.0016	0.32 ± 0.08	277.26 ± 18.28	182.26 ± 24.7	1.21 ± 0.12	5.93 ± 0.54	0.0077 ± 0.0008	0.18 ± 0.03	279.14 ± 18.62	220.59 ± 18.7

Supplemental Table 7

Female							
**Gene**	**Allele**	**RRID**	n=	Day 1	Day 2	Day 3	Day 4
C57BL/6NJ	C57BL/6NJ	RRID: IMSR_JAX:005304	18	3.48 ± 0.29	4.5 ± 0.32	4.84 ± 0.33	9.03 ± 0.44
Cp	$Cp^{tm1b(KOMP)Wtsi}/J^{(-/-)}$	RRID:MMRRC_047194-U(	8	3.43 ± 0.4	4.47 ± 0.29	4.76 ± 0.28	8.28 ± 0.48
Dnaja4	$Dnaja4^{tm1b(KOMP)Wtsi}/J^{(-/-)}$	RRID:MGI:5522543	8	3.04 ± 1.09	5.58 ± 1.4	6.21 ± 1.22	10.27 ± 1.96
Elof1	$Elof1^{tm1.1(KOMP)Vlcg/J(+/-)}$	RRID:IMSR_JAX:025191	8	2.18 ± 0.41	3.53 ± 0.51	3.74 ± 0.49	8.11 ± 0.46
Lrrc15	$Lrrc15^{tm1b(KOMP)Wtsi}/J^{(-/-)}$	RRID:IMSR_JAX:023021	8	3.25 ± 0.52	4.64 ± 0.77	4.81 ± 0.58	9.96 ± 0.82
Rap2b	$Rap2b^{tm1.1(KOMP)Vlcg}/J^{(+/-)}$	RRID:IMSR_JAX:025590	8	2.53 ± 0.35	4.09 ± 0.3	4.67 ± 0.32	9.17 ± 1.15
Stx19	$Stx19^{tm1.1(KOMP)Vlcg}/2J^{(-/-)}$	RRID:MGI:5603919	8	3.02 ± 0.51	5.01 ± 0.69	5.06 ± 0.97	9.44 ± 1.02
Zbtb4	$Zbtb4^{tm1.1(KOMP)Vlcg}/J^{(-/-)}$	RRID:IMSR_JAX:023660	8	2.54 ± 0.34	3.96 ± 0.28	3.74 ± 0.35	6.38 ± 0.64
C3	$C3^{tm1.1(KOMP)Vlcg}/J^{(-/-)}$	RRID:IMSR_JAX:022098	7	3.02 ± 0.57	5 ± 0.45	4.31 ± 0.38	8.57 ± 1.04
Tmod2	$Tmod2^{tm1b(KOMP)Wtsi}/2J^{(-/-)}$	RRID:IMSR_JAX:024939	8	2.01 ± 0.61	2.92 ± 0.42	4.2 ± 0.41	7.3 ± 0.88
Myh10	$Myh10^{em1(IMPC)J}/J^{(+/-)}$	RRID:MGI:5884012	8	3.85 ± 0.55	4.31 ± 0.52	4.39 ± 0.42	7.83 ± 0.7
Stk36	$Stk36^{em1(IMPC)J}/J^{(-/-)}$	RRID:MGI:5884030	8	3.47 ± 0.5	4.94 ± 0.77	4.75 ± 0.66	8.34 ± 0.6
Dnmt3a	$Dnmt3a^{tm1b(KOMP)Wtsi}/J^{(+/-}$)	RRID:IMSR_JAX:018838	5	3.26 ± 0.46	4.51 ± 0.96	4.5 ± 1.06	7.74 ± 1.55
Gpr142	$Gpr142^{tm1.1(KOMP)Vlcg}/J^{(-/-)}$	RRID:IMSR_JAX:026065	8	3.35 ± 0.77	3.88 ± 0.66	4.61 ± 0.68	7.45 ± 0.62
Htr7	$Htr7^{tm1b(KOMP)Wtsi}/J^{(-/-)}$	RRID:IMSR_JAX:023282	8	3.27 ± 0.31	4.2 ± 0.45	4.27 ± 0.73	9.03 ± 0.6
Irf8	$Irf8^{tm1b(KOMP)Wtsi}/J^{(-/-)}$	RRID:IMSR_JAX:018655	8	3.03 ± 0.35	3.57 ± 0.54	2.75 ± 0.33	5.4 ± 0.71
**Male**							
C57BL/6NJ	C57BL/6NJ	RRID: IMSR_JAX:005304	18	1.61 ± 0.2	2.01 ± 0.24	1.97 ± 0.24	3.73 ± 0.45
Cp	$Cp^{tm1b(KOMP)Wtsi}/J^{(-/-)}$	RRID:MMRRC_047194-U(	8	1.32 ± 0.31	1.75 ± 0.52	2.36 ± 0.53	3.69 ± 0.62
Dnaja4	$Dnaja4^{tm1b(KOMP)Wtsi}/J^{(-/-)}$	RRID:MGI:5522543	8	2.85 ± 0.5	3.93 ± 0.3	3.77 ± 0.34	5.14 ± 1.02
Elof1	$Elof1^{tm1.1(KOMP)Vlcg/J(+/-)}$	RRID:IMSR_JAX:025191	8	1.34 ± 0.28	1.27 ± 0.3	1.72 ± 0.27	3.24 ± 0.47
Lrrc15	$Lrrc15^{tm1b(KOMP)Wtsi}/J^{(-/-)}$	RRID:IMSR_JAX:023021	8	2.25 ± 0.49	2.52 ± 0.22	2.5 ± 0.26	4.21 ± 0.51
Rap2b	$Rap2b^{tm1.1(KOMP)Vlcg}/J^{(+/-)}$	RRID:IMSR_JAX:025590	8	2.31 ± 0.47	2.88 ± 0.44	2.71 ± 0.45	5.1 ± 0.37
Stx19	$Stx19^{tm1.1(KOMP)Vlcg}/2J^{(-/-)}$	RRID:MGI:5603919	8	1.28 ± 0.52	1.59 ± 0.35	1.86 ± 0.47	3.29 ± 0.48
Zbtb4	$Zbtb4^{tm1.1(KOMP)Vlcg}/J^{(-/-)}$	RRID:IMSR_JAX:023660	8	1.79 ± 0.32	2.62 ± 0.42	2.05 ± 0.32	4.79 ± 0.44
C3	$C3^{tm1.1(KOMP)Vlcg}/J^{(-/-)}$	RRID:IMSR_JAX:022098	7	1.73 ± 0.32	2.18 ± 0.52	2.19 ± 0.49	3.41 ± 0.69
Tmod2	$Tmod2^{tm1b(KOMP)Wtsi}/2J^{(-/-)}$	RRID:IMSR_JAX:024939	8	1.14 ± 0.39	2.52 ± 0.34	2.3 ± 0.27	5.02 ± 0.7
Myh10	$Myh10^{em1(IMPC)J}/J^{(+/-)}$	RRID:MGI:5884012	8	1.94 ± 0.25	2.75 ± 0.41	2.77 ± 0.3	3.94 ± 0.41
Stk36	$Stk36^{em1(IMPC)J}/J^{(-/-)}$	RRID:MGI:5884030	8	1.19 ± 0.5	1.49 ± 0.46	1.65 ± 0.43	3.35 ± 0.77
Dnmt3a	$Dnmt3a^{tm1b(KOMP)Wtsi}/J^{(+/-}$)	RRID:IMSR_JAX:018838	5	1.4 ± 0.34	1.75 ± 0.71	1.8 ± 0.55	3.35 ± 1.02
Gpr142	$Gpr142^{tm1.1(KOMP)Vlcg}/J^{(-/-)}$	RRID:IMSR_JAX:026065	8	2.08 ± 0.27	2.06 ± 0.41	2.27 ± 0.33	3.81 ± 0.38
Htr7	$Htr7^{tm1b(KOMP)Wtsi}/J^{(-/-)}$	RRID:IMSR_JAX:023282	8	1.86 ± 0.19	2.31 ± 0.25	2.18 ± 0.4	3.43 ± 0.49
Irf8	$Irf8^{tm1b(KOMP)Wtsi}/J^{(-/-)}$	RRID:IMSR_JAX:018655	8	1.41 ± 0.4	2.16 ± 0.5	1.66 ± 0.32	2.66 ± 0.53

Supplemental Table 8

	Hole Board		Light Dark		Open Field		Startle		TST	
	Female	Male	Female	Male	Female	Male	Female	Male	Female	Male
Cp	8	5	8	5	16	10	24	15	6	5
Stk36	8	7	8	7	8	8	8	7		
Dnaja4	9	8	9	7	2	3	2	3	2	3
Zbtb4	5	7	5	6	10	13	5	7	5	7
Stx19	8	8	8	7	8	8	8	8		
Myh10	8	7	8	7	8	7	8	7		
Irf8	8	8	8	8	24	24	8	8	6	5
C3	8	8	16	16	8	8	8	8	8	8
Gpr142	8	8	8	8	8	8	4	3		
Dnmt3a	8	6	8	6	24	9	8	7	8	5
Lrrc15	8	8	8	8	8	8	8	8	2	2
Htr7	4	6	7	7	7	7	1	3	1	3
Rap2b	11	10	11	10	10	10	4	2		
Elof1	8	9	8	9	8	9	8	9	8	8
Tmod2	8	8	8	8	8	8	8	8		
Dnajb3	8	9	8	9	18	12	8	9	8	9
Hspb2	6	8	6	8	18	24	5	6	6	7
Lpar6	8	8	16	12	4	6	8	8	8	8
Il12rb2	8	5	16	10	6	4	8	5	7	4
Pitx3	8	6	16	12	2	2	7	6	8	6
Htr1a	8	8	8	8	24	24	8	8	7	4
Pnmt	7	8	8	8	8	8	7	8	8	8
Far2	8	10	16	20	8	10	8	10	8	8
Btg2	8	5	16	10	8	5	8	5	8	5
Parp8	8	8	8	7	8	8	8	8	7	8
C9	8	10			8	8	8	9	8	9
Gipc3	8	8	8	8	24	12	8	8	8	7
Rilpl2	5	8	10	16	15	24	5	8	5	8
C1qa	8	8	16	16	6	5	8	8	8	8
Epb41l4a	8	7	16	14	8	7	8	7	5	4
Hdac10	8	7	8	7	12	9	8	7	4	3
Dnase1l2	8	7	16	14	24	21	8	7	8	6

Supplemental Table 9

Ethanol		3%						6%					
**Female**	n=	Amount of Drug Consumed	Amount of Water Cosnumed	Drug Weight	Preference (decimal %)	Total Drinking (g/kg bodyweight)	Water Drinking	Amount of Drug Consumed	Amount of Water Cosnumed	Drug Weight	Preference (decimal %)	Total Drinking (g/kg bodyweight)	Water Drinking
C57Bl6/6NJ	39	3.39 ± 0.23	2.97 ± 0.27	4.44 ± 0.3	0.54 ± 0.03	277.85 ± 14.84	129.82 ± 12.06	3.63 ± 0.31	2.57 ± 0.23	9.56 ± 0.82	0.57 ± 0.03	274.01 ± 13.61	111.99 ± 9.58
Btg2	8	3.84 ± 0.66	3.94 ± 0.7	5.53 ± 0.95	0.5 ± 0.08	373.88 ± 20.89	189.63 ± 33.97	3.33 ± 0.48	3.74 ± 0.58	9.57 ± 1.35	0.48 ± 0.07	340.63 ± 16.96	181.16 ± 29.49
C1qa	8	2.22 ± 0.46	4.4 ± 0.67	2.96 ± 0.63	0.34 ± 0.07	293.11 ± 22.52	194.32 ± 28.43	2.61 ± 0.4	3.29 ± 0.53	6.91 ± 1.02	0.45 ± 0.07	260.11 ± 16.04	144.92 ± 22.18
C9	0	n.d.	n.d.	n.d.	n.d.	n.d.	n.d.	n.d.	n.d.	n.d.	n.d.	n.d.	n.d.
Cfb	8	2.22 ± 0.33	2.29 ± 0.28	3.53 ± 0.59	0.49 ± 0.06	241.24 ± 25.26	123.68 ± 17.16	2.41 ± 0.26	1.97 ± 0.35	7.61 ± 0.83	0.57 ± 0.06	234.22 ± 22.22	107.41 ± 22.11
Cp	8	3.98 ± 0.65	2.04 ± 0.51	5.99 ± 1.01	0.64 ± 0.1	301.03 ± 14.96	101.23 ± 24.99	3.66 ± 0.56	2.43 ± 0.48	10.98 ± 1.7	0.59 ± 0.08	303.57 ± 17.39	120.51 ± 22.9
Dnajb3	8	3.45 ± 0.57	1.53 ± 0.47	4.9 ± 0.8	0.69 ± 0.09	235.37 ± 15.62	72.12 ± 22.29	3.49 ± 0.93	1.9 ± 0.83	9.91 ± 2.65	0.64 ± 0.12	254.88 ± 38.89	89.76 ± 39.47
Dnase112	8	2.55 ± 0.35	2.73 ± 0.63	3.82 ± 0.56	0.51 ± 0.07	259.31 ± 29.78	131.96 ± 28.91	3.41 ± 0.67	2.85 ± 0.56	10.2 ± 2.06	0.54 ± 0.08	308.55 ± 24.41	138.48 ± 25.6
Epb4.114a	8	2.48 ± 0.58	2.38 ± 0.57	3.31 ± 0.72	0.51 ± 0.09	218.82 ± 24.34	108.65 ± 26.06	3.07 ± 0.68	2.99 ± 0.53	8.27 ± 1.84	0.49 ± 0.1	274.3 ± 11.63	136.43 ± 25
Far2	8	3.15 ± 0.66	4.5 ± 0.76	3.86 ± 0.78	0.42 ± 0.08	313.62 ± 20.88	185.02 ± 32.17	2.3 ± 0.6	4.18 ± 0.56	5.81 ± 1.61	0.36 ± 0.08	266.54 ± 24.82	164.9 ± 18.16
Gipc3	7	3.38 ± 0.55	2.51 ± 0.64	4.25 ± 0.74	0.6 ± 0.07	247.77 ± 42.41	106.05 ± 27.79	3.08 ± 0.9	2.31 ± 0.72	7.77 ± 2.21	0.57 ± 0.11	227.96 ± 33.22	98.44 ± 30.16
Hdac10	8	2.86 ± 0.55	1.84 ± 0.36	3.7 ± 0.69	0.59 ± 0.06	202.6 ± 23	79.34 ± 15.17	3.06 ± 0.82	1.19 ± 0.49	7.84 ± 2.04	0.66 ± 0.11	184.95 ± 28.49	54.23 ± 23.12
Hspb2	8	2.87 ± 0.67	3.39 ± 0.7	4.11 ± 0.97	0.46 ± 0.08	295.65 ± 38.15	158.62 ± 29.87	3.75 ± 0.93	2.02 ± 0.44	10.56 ± 2.58	0.6 ± 0.08	271.94 ± 40.27	95.94 ± 21.64
Htr1a	8	3.56 ± 0.57	2.89 ± 0.68	3.96 ± 0.65	0.58 ± 0.06	241.3 ± 41.34	109.39 ± 26.82	2.08 ± 0.34	3.78 ± 0.35	4.68 ± 0.8	0.35 ± 0.05	218.26 ± 11.6	140.19 ± 12.83
Il12rb2	8	2.71 ± 0.59	1.84 ± 0.54	3.88 ± 0.84	0.59 ± 0.08	217.27 ± 27.87	88 ± 25.74	3.16 ± 0.8	1.33 ± 0.58	9.05 ± 2.28	0.67 ± 0.12	214.22 ± 28.08	63.31 ± 27.44
Lpar6	3	0.97 ± 0.23	5.51 ± 0.75	1.41 ± 0.37	0.15 ± 0.02	312.17 ± 55.96	265.26 ± 45.16	2.5 ± 0.8	3.03 ± 0.41	7.13 ± 2.19	0.43 ± 0.12	264.59 ± 27.63	145.71 ± 22.89
Parp8	6	2.71 ± 0.52	2.6 ± 0.6	4.04 ± 0.81	0.52 ± 0.09	260.33 ± 22.09	125.83 ± 27.41	2.98 ± 1.01	2.29 ± 0.94	8.74 ± 2.94	0.56 ± 0.13	257.4 ± 42.55	111.67 ± 45.11
Pitx3	2	3.67 ± 0.58	3.31 ± 1.09	6.04 ± 0.7	0.53 ± 0.12	386.84 ± 44.37	185.45 ± 67.86	2.26 ± 0.69	3.88 ± 0.73	7.4 ± 1.96	0.37 ± 0.11	339.69 ± 16.55	216.41 ± 49.3
Pnmt	3	3.74 ± 1.41	3.08 ± 1.88	5.04 ± 1.94	0.58 ± 0.23	295.86 ± 8.51	127.96 ± 72.63	3.92 ± 1.6	1.89 ± 1.11	10.46 ± 4.25	0.65 ± 0.22	253.03 ± 33.13	78.66 ± 42.84
Rilp12	8	2.22 ± 0.47	2.33 ± 0.57	2.71 ± 0.59	0.51 ± 0.08	182.45 ± 23.03	92.2 ± 21.17	3.19 ± 0.77	1.43 ± 0.47	7.81 ± 1.91	0.65 ± 0.09	185.69 ± 27.13	55.55 ± 16.71
**Male**													
C57Bl6/6NJ	38	3.1 ± 0.25	3.73 ± 0.26	3.31 ± 0.28	0.45 ± 0.03	241.88 ± 9.34	131.6 ± 9	2.66 ± 0.29	3.89 ± 0.28	5.59 ± 0.6	0.41 ± 0.03	228.97 ± 11.84	135.87 ± 9.67
Btg2	7	1.92 ± 0.4	4.52 ± 0.32	2.12 ± 0.44	0.29 ± 0.05	237.45 ± 9.48	166.76 ± 10.09	1.67 ± 0.42	4.59 ± 0.38	3.65 ± 0.89	0.26 ± 0.06	231.17 ± 5.75	170.4 ± 15.01
C1qa	7	2.97 ± 0.58	4.47 ± 0.68	3.4 ± 0.63	0.4 ± 0.08	287.53 ± 15.48	174.14 ± 26.92	3.59 ± 0.52	3.34 ± 0.26	8.33 ± 1.22	0.52 ± 0.05	278.81 ± 20.07	131.43 ± 13.06
C9	0	n.d.	n.d.	n.d.	n.d.	n.d.	n.d.	n.d.	n.d.	n.d.	n.d.	n.d.	n.d.
Cfb	8	2.17 ± 0.44	2.33 ± 0.51	2.78 ± 0.64	0.5 ± 0.09	192.1 ± 20.94	99.47 ± 22.32	2.03 ± 0.35	2.64 ± 0.57	5.07 ± 0.85	0.46 ± 0.08	198.19 ± 17.04	113.74 ± 25.92
Cp	8	2.34 ± 0.41	3.1 ± 0.53	2.98 ± 0.5	0.45 ± 0.07	231 ± 20.37	131.57 ± 22.61	2.96 ± 0.5	2.89 ± 0.5	7.57 ± 1.26	0.51 ± 0.08	248.68 ± 14.37	122.46 ± 21.34
Dnajb3	8	2.14 ± 0.22	1.6 ± 0.36	2.81 ± 0.29	0.59 ± 0.03	163.75 ± 23.66	70.03 ± 15.79	2.92 ± 0.68	2.32 ± 0.9	7.68 ± 1.78	0.6 ± 0.14	229.54 ± 20.89	101.52 ± 39.23
Dnase112	7	2.55 ± 0.65	3.7 ± 0.38	3.02 ± 0.75	0.39 ± 0.07	246 ± 23.96	145.25 ± 13.56	3.28 ± 0.73	3.99 ± 0.51	7.73 ± 1.6	0.44 ± 0.05	285.32 ± 36.48	156.52 ± 17.95
Epb4.114a	8	2.78 ± 0.31	2.73 ± 0.32	3.57 ± 0.42	0.5 ± 0.05	235.07 ± 17.96	116.23 ± 13.56	3.43 ± 0.66	2.03 ± 0.56	8.77 ± 1.7	0.62 ± 0.11	232.51 ± 13.05	86.31 ± 23.82
Far2	5	2.44 ± 1.08	4.68 ± 1.16	2.5 ± 1.05	0.35 ± 0.15	255.21 ± 17.45	171.82 ± 44.78	1.87 ± 0.66	4.93 ± 0.87	3.91 ± 1.31	0.29 ± 0.11	243.73 ± 13.46	178.65 ± 34.26
Gipc3	8	2.68 ± 0.47	3.17 ± 0.52	3.01 ± 0.53	0.46 ± 0.07	218.91 ± 17.31	118.45 ± 19.37	3.05 ± 0.76	2.71 ± 0.73	6.89 ± 1.76	0.53 ± 0.11	215.57 ± 23.36	100.66 ± 26.93
Hdac10	8	3.37 ± 0.61	1.33 ± 0.26	4.35 ± 0.8	0.68 ± 0.06	202.02 ± 23.62	57.09 ± 11.05	3.5 ± 0.86	2.59 ± 1.09	9.03 ± 2.24	0.61 ± 0.14	261.97 ± 24.64	111.42 ± 46.75
Hspb2	3	1.92 ± 0.17	2.8 ± 0.86	2.48 ± 0.22	0.44 ± 0.1	202.78 ± 38.47	120.21 ± 37.02	3.23 ± 1.9	0.44 ± 0.13	8.33 ± 4.9	0.79 ± 0.09	157.88 ± 76.53	18.97 ± 5.45
Htr1a	8	1.7 ± 0.36	3.32 ± 0.63	1.8 ± 0.39	0.35 ± 0.06	182.14 ± 34.32	122.19 ± 26.49	1.85 ± 0.53	4.12 ± 0.56	3.91 ± 1.13	0.3 ± 0.05	213.38 ± 33.86	148.25 ± 22.56
Il12rb2	7	3.04 ± 0.77	2.59 ± 0.52	4.11 ± 1.04	0.51 ± 0.11	253.76 ± 20.86	116.7 ± 23.34	4.75 ± 0.96	0.84 ± 0.36	12.84 ± 2.6	0.8 ± 0.1	251.66 ± 34	37.69 ± 16.06
Lpar6	3	1.07 ± 0.28	4.7 ± 0.12	1.33 ± 0.3	0.18 ± 0.04	240.2 ± 10.87	196.02 ± 9.27	1.33 ± 0.14	4.83 ± 0.37	3.32 ± 0.27	0.22 ± 0.02	255.48 ± 1.29	200.22 ± 3.26
Parp8	5	3.16 ± 1.58	3.71 ± 1.47	4.35 ± 2.21	0.44 ± 0.21	307.02 ± 40.67	161.87 ± 66.67	2.26 ± 1.1	3.43 ± 0.91	6.21 ± 3.08	0.37 ± 0.17	254.51 ± 20.55	150.97 ± 40.44
Pitx3	2	2.74 ± 0.64	3.28 ± 0.43	3.87 ± 1.05	0.45 ± 0.09	280.76 ± 21.94	151.88 ± 13.22	1.33 ± 0.3	5.14 ± 0.41	3.76 ± 1.01	0.2 ± 0.02	302.71 ± 46.3	240.05 ± 29.49
Pnmt	3	1.01 ± 0.35	4.14 ± 0.25	1.14 ± 0.36	0.19 ± 0.05	197.64 ± 12.74	159.58 ± 2.24	1.01 ± 0.3	4.56 ± 0.26	2.28 ± 0.58	0.18 ± 0.05	215.11 ± 4.79	177.19 ± 13.53
Rilp12	8	4.17 ± 0.71	2.3 ± 0.57	4.64 ± 0.78	0.63 ± 0.09	239.91 ± 14.84	85.34 ± 21.21	3.65 ± 0.89	1.49 ± 0.65	8.14 ± 1.99	0.68 ± 0.11	190.67 ± 24.82	55.02 ± 23.97

		12%						15%					
**Female**	n=	Amount of Drug Consumed	Amount of Water Cosnumed	Drug Weight	Preference (decimal %)	Total Drinking (g/kg bodyweight)	Water Drinking	Amount of Drug Consumed	Amount of Water Cosnumed	Drug Weight	Preference (decimal %)	Total Drinking (g/kg bodyweight)	Water Drinking
C57Bl6/6NJ	39	5.01 ± 0.22	2.71 ± 0.23	26.32 ± 1.16	0.65 ± 0.03	336.94 ± 7.73	117.58 ± 9.57	4.01 ± 0.28	2.42 ± 0.23	26.34 ± 1.87	0.62 ± 0.03	280.41 ± 10.48	104.84 ± 9.68
Btg2	8	6.17 ± 0.79	2.79 ± 0.54	35.52 ± 4.62	0.68 ± 0.06	429.95 ± 28.54	133.97 ± 25.63	4.47 ± 0.24	1.33 ± 0.19	32.24 ± 1.89	0.77 ± 0.03	278.85 ± 12.49	63.94 ± 9.2
C1qa	8	4 ± 0.43	2.97 ± 0.5	21.24 ± 2.24	0.58 ± 0.06	308.35 ± 10.06	131.33 ± 21.24	2.17 ± 0.41	2.51 ± 0.39	14.22 ± 2.49	0.46 ± 0.08	206.68 ± 5.41	111.91 ± 17.64
C9	0	n.d.	n.d.	n.d.	n.d.	n.d.	n.d.	n.d.	n.d.	n.d.	n.d.	n.d.	n.d.
Cfb	8	3.79 ± 0.51	1.98 ± 0.43	24.02 ± 3.1	0.65 ± 0.08	305.17 ± 20.92	105 ± 23	2.48 ± 0.22	1.84 ± 0.13	19.69 ± 1.86	0.57 ± 0.03	227.4 ± 10.63	96.13 ± 4.75
Cp	8	6.26 ± 0.69	1.91 ± 0.34	37.43 ± 3.8	0.75 ± 0.05	407.9 ± 21.54	95.96 ± 17.05	3.51 ± 0.48	1.53 ± 0.34	26.12 ± 3.39	0.68 ± 0.08	251.8 ± 8.27	77.68 ± 17.9
Dnajb3	8	7.52 ± 0.43	0.54 ± 0.06	42.65 ± 2.46	0.93 ± 0.01	381.21 ± 19.52	25.77 ± 2.96	7.21 ± 0.6	1.19 ± 0.55	51.15 ± 4.28	0.86 ± 0.06	397.5 ± 11.84	56.48 ± 25.78
Dnase112	8	3.72 ± 0.36	4 ± 0.44	22.17 ± 2.35	0.48 ± 0.05	380.53 ± 14.29	195.78 ± 18.89	3.81 ± 0.36	1.8 ± 0.26	28.3 ± 2.73	0.68 ± 0.05	276.61 ± 12.99	87.94 ± 11.26
Epb4.114a	8	5.26 ± 0.4	2.08 ± 0.46	28.76 ± 2.38	0.72 ± 0.06	332.94 ± 7.8	93.26 ± 20.1	3.7 ± 0.55	2.2 ± 0.41	25.45 ± 3.94	0.61 ± 0.08	268.42 ± 16.15	98.77 ± 17.91
Far2	8	4.52 ± 0.48	4.19 ± 0.48	22.61 ± 2.99	0.52 ± 0.05	359.3 ± 20.85	170.91 ± 20.32	2.62 ± 0.54	3.14 ± 0.47	16.4 ± 3.71	0.45 ± 0.08	237.96 ± 16.17	128.66 ± 21.22
Gipc3	7	5.43 ± 0.58	2.28 ± 0.55	27.1 ± 2.57	0.71 ± 0.07	324.09 ± 16.19	98.27 ± 24.57	3.98 ± 0.91	2.07 ± 0.71	24.46 ± 5.42	0.61 ± 0.14	253.27 ± 14.18	90.6 ± 32.08
Hdac10	8	5.94 ± 0.16	0.89 ± 0.17	30.83 ± 0.71	0.87 ± 0.02	296.15 ± 9.7	39.26 ± 8.11	4.84 ± 0.74	1.82 ± 0.68	30.82 ± 4.49	0.72 ± 0.09	284.19 ± 17.36	78.71 ± 28.76
Hspb2	8	5.53 ± 0.76	2.05 ± 0.42	30.81 ± 3.85	0.71 ± 0.06	354.35 ± 19.29	97.61 ± 21.27	4.38 ± 0.54	1.5 ± 0.32	30.78 ± 3.67	0.75 ± 0.03	274.55 ± 33.25	69.37 ± 13.93
Htr1a	8	5.13 ± 0.62	2.2 ± 0.5	22.89 ± 2.98	0.7 ± 0.07	274.55 ± 18.36	83.76 ± 19.87	2.82 ± 0.45	1.71 ± 0.26	15.58 ± 2.31	0.61 ± 0.07	168.43 ± 10.93	64.57 ± 10.63
Il12rb2	8	7.29 ± 0.34	0.85 ± 0.21	41.72 ± 1.95	0.9 ± 0.03	388.13 ± 13.86	40.44 ± 10.23	7.1 ± 0.58	1.08 ± 0.18	50.8 ± 4.14	0.87 ± 0.02	390.32 ± 28.77	51.68 ± 8.78
Lpar6	3	4.75 ± 0.99	2.64 ± 1	27.36 ± 5.83	0.64 ± 0.14	353.08 ± 16.62	125.07 ± 45.56	2.14 ± 0.24	2.17 ± 0.44	14.84 ± 1.42	0.5 ± 0.08	199.81 ± 12.42	100.85 ± 21.92
Parp8	6	5.72 ± 0.99	1.89 ± 0.6	33.55 ± 5.68	0.74 ± 0.08	372.13 ± 37.08	92.57 ± 28.76	5.73 ± 1.08	1.17 ± 0.35	42.17 ± 7.81	0.8 ± 0.07	337.22 ± 43.27	56.11 ± 14.94
Pitx3	2	4.42 ± 1.3	3.24 ± 1.07	29.69 ± 9.88	0.57 ± 0.15	423.95 ± 30.59	176.53 ± 51.75	2.68 ± 0.6	2.46 ± 0.85	22.42 ± 5.95	0.53 ± 0.14	283.67 ± 1.7	134.19 ± 41.39
Pnmt	3	5.05 ± 1.59	2.22 ± 1.23	27.3 ± 9.41	0.68 ± 0.19	320.26 ± 39.44	92.8 ± 47.02	1.99 ± 0.2	2.2 ± 0.83	13.21 ± 2.07	0.51 ± 0.11	180.49 ± 21.85	92.44 ± 30.46
Rilp12	8	5.43 ± 0.56	1.45 ± 0.4	26.56 ± 2.92	0.78 ± 0.06	278.31 ± 13.95	57.01 ± 14.15	5.64 ± 0.53	1.23 ± 0.26	34.48 ± 3.5	0.81 ± 0.05	278.6 ± 18.45	48.76 ± 9.53
**Male**													
C57Bl6/6NJ	38	4.51 ± 0.33	3.28 ± 0.33	19.39 ± 1.49	0.58 ± 0.04	275.13 ± 6.16	113.56 ± 11.1	3.26 ± 0.38	3.07 ± 0.23	17.37 ± 1.99	0.49 ± 0.04	222.74 ± 12.88	106.9 ± 7.43
Btg2	7	3.81 ± 0.47	4.25 ± 0.73	17.09 ± 2.22	0.49 ± 0.07	298.13 ± 13	155.74 ± 25.58	2.36 ± 0.46	3.13 ± 0.47	13.07 ± 2.55	0.43 ± 0.09	203.04 ± 7.95	115.91 ± 17.19
C1qa	7	5.24 ± 0.66	3.25 ± 0.69	24.12 ± 2.77	0.62 ± 0.07	329.53 ± 25.69	128.53 ± 29.49	3.33 ± 0.35	2.07 ± 0.4	19.25 ± 1.92	0.62 ± 0.06	209.69 ± 14.64	81.32 ± 16.49
C9	0	n.d.	n.d.	n.d.	n.d.	n.d.	n.d.	n.d.	n.d.	n.d.	n.d.	n.d.	n.d.
Cfb	8	2.95 ± 0.52	3.1 ± 0.51	15.59 ± 3.12	0.49 ± 0.08	257.48 ± 13.75	127.53 ± 19.31	1.92 ± 0.26	2.33 ± 0.3	12.38 ± 2.11	0.46 ± 0.07	177.72 ± 11.16	95.21 ± 11.78
Cp	8	4.52 ± 0.56	3.03 ± 0.69	23.36 ± 2.89	0.61 ± 0.08	323.63 ± 13.15	128.94 ± 28.02	2.41 ± 0.23	2.32 ± 0.48	15.65 ± 1.68	0.53 ± 0.07	202.6 ± 9.9	98.27 ± 19.14
Dnajb3	8	6 ± 0.82	1.42 ± 0.44	31.52 ± 4.3	0.79 ± 0.08	324.88 ± 20.62	62.18 ± 19.42	5.87 ± 0.69	1.14 ± 0.21	38.55 ± 4.51	0.83 ± 0.03	307 ± 28.53	49.98 ± 9.3
Dnase112	7	5.19 ± 0.54	2.25 ± 0.46	24.52 ± 2.36	0.69 ± 0.07	294.52 ± 6.85	90.22 ± 19.23	2.39 ± 0.29	2.97 ± 0.37	14.41 ± 1.96	0.45 ± 0.05	212.54 ± 6.49	116.46 ± 12.8
Epb4.114a	8	5.06 ± 0.8	1.86 ± 0.4	25.75 ± 4.07	0.7 ± 0.08	294.53 ± 22.2	79.95 ± 17.67	3.8 ± 0.8	1.94 ± 0.48	24.34 ± 5.16	0.63 ± 0.1	244.49 ± 21.23	82.2 ± 19.69
Far2	5	2.84 ± 0.89	5.56 ± 1.43	12.05 ± 3.8	0.37 ± 0.14	300.83 ± 23.38	200.37 ± 53.39	1.39 ± 0.5	4.97 ± 0.67	7.54 ± 2.85	0.22 ± 0.08	225.57 ± 7.02	175.29 ± 19.29
Gipc3	8	3.25 ± 0.7	3.78 ± 0.97	14.65 ± 3.25	0.49 ± 0.11	263.41 ± 18.26	141.3 ± 36.56	2.76 ± 0.92	3.47 ± 0.89	15.68 ± 5.29	0.42 ± 0.13	234.05 ± 26.72	129.51 ± 33.6
Hdac10	8	5.71 ± 0.39	1.53 ± 0.55	29.51 ± 2.05	0.8 ± 0.06	311.94 ± 15.61	66.04 ± 23.9	4.24 ± 0.54	2.03 ± 0.5	27.39 ± 3.48	0.68 ± 0.07	269.88 ± 21.54	87.27 ± 21.69
Hspb2	3	7.42 ± 0.64	0.8 ± 0.16	38.27 ± 3.28	0.9 ± 0.01	353.25 ± 33.06	34.37 ± 6.99	6.88 ± 0.56	1.04 ± 0.27	44.33 ± 3.63	0.87 ± 0.04	340.37 ± 15.81	44.82 ± 11.72
Htr1a	8	2.96 ± 0.56	3.27 ± 0.45	12.68 ± 2.73	0.44 ± 0.09	210.49 ± 16.58	115.65 ± 18.68	1.58 ± 0.21	2.71 ± 0.26	8.6 ± 1.35	0.37 ± 0.05	153.35 ± 11.73	96.03 ± 9.68
Il12rb2	7	6.72 ± 0.75	1.33 ± 0.49	36.33 ± 4.07	0.83 ± 0.06	362.44 ± 22.28	59.67 ± 22.14	7.1 ± 0.42	0.79 ± 0.07	47.96 ± 2.84	0.9 ± 0.01	355.39 ± 17.03	35.63 ± 3.35
Lpar6	3	3.35 ± 0.45	4.1 ± 0.75	17.18 ± 3.65	0.46 ± 0.08	310.29 ± 11.16	167.11 ± 21.48	1.44 ± 0.31	3.68 ± 0.38	8.98 ± 0.87	0.28 ± 0.02	215.35 ± 3.33	155.5 ± 2.47
Parp8	5	5.19 ± 1.3	1.87 ± 0.6	28.42 ± 7.45	0.69 ± 0.11	318.71 ± 43.4	81.86 ± 26.03	4.73 ± 1.37	2.16 ± 0.63	32.6 ± 9.61	0.61 ± 0.13	312.03 ± 60.01	94.67 ± 27.91
Pitx3	2	3.38 ± 0.4	3.69 ± 0.61	18.95 ± 3.04	0.48 ± 0.07	328.44 ± 4.2	170.48 ± 21.14	2.15 ± 0.78	2.57 ± 1.05	15.22 ± 6.06	0.47 ± 0.19	219.14 ± 3.46	117.68 ± 43.86
Pnmt	3	2.9 ± 0.63	4.29 ± 0.84	13.84 ± 3.7	0.41 ± 0.1	277.52 ± 10.06	162.17 ± 22.88	0.97 ± 0.13	3.65 ± 0.31	5.57 ± 0.53	0.21 ± 0.01	177.67 ± 8.09	140.52 ± 4.59
Rilp12	8	5.32 ± 0.88	1.86 ± 0.75	23.76 ± 3.94	0.73 ± 0.11	266.87 ± 12.42	68.89 ± 27.71	4.28 ± 0.67	2.26 ± 0.57	23.91 ± 3.77	0.64 ± 0.09	243.01 ± 12.47	83.61 ± 21.1

Supplemental Table 10

Methamphetamine		10 mg/L						20 mg/L					
**Female**	n=	Amount of Drug Consumed	Amount of Water Cosnumed	Drug Weight	Preference (decimal %)	Total Drinking (g/kg bodyweight)	Water Drinking	Amount of Drug Consumed	Amount of Water Cosnumed	Drug Weight	Preference (decimal %)	Total Drinking (g/kg bodyweight)	Water Drinking
C57Bl6/6NJ	29	3.59 ± 0.31	2.9 ± 0.37	0.0016 ± 0.0001	0.57 ± 0.05	288.19 ± 13.98	126.29 ± 17.4	1.78 ± 0.35	5.32 ± 0.35	0.0016 ± 0.0003	0.24 ± 0.04	320.34 ± 13.57	239.24 ± 16.61
Btg2	8	3.42 ± 0.62	3.58 ± 0.41	0.0015 ± 0.0003	0.47 ± 0.07	316.31 ± 19.51	161.6 ± 17.15	1.92 ± 0.52	3.28 ± 0.53	0.0018 ± 0.0005	0.37 ± 0.1	237.52 ± 15.81	148.17 ± 24.05
C1qa	8	3.09 ± 0.48	3.05 ± 0.4	0.0015 ± 0.0002	0.5 ± 0.07	295.12 ± 8.54	146.71 ± 18.08	2.01 ± 0.38	3.42 ± 0.41	0.0019 ± 0.0003	0.37 ± 0.05	259.77 ± 24.06	163.5 ± 18.35
C9	7	1.74 ± 0.26	3.15 ± 0.44	0.0008 ± 0.0001	0.39 ± 0.06	242.06 ± 19.16	152.53 ± 25.63	1.3 ± 0.13	5.43 ± 0.23	0.0013 ± 0.0002	0.19 ± 0.02	328.13 ± 24.5	264.03 ± 18.33
Cfb	5	2.55 ± 1.09	3.17 ± 0.57	0.0013 ± 0.0006	0.4 ± 0.12	298.08 ± 34.36	163.56 ± 28.83	1.76 ± 0.61	3.05 ± 0.54	0.0019 ± 0.0007	0.36 ± 0.1	250.2 ± 25.74	157.38 ± 27.47
Cp	11	2.49 ± 0.42	3.63 ± 0.69	0.0011 ± 0.0002	0.45 ± 0.09	244.76 ± 28.08	139.61 ± 30.73	1.53 ± 0.22	5.13 ± 0.39	0.0012 ± 0.0002	0.23 ± 0.04	275.15 ± 7.76	212.66 ± 13.71
Dnajb3	5	2.64 ± 0.5	3.54 ± 0.74	0.0012 ± 0.0002	0.44 ± 0.09	291.14 ± 40.75	171.32 ± 46.92	1.6 ± 0.61	3.54 ± 0.53	0.0016 ± 0.0007	0.3 ± 0.11	239.07 ± 23.34	157.26 ± 18.39
Dnase112	8	2.4 ± 0.64	5.22 ± 0.85	0.0012 ± 0.0003	0.33 ± 0.1	389.1 ± 25.44	267.97 ± 44.37	3.11 ± 1.23	3.6 ± 0.83	0.0034 ± 0.0014	0.4 ± 0.12	345.21 ± 42.73	177.23 ± 37.42
Epb4.114a	8	3.04 ± 0.8	3.23 ± 0.78	0.0015 ± 0.0004	0.48 ± 0.12	313.66 ± 24.71	165.21 ± 43.15	2.82 ± 1.5	4.61 ± 0.99	0.0028 ± 0.0016	0.33 ± 0.1	376.11 ± 63.99	233.83 ± 54.99
Far2	7	4.36 ± 1.05	1.97 ± 0.79	0.002 ± 0.0005	0.67 ± 0.11	288.15 ± 35.98	86.86 ± 32.76	7.98 ± 1.51	1.63 ± 0.59	0.0076 ± 0.0016	0.79 ± 0.1	453.51 ± 61.92	73.49 ± 25.33
Gipc3	8	2.4 ± 0.31	3.72 ± 0.44	0.001 ± 0.0001	0.4 ± 0.06	252.97 ± 15.37	152.95 ± 18.12	1.48 ± 0.25	5.08 ± 0.43	0.0012 ± 0.0002	0.23 ± 0.04	269.29 ± 14.85	208.09 ± 15.73
Hdac10	7	2.51 ± 0.31	3.56 ± 0.84	0.0011 ± 0.0001	0.46 ± 0.09	267.51 ± 25.17	156.57 ± 36.69	1.63 ± 0.21	5.5 ± 0.25	0.0014 ± 0.0002	0.23 ± 0.02	314.53 ± 16.48	242.69 ± 11.77
Hspb2	8	2.39 ± 0.39	3.31 ± 0.71	0.0012 ± 0.0002	0.45 ± 0.07	278.15 ± 22.56	157.8 ± 30.09	1.78 ± 0.34	2.62 ± 0.3	0.0017 ± 0.0003	0.39 ± 0.03	213.5 ± 21.56	128.05 ± 13.32
Htr1a	11	2.18 ± 0.37	3.62 ± 0.69	0.0009 ± 0.0002	0.42 ± 0.06	250.38 ± 30.88	155.84 ± 28.81	1.31 ± 0.21	4.85 ± 0.96	0.0011 ± 0.0002	0.27 ± 0.05	252.35 ± 43.8	196.33 ± 45.14
Il12rb2	8	3.54 ± 0.72	2.2 ± 0.84	0.0018 ± 0.0004	0.64 ± 0.13	281.62 ± 24.49	98.08 ± 36.68	2.56 ± 0.93	3.65 ± 0.93	0.0028 ± 0.0011	0.41 ± 0.15	306.37 ± 27.84	168.39 ± 41.64
Lpar6	0	n.d.	n.d.	n.d.	n.d.	n.d.	n.d.	n.d.	n.d.	n.d.	n.d.	n.d.	n.d.
Parp8	8	3.07 ± 0.52	3.17 ± 0.56	0.0015 ± 0.0003	0.5 ± 0.08	318.51 ± 16.64	164.19 ± 32.15	1.91 ± 0.28	3.24 ± 0.43	0.0019 ± 0.0003	0.38 ± 0.07	261.59 ± 11.31	164.36 ± 22.12
Pitx3	5	2.35 ± 0.33	2.44 ± 0.65	0.0014 ± 0.0002	0.51 ± 0.07	294.17 ± 50.59	150.56 ± 46.49	1.54 ± 0.28	3.83 ± 0.39	0.0019 ± 0.0004	0.29 ± 0.06	325.31 ± 10.53	228.47 ± 13.21
Pnmt	0	n.d.	n.d.	n.d.	n.d.	n.d.	n.d.	n.d.	n.d.	n.d.	n.d.	n.d.	n.d.
Rilp12	8	2.8 ± 0.43	4.22 ± 0.74	0.0013 ± 0.0002	0.42 ± 0.06	321.14 ± 22.43	193.14 ± 32.99	1.94 ± 0.34	3.77 ± 0.45	0.0018 ± 0.0003	0.35 ± 0.06	262.18 ± 17.87	173.41 ± 21.19
**Male**													
C57Bl6/6NJ	31	3.34 ± 0.29	3.88 ± 0.42	0.0013 ± 0.0001	0.49 ± 0.05	268.06 ± 12.07	143.04 ± 16.07	2.39 ± 0.28	3.87 ± 0.37	0.0018 ± 0.0002	0.4 ± 0.04	232.44 ± 10.4	142.64 ± 13.63
Btg2	8	2.34 ± 0.34	3.77 ± 0.3	0.0009 ± 0.0001	0.38 ± 0.05	236.12 ± 14.36	146.69 ± 13.99	1.39 ± 0.28	3.93 ± 0.24	0.0011 ± 0.0002	0.26 ± 0.04	207.99 ± 17.1	152.99 ± 11.82
C1qa	8	2.46 ± 0.31	5.11 ± 0.25	0.001 ± 0.0001	0.32 ± 0.03	306.37 ± 19.28	207.14 ± 11.93	2.06 ± 0.34	4.44 ± 0.41	0.0017 ± 0.0003	0.32 ± 0.05	262.64 ± 10.57	178.58 ± 15.6
C9	6	1.94 ± 0.19	3.97 ± 0.3	0.0008 ± 0.0001	0.33 ± 0.03	243.53 ± 6.87	163.28 ± 9.77	1.64 ± 0.17	4.94 ± 0.57	0.0014 ± 0.0002	0.26 ± 0.05	271.93 ± 16.78	204.03 ± 23.37
Cfb	5	1.74 ± 0.36	4.04 ± 0.26	0.0009 ± 0.0002	0.3 ± 0.06	279.62 ± 15.58	194.06 ± 11.46	1.67 ± 0.27	2.78 ± 0.47	0.0016 ± 0.0002	0.39 ± 0.06	211.34 ± 20.76	131.36 ± 20.05
Cp	11	3.66 ± 0.44	3.32 ± 0.63	0.0014 ± 0.0002	0.55 ± 0.07	270.83 ± 14.35	127.19 ± 22.76	1.72 ± 0.28	5.17 ± 0.28	0.0013 ± 0.0002	0.25 ± 0.04	269.72 ± 12.97	202.23 ± 13.34
Dnajb3	5	1.92 ± 0.13	4.38 ± 0.46	0.0009 ± 0.0001	0.31 ± 0.03	289.04 ± 37.39	202.45 ± 32.14	1.02 ± 0.08	4.39 ± 0.41	0.0009 ± 0.0001	0.19 ± 0.03	244.08 ± 21.9	198.48 ± 22.1
Dnase112	8	2.3 ± 0.6	5.54 ± 0.67	0.0009 ± 0.0002	0.28 ± 0.06	309.9 ± 37.19	219.43 ± 26.63	1.39 ± 0.24	5.94 ± 0.79	0.0011 ± 0.0002	0.21 ± 0.04	287.36 ± 20.23	231.65 ± 25.48
Epb4.114a	8	2.99 ± 0.6	1.7 ± 0.46	0.0013 ± 0.0002	0.62 ± 0.08	196.88 ± 26.77	70.94 ± 18.85	2.95 ± 0.82	3.11 ± 0.72	0.0026 ± 0.0007	0.48 ± 0.11	261.86 ± 21.02	133.68 ± 31.22
Far2	6	3.86 ± 0.94	3.04 ± 1.08	0.0017 ± 0.0004	0.58 ± 0.14	297.74 ± 25.03	128.3 ± 44.57	3.52 ± 2	5.42 ± 0.91	0.0029 ± 0.0016	0.32 ± 0.12	382.13 ± 52.59	237.01 ± 41.92
Gipc3	8	2.71 ± 0.44	3.12 ± 0.68	0.0009 ± 0.0001	0.49 ± 0.09	199.46 ± 15.26	107.26 ± 22.72	5.89 ± 4.35	5.31 ± 0.13	0.0046 ± 0.0035	0.3 ± 0.09	411.98 ± 178.23	182.75 ± 6.6
Hdac10	8	3.28 ± 0.41	2.87 ± 0.71	0.0012 ± 0.0002	0.56 ± 0.09	215.24 ± 18.18	98.85 ± 22.82	1.92 ± 0.23	5.8 ± 0.24	0.0014 ± 0.0002	0.24 ± 0.02	276.71 ± 21.25	207.3 ± 12.51
Hspb2	7	1.98 ± 0.4	4.44 ± 0.35	0.0008 ± 0.0002	0.3 ± 0.05	263.43 ± 15.6	181.41 ± 12.94	1.71 ± 0.37	3.31 ± 0.4	0.0014 ± 0.0003	0.34 ± 0.06	204.8 ± 20.42	133.59 ± 13.96
Htr1a	8	1.88 ± 0.46	3.77 ± 0.65	0.0007 ± 0.0002	0.35 ± 0.09	207.8 ± 14.28	139.1 ± 25.17	2.82 ± 1.73	5.87 ± 0.94	0.002 ± 0.0012	0.25 ± 0.11	313.53 ± 68.18	212.39 ± 35.06
Il12rb2	8	2.9 ± 0.58	2.19 ± 0.77	0.0013 ± 0.0003	0.6 ± 0.13	220.91 ± 9.87	89.85 ± 30.91	1.67 ± 0.45	4.06 ± 0.66	0.0015 ± 0.0005	0.32 ± 0.1	250.86 ± 23.63	174.35 ± 30.04
Lpar6	0	n.d.	n.d.	0 ± 0	n.d.	n.d.	n.d.	n.d.	n.d.	n.d.	n.d.	n.d.	n.d.
Parp8	8	2.32 ± 0.53	3.02 ± 0.59	0.001 ± 0.0002	0.44 ± 0.1	242.13 ± 19.5	137.73 ± 28	1.33 ± 0.22	3.17 ± 0.27	0.0012 ± 0.0002	0.3 ± 0.05	202.09 ± 8.8	142.71 ± 13.22
Pitx3	5	2.04 ± 0.42	2.32 ± 0.42	0.0013 ± 0.0003	0.47 ± 0.07	274.99 ± 31.5	143.63 ± 19.87	1.69 ± 0.16	3.56 ± 0.31	0.0021 ± 0.0002	0.33 ± 0.04	345.9 ± 44.04	238.89 ± 40.93
Pnmt	0	n.d.	n.d.	0 ± 0	n.d.	n.d.	n.d.	n.d.	n.d.	n.d.	n.d.	n.d.	n.d.
Rilp12	8	2.63 ± 0.61	4.19 ± 0.48	0.0009 ± 0.0002	0.37 ± 0.07	245.11 ± 19.79	151.66 ± 18.23	2.41 ± 0.37	2.8 ± 0.47	0.0017 ± 0.0003	0.47 ± 0.08	187.66 ± 11.2	102.23 ± 18.26

		40 mg/L						80 mg/L					
**Female**	n=	Amount of Drug Consumed	Amount of Water Cosnumed	Drug Weight	Preference (decimal %)	Total Drinking (g/kg bodyweight)	Water Drinking	Amount of Drug Consumed	Amount of Water Cosnumed	Drug Weight	Preference (decimal %)	Total Drinking (g/kg bodyweight)	Water Drinking
C57Bl6/6NJ	29	1.94 ± 0.28	5.74 ± 0.53	0.0036 ± 0.0005	0.27 ± 0.04	350.2 ± 25.92	261.36 ± 25.67	1.49 ± 0.28	4.07 ± 0.25	0.0054 ± 0.001	0.25 ± 0.03	248.3 ± 15.27	181.12 ± 10.66
Btg2	8	1.78 ± 0.3	5.22 ± 0.68	0.0032 ± 0.0005	0.28 ± 0.06	317.63 ± 24.34	237.41 ± 31.76	0.91 ± 0.12	3.41 ± 0.32	0.0033 ± 0.0005	0.22 ± 0.03	193.77 ± 12.07	152.36 ± 11.59
C1qa	8	1.94 ± 0.17	4.61 ± 0.51	0.0038 ± 0.0004	0.3 ± 0.02	320.08 ± 26.05	224.1 ± 19.93	0.88 ± 0.07	4.38 ± 0.38	0.0034 ± 0.0003	0.17 ± 0.02	250.68 ± 14.67	208.39 ± 14.12
C9	7	1.67 ± 0.56	4.97 ± 0.18	0.0033 ± 0.0012	0.22 ± 0.04	324.69 ± 42.12	240.99 ± 15.23	1.17 ± 0.19	5.24 ± 0.35	0.0046 ± 0.0009	0.18 ± 0.03	313.19 ± 25.39	255.16 ± 23.06
Cfb	5	1.25 ± 0.23	5.07 ± 0.41	0.0026 ± 0.0005	0.2 ± 0.04	328.09 ± 10.38	262.51 ± 19.61	0.68 ± 0.04	3.06 ± 0.15	0.0028 ± 0.0002	0.18 ± 0.01	194.5 ± 8.61	159.1 ± 8.07
Cp	11	1.49 ± 0.16	6.65 ± 0.35	0.0025 ± 0.0003	0.19 ± 0.02	344.12 ± 20.17	281.81 ± 20.44	1.28 ± 0.22	5.2 ± 0.42	0.0041 ± 0.0005	0.19 ± 0.03	265.39 ± 13.05	214.29 ± 12.95
Dnajb3	5	2.22 ± 0.76	4.74 ± 0.8	0.0044 ± 0.0019	0.32 ± 0.11	320.94 ± 22.02	210.61 ± 31.39	0.79 ± 0.05	3.56 ± 0.18	0.0029 ± 0.0003	0.18 ± 0.01	203.18 ± 22.89	166.61 ± 20.34
Dnase112	8	1.97 ± 0.41	4.6 ± 0.8	0.004 ± 0.0008	0.31 ± 0.06	332.26 ± 39.25	231.69 ± 35.69	1.21 ± 0.28	2.98 ± 0.46	0.005 ± 0.0012	0.31 ± 0.08	214.74 ± 15.83	151.9 ± 23.12
Epb4.114a	8	2.99 ± 1.35	6.88 ± 1.49	0.0061 ± 0.0029	0.26 ± 0.03	496.15 ± 150.17	344.76 ± 79.97	3.01 ± 1.75	4.89 ± 0.77	0.0123 ± 0.0074	0.27 ± 0.06	397.4 ± 124.34	243.66 ± 39.14
Far2	7	4.77 ± 1.2	4.54 ± 1.59	0.0088 ± 0.0022	0.55 ± 0.11	427.8 ± 94.6	208.01 ± 72.62	2.53 ± 0.97	5.39 ± 0.95	0.0099 ± 0.0041	0.28 ± 0.08	374.32 ± 67.44	250.31 ± 46.12
Gipc3	8	1.86 ± 0.42	3.88 ± 0.51	0.003 ± 0.0006	0.33 ± 0.08	238.11 ± 13.02	163.53 ± 23.05	2.32 ± 1.33	4.95 ± 0.36	0.0078 ± 0.0046	0.24 ± 0.06	300.11 ± 63.2	202.41 ± 13.1
Hdac10	7	1.47 ± 0.23	5.55 ± 0.33	0.0026 ± 0.0004	0.21 ± 0.04	310.99 ± 14.41	246.3 ± 18.55	1.1 ± 0.1	4.85 ± 0.26	0.0039 ± 0.0003	0.19 ± 0.01	262.41 ± 13.67	213.94 ± 12.58
Hspb2	8	1.57 ± 0.23	3.57 ± 0.63	0.0032 ± 0.0005	0.34 ± 0.05	250.19 ± 30.74	171.3 ± 27.75	0.93 ± 0.08	2.5 ± 0.37	0.0038 ± 0.0004	0.3 ± 0.05	167.1 ± 11.72	120.08 ± 15.42
Htr1a	11	2.67 ± 0.69	4.7 ± 0.44	0.0051 ± 0.0016	0.34 ± 0.06	335.38 ± 29.93	209.09 ± 19.08	2.76 ± 1.38	3.81 ± 0.48	0.0105 ± 0.0055	0.33 ± 0.05	310.22 ± 85.91	178.75 ± 27.24
Il12rb2	8	6.14 ± 1.58	4.96 ± 1.3	0.0128 ± 0.0034	0.52 ± 0.12	558.64 ± 93.88	237.56 ± 63.57	1.7 ± 0.34	3.99 ± 0.71	0.0071 ± 0.0017	0.34 ± 0.09	275.11 ± 26.53	186.78 ± 30.88
Lpar6	0	n.d.	n.d.	n.d.	n.d.	n.d.	n.d.	n.d.	n.d.	n.d.	n.d.	n.d.	n.d.
Parp8	8	2.83 ± 0.4	3.02 ± 0.73	0.0058 ± 0.0008	0.52 ± 0.09	297.09 ± 20.65	152.01 ± 36.21	0.96 ± 0.23	3.71 ± 0.69	0.0039 ± 0.0009	0.24 ± 0.07	241 ± 34	192.51 ± 37.89
Pitx3	5	2.25 ± 0.68	3.59 ± 0.63	0.0055 ± 0.0018	0.35 ± 0.13	318.9 ± 45.52	180.66 ± 53.97	1.15 ± 0.12	4.45 ± 0.44	0.0057 ± 0.0008	0.21 ± 0.03	337.47 ± 15.52	266.34 ± 16.61
Pnmt	0	n.d.	n.d.	n.d.	n.d.	n.d.	n.d.	n.d.	n.d.	n.d.	n.d.	n.d.	n.d.
Rilp12	8	1.38 ± 0.17	5.56 ± 0.24	0.0025 ± 0.0003	0.2 ± 0.02	316.83 ± 11.63	254.33 ± 8.63	0.9 ± 0.09	3.33 ± 0.15	0.0033 ± 0.0003	0.21 ± 0.01	193.36 ± 7.63	152.25 ± 5.41
**Male**													
C57Bl6/6NJ	31	2.3 ± 0.31	5.5 ± 0.45	0.0035 ± 0.0005	0.31 ± 0.04	295.57 ± 16.25	204.79 ± 17.54	1.7 ± 0.46	3.82 ± 0.24	0.0051 ± 0.0014	0.28 ± 0.03	204.9 ± 20.06	141.69 ± 8.74
Btg2	8	1.9 ± 0.42	4.9 ± 0.51	0.003 ± 0.0007	0.28 ± 0.06	263.31 ± 16.44	188.68 ± 18.67	0.93 ± 0.11	3.43 ± 0.39	0.0029 ± 0.0004	0.23 ± 0.04	167.49 ± 10.6	130.97 ± 13.66
C1qa	8	2.29 ± 0.32	6.29 ± 0.23	0.0038 ± 0.0006	0.26 ± 0.02	349.29 ± 26.57	255.25 ± 13.81	1.34 ± 0.22	3.45 ± 0.18	0.0043 ± 0.0007	0.27 ± 0.04	193.23 ± 9.03	138.93 ± 6.29
C9	6	1.31 ± 0.07	5.15 ± 0.35	0.0022 ± 0.0001	0.2 ± 0.01	266.78 ± 12.61	212.64 ± 12.97	1.27 ± 0.2	5.74 ± 0.26	0.0042 ± 0.0006	0.18 ± 0.02	289.23 ± 9.77	237.17 ± 10.54
Cfb	5	1.76 ± 0.46	4.86 ± 0.69	0.0034 ± 0.0009	0.28 ± 0.08	317.52 ± 5.86	231.64 ± 28.95	0.89 ± 0.23	2.74 ± 0.53	0.0035 ± 0.0011	0.28 ± 0.11	173.57 ± 11.97	129.33 ± 23.99
Cp	11	2.26 ± 0.33	5.61 ± 0.77	0.0035 ± 0.0005	0.32 ± 0.06	303.8 ± 25.3	215.23 ± 27.53	1.31 ± 0.14	4.85 ± 0.43	0.0042 ± 0.0005	0.22 ± 0.02	242.29 ± 21.97	189.99 ± 18.81
Dnajb3	5	1.73 ± 0.1	6.1 ± 0.37	0.0031 ± 0.0003	0.22 ± 0.02	355.9 ± 34.96	278.03 ± 30.94	1.04 ± 0.02	3.24 ± 0.52	0.0038 ± 0.0003	0.27 ± 0.05	190.92 ± 24.34	143.65 ± 23.92
Dnase112	8	2.25 ± 0.96	5.76 ± 0.45	0.0036 ± 0.0016	0.25 ± 0.07	318.94 ± 26.45	227.95 ± 18.02	2.83 ± 1.61	3.51 ± 0.54	0.0091 ± 0.0053	0.34 ± 0.08	249.38 ± 60.2	135.25 ± 16.96
Epb4.114a	8	2.05 ± 0.37	4.01 ± 0.73	0.0035 ± 0.0007	0.37 ± 0.09	258.45 ± 20.63	169.86 ± 29.68	1.44 ± 0.32	2.85 ± 0.2	0.0052 ± 0.0013	0.32 ± 0.05	185.78 ± 17.95	120.92 ± 5.29
Far2	6	2.04 ± 0.85	4.59 ± 1.07	0.0036 ± 0.0015	0.33 ± 0.14	286.12 ± 20.47	196.71 ± 45.98	1.22 ± 0.21	4.71 ± 0.48	0.0042 ± 0.0007	0.21 ± 0.05	257.02 ± 18.5	204.44 ± 23.65
Gipc3	8	2.06 ± 0.47	4.6 ± 0.56	0.003 ± 0.0008	0.32 ± 0.08	229.38 ± 9.27	154.48 ± 17.4	1.5 ± 0.21	5.1 ± 0.21	0.0043 ± 0.0007	0.22 ± 0.03	228.82 ± 17.43	175.47 ± 9.58
Hdac10	8	1.54 ± 0.16	4.83 ± 0.22	0.0022 ± 0.0003	0.24 ± 0.02	226.56 ± 12.71	171.35 ± 7.77	1.67 ± 0.21	5.56 ± 0.3	0.0048 ± 0.0007	0.23 ± 0.02	258.18 ± 17.39	198.13 ± 12.63
Hspb2	7	2.31 ± 0.4	5.02 ± 0.78	0.0039 ± 0.0007	0.33 ± 0.08	298.77 ± 30.01	202.5 ± 29.87	1.3 ± 0.26	2.61 ± 0.37	0.0043 ± 0.0008	0.34 ± 0.06	159.54 ± 15.06	105.96 ± 14.56
Htr1a	8	1.57 ± 0.17	4.3 ± 0.63	0.0023 ± 0.0003	0.29 ± 0.05	209.38 ± 14.57	151.76 ± 19.59	1.25 ± 0.24	3.85 ± 0.59	0.0039 ± 0.001	0.27 ± 0.07	183.89 ± 16.79	135.64 ± 18.77
Il12rb2	8	4.63 ± 1.5	5.42 ± 1.41	0.0085 ± 0.0028	0.46 ± 0.11	457.06 ± 105.33	245.53 ± 71.63	2.33 ± 1.05	2.86 ± 0.58	0.0085 ± 0.0041	0.4 ± 0.12	227.43 ± 35.25	120.75 ± 23.69
Lpar6	0	n.d.	n.d.	n.d.	n.d.	n.d.	n.d.	n.d.	n.d.	n.d.	n.d.	n.d.	n.d.
Parp8	8	1.91 ± 0.36	3.38 ± 0.45	0.0034 ± 0.0006	0.37 ± 0.07	238.61 ± 17.25	152.86 ± 22.51	0.85 ± 0.18	4.05 ± 0.32	0.003 ± 0.0007	0.18 ± 0.04	219.26 ± 9.82	181.59 ± 13.62
Pitx3	5	2.1 ± 0.31	3.8 ± 0.45	0.0052 ± 0.0004	0.36 ± 0.04	372.67 ± 25.02	243.87 ± 33.56	0.88 ± 0.11	3.7 ± 0.12	0.0045 ± 0.0005	0.19 ± 0.02	295.43 ± 21.97	239.73 ± 20.39
Pnmt	0	n.d.	n.d.	n.d.	n.d.	n.d.	n.d.	n.d.	n.d.	n.d.	n.d.	n.d.	n.d.
Rilp12	8	1.74 ± 0.39	5.39 ± 0.69	0.0024 ± 0.0005	0.26 ± 0.08	256.6 ± 14.01	195.66 ± 24.75	1.18 ± 0.14	2.78 ± 0.32	0.0034 ± 0.0004	0.31 ± 0.05	141.18 ± 8.32	98.73 ± 10.58

Supplemental Table 11

Nicotine		10 mg/L						20 mg/L					
**Female**	n=	Amount of Drug Consumed	Amount of Water Cosnumed	Drug Weight	Preference (decimal %)	Total Drinking (g/kg bodyweight)	Water Drinking	Amount of Drug Consumed	Amount of Water Cosnumed	Drug Weight	Preference (decimal %)	Total Drinking (g/kg bodyweight)	Water Drinking
C57BL/6NJ	30	4.44 ± 0.37	4.68 ± 0.42	0.002 ± 0.0002	0.49 ± 0.03	425.63 ± 25	221.29 ± 21.84	5.04 ± 0.45	4.69 ± 0.44	0.0047 ± 0.0005	0.52 ± 0.03	452.02 ± 34.67	216.13 ± 21.22
Btg2	8	6.32 ± 1	5.71 ± 0.63	0.003 ± 0.0005	0.51 ± 0.04	575.78 ± 78.81	272.08 ± 36.54	6.4 ± 1.17	6.33 ± 1.53	0.006 ± 0.0012	0.51 ± 0.08	602.34 ± 88.84	302.15 ± 82.5
C1qa	8	4.94 ± 0.7	4.62 ± 0.72	0.0022 ± 0.0003	0.52 ± 0.06	432.83 ± 35.96	207.92 ± 30.34	5.63 ± 0.94	4.77 ± 0.85	0.0051 ± 0.0008	0.54 ± 0.07	470.94 ± 58.81	216.39 ± 37.8
C9	8	3.5 ± 0.5	4.04 ± 0.76	0.0057 ± 0.004	0.48 ± 0.07	907.65 ± 539.03	337.9 ± 139.36	5.41 ± 1.18	4.26 ± 0.75	0.0273 ± 0.0229	0.54 ± 0.09	2147.55 ± 1738	780.78 ± 595.33
Cfb	2	4.35 ± 2.41	3.63 ± 1.74	0.0022 ± 0.0012	0.53 ± 0.03	394.33 ± 213.89	179.2 ± 90.09	3.89 ± 2.06	3.45 ± 1.56	0.0038 ± 0.0021	0.52 ± 0.03	362.09 ± 186.61	169.93 ± 80.82
Cp	5	6.28 ± 1.02	5.48 ± 1	0.0028 ± 0.0004	0.53 ± 0.08	525.18 ± 18.41	247.08 ± 47.51	5.26 ± 0.51	3.67 ± 0.49	0.0048 ± 0.0006	0.59 ± 0.05	402.26 ± 35.61	164.13 ± 22.27
Dnajb3	7	5.15 ± 0.99	5.24 ± 1.12	0.0027 ± 0.0005	0.5 ± 0.09	557.89 ± 58.93	287.94 ± 70.47	6.13 ± 1.29	6.68 ± 1.2	0.0065 ± 0.0013	0.48 ± 0.08	689.13 ± 82.13	365.67 ± 76.81
Dnase112	5	5.09 ± 0.31	5.32 ± 1	0.0025 ± 0.0001	0.51 ± 0.07	515.94 ± 52.7	267.23 ± 51.43	4.36 ± 1.82	3.28 ± 0.74	0.004 ± 0.0015	0.49 ± 0.1	354.53 ± 90.36	153.93 ± 27.62
Epb4.114a	8	5.61 ± 0.63	4.34 ± 0.66	0.0027 ± 0.0003	0.56 ± 0.05	470.65 ± 35.77	203.7 ± 27.74	6.47 ± 1.28	4.14 ± 0.8	0.0062 ± 0.0013	0.58 ± 0.08	510.14 ± 71.66	201.13 ± 41.73
Far2	8	5.02 ± 0.86	4.69 ± 0.68	0.0023 ± 0.0004	0.51 ± 0.06	433.78 ± 38.16	207.95 ± 28.39	4.12 ± 0.65	3.45 ± 0.65	0.0037 ± 0.0006	0.57 ± 0.05	336.79 ± 49.23	153.02 ± 28.18
Gipc3	8	5.71 ± 0.59	5.12 ± 0.85	0.0025 ± 0.0003	0.54 ± 0.05	479.44 ± 48.91	228.68 ± 41.18	6.32 ± 1.2	4.4 ± 0.98	0.0056 ± 0.0011	0.58 ± 0.09	473.49 ± 35.7	193.59 ± 43.47
Hdac10	7	5.61 ± 0.88	5.3 ± 0.63	0.0029 ± 0.0004	0.51 ± 0.04	558.12 ± 57.89	271.6 ± 32.5	7.31 ± 1.13	6.31 ± 1.13	0.0074 ± 0.0011	0.54 ± 0.06	687.29 ± 68.95	316.19 ± 52.64
Hspb2	8	7.25 ± 0.67	4.21 ± 0.82	0.0036 ± 0.0004	0.64 ± 0.05	569.23 ± 57.98	208.31 ± 43.08	4.53 ± 0.53	5.65 ± 1.7	0.0045 ± 0.0007	0.5 ± 0.07	513.5 ± 114.49	286.82 ± 95.84
Htr1a	8	4.43 ± 0.85	4.16 ± 0.74	0.0022 ± 0.0004	0.5 ± 0.05	415.79 ± 63.99	200.03 ± 36.54	5.29 ± 0.7	4.89 ± 0.3	0.005 ± 0.0007	0.51 ± 0.05	484.84 ± 29.27	233.34 ± 14.51
Il12rb2	8	6.72 ± 0.75	4 ± 0.82	0.0032 ± 0.0004	0.64 ± 0.05	515.57 ± 46.09	191.22 ± 38.78	6.8 ± 1.51	3.89 ± 0.74	0.0067 ± 0.0016	0.59 ± 0.07	522.32 ± 85.84	186.92 ± 36.37
Lpar6	0	n.d.	n.d.	n.d.	n.d.	n.d.	n.d.	n.d.	n.d.	n.d.	n.d.	n.d.	n.d.
Parp8	9	4.55 ± 0.57	3.33 ± 0.54	0.0025 ± 0.0003	0.58 ± 0.05	423.74 ± 40.04	177.48 ± 28.17	4.15 ± 0.68	3.27 ± 0.62	0.0043 ± 0.0007	0.56 ± 0.05	390.42 ± 53.81	174.05 ± 33.79
Pitx3	8	4.62 ± 0.62	3.24 ± 0.44	0.0026 ± 0.0004	0.58 ± 0.06	445.66 ± 35.91	184.53 ± 27.41	4.92 ± 0.43	5.51 ± 0.85	0.0058 ± 0.0006	0.49 ± 0.03	617.83 ± 84.12	329.19 ± 61.49
Pnmt	0	n.d.	n.d.	n.d.	n.d.	n.d.	n.d.	n.d.	n.d.	n.d.	n.d.	n.d.	n.d.
Rilp12	8	4.55 ± 0.61	4.68 ± 0.5	0.0019 ± 0.0002	0.49 ± 0.05	398.17 ± 15.68	204.48 ± 22.29	4.46 ± 0.4	3.84 ± 0.83	0.0039 ± 0.0004	0.56 ± 0.07	361.31 ± 24.1	167.19 ± 38.52
**Male**													
C57BL/6NJ	31	4.94 ± 0.38	4.73 ± 0.34	0.0019 ± 0.0001	0.51 ± 0.03	369.44 ± 13.68	180.64 ± 13.27	4.93 ± 0.41	6.97 ± 2.2	0.0039 ± 0.0004	0.5 ± 0.04	446.72 ± 70.9	253.22 ± 71.41
Btg2	8	5.74 ± 0.84	5.26 ± 0.71	0.0023 ± 0.0004	0.52 ± 0.07	447.51 ± 35.52	212.57 ± 26.9	5.03 ± 0.9	7.09 ± 1.35	0.0041 ± 0.0008	0.43 ± 0.08	495.99 ± 52.92	289.37 ± 55.76
C1qa	8	4.93 ± 0.47	4.47 ± 0.77	0.0019 ± 0.0002	0.54 ± 0.06	361.02 ± 36.04	171.17 ± 30.2	4.74 ± 0.83	3.8 ± 0.32	0.0037 ± 0.0007	0.53 ± 0.05	329.07 ± 32.04	145.84 ± 12.67
C9	8	3.78 ± 0.7	6.03 ± 0.88	0.0016 ± 0.0003	0.39 ± 0.07	421.12 ± 48.35	259.81 ± 39.69	3.74 ± 0.49	5.03 ± 1.09	0.0032 ± 0.0005	0.46 ± 0.07	372.04 ± 39.54	211.49 ± 43.44
Cfb	2	4.47 ± 0.28	2.38 ± 1.26	0.0019 ± 0.0002	0.67 ± 0.14	281.31 ± 20.52	94.96 ± 45.14	3.99 ± 2.17	5.39 ± 0.25	0.0034 ± 0.002	0.39 ± 0.13	396.88 ± 128.38	224.65 ± 26.46
Cp	4	3.59 ± 0.69	3.85 ± 0.65	0.0016 ± 0.0001	0.47 ± 0.02	321.7 ± 24.42	165 ± 12.11	3.05 ± 0.69	4.39 ± 0.94	0.0027 ± 0.0004	0.42 ± 0.04	327.59 ± 20.99	194.02 ± 12.94
Dnajb3	7	4.56 ± 0.37	5.12 ± 0.95	0.0019 ± 0.0002	0.49 ± 0.06	402.36 ± 40.94	213.08 ± 39.19	5.46 ± 1.01	5.6 ± 1.06	0.0046 ± 0.0009	0.49 ± 0.07	466.98 ± 71.79	236.7 ± 49.74
Dnase112	5	5.41 ± 0.85	4.94 ± 0.65	0.0022 ± 0.0003	0.52 ± 0.07	440.18 ± 46.39	221.46 ± 52.01	2.83 ± 0.89	3.37 ± 1.06	0.0021 ± 0.0005	0.46 ± 0.09	240.31 ± 34.01	134.07 ± 33.91
Epb4.114a	8	4.49 ± 0.83	5.11 ± 0.84	0.0018 ± 0.0003	0.46 ± 0.07	386.55 ± 38.72	210.43 ± 35.74	5.57 ± 1.12	5 ± 1.2	0.0047 ± 0.0011	0.52 ± 0.08	445.56 ± 72.51	212.29 ± 60.24
Far2	8	4.89 ± 1.03	4.47 ± 0.51	0.0018 ± 0.0004	0.5 ± 0.07	351.48 ± 31.54	167.42 ± 19.24	5.58 ± 0.52	3.51 ± 0.76	0.0042 ± 0.0004	0.63 ± 0.07	341.51 ± 28.95	133.53 ± 29.98
Gipc3	8	4.35 ± 0.84	4.4 ± 0.66	0.0015 ± 0.0003	0.49 ± 0.08	305.01 ± 16.42	152.16 ± 21.04	4.66 ± 0.78	4.69 ± 0.95	0.0033 ± 0.0006	0.51 ± 0.08	325.78 ± 30.92	161.49 ± 30.13
Hdac10	8	4.68 ± 0.91	4.1 ± 0.72	0.0018 ± 0.0004	0.53 ± 0.05	344.93 ± 46.54	163.89 ± 31.89	3.91 ± 0.74	5.72 ± 1.54	0.0029 ± 0.0006	0.45 ± 0.1	370.86 ± 64.89	224.89 ± 64.75
Hspb2	6	2.94 ± 0.44	4.59 ± 1.28	0.0013 ± 0.0003	0.44 ± 0.08	334.42 ± 81.11	204.8 ± 66.29	4.18 ± 0.99	3.44 ± 0.71	0.0038 ± 0.0011	0.54 ± 0.09	337.56 ± 62.88	147.7 ± 30.26
Htr1a	8	4.29 ± 0.28	3.07 ± 0.35	0.0018 ± 0.0002	0.59 ± 0.03	300.81 ± 29.85	124.89 ± 16.27	4.87 ± 0.36	3.13 ± 0.53	0.0039 ± 0.0003	0.62 ± 0.05	324.39 ± 22.95	128.96 ± 23.75
Il12rb2	8	3.86 ± 0.57	4.17 ± 0.91	0.0016 ± 0.0003	0.49 ± 0.06	328.64 ± 48.75	171.2 ± 41.69	5.07 ± 0.89	4.55 ± 1.08	0.0041 ± 0.0008	0.53 ± 0.07	395.09 ± 70.21	189.43 ± 49.13
Lpar6	0	n.d.	n.d.	n.d.	n.d.	n.d.	n.d.	n.d.	n.d.	n.d.	n.d.	n.d.	n.d.
Parp8	7	6.04 ± 0.88	2.59 ± 0.49	0.0029 ± 0.0005	0.68 ± 0.08	407.99 ± 34.2	122.7 ± 27.6	5.6 ± 1.32	5.3 ± 0.88	0.0057 ± 0.0013	0.49 ± 0.06	532.67 ± 90.68	247.6 ± 52.36
Pitx3	8	3.77 ± 0.67	3.99 ± 0.28	0.0019 ± 0.0003	0.47 ± 0.05	394.25 ± 32.43	204.05 ± 18.16	4.51 ± 0.8	4.04 ± 0.76	0.0046 ± 0.0009	0.52 ± 0.09	432.83 ± 27.75	203.88 ± 39.49
Pnmt	0	n.d.	n.d.	n.d.	n.d.	n.d.	n.d.	n.d.	n.d.	n.d.	n.d.	n.d.	n.d.
Rilp12	7	3.68 ± 0.45	3.16 ± 0.59	0.0012 ± 0.0001	0.55 ± 0.07	233.88 ± 16.35	110.67 ± 24.66	4.31 ± 0.37	3.32 ± 0.5	0.003 ± 0.0003	0.57 ± 0.03	264.2 ± 32.97	114.96 ± 21.19

		40 mg/L						80 mg/L					
**Female**	n=	Amount of Drug Consumed	Amount of Water Cosnumed	Drug Weight	Preference (decimal %)	Total Drinking (g/kg bodyweight)	Water Drinking	Amount of Drug Consumed	Amount of Water Cosnumed	Drug Weight	Preference (decimal %)	Total Drinking (g/kg bodyweight)	Water Drinking
C57BL/6NJ	30	4.43 ± 0.47	7.2 ± 2.4	0.0082 ± 0.0009	0.47 ± 0.04	524.11 ± 90.53	319.71 ± 96.89	3.42 ± 0.38	4.54 ± 0.42	0.0126 ± 0.0014	0.43 ± 0.04	368.48 ± 25.65	211.47 ± 21.8
Btg2	8	4.94 ± 1.18	3.66 ± 0.92	0.0094 ± 0.0024	0.57 ± 0.07	406.57 ± 78.77	171.47 ± 46.27	4.52 ± 1.45	5.29 ± 1.1	0.0178 ± 0.006	0.43 ± 0.1	462.58 ± 62.94	240.11 ± 47.44
C1qa	8	5.51 ± 0.76	4.74 ± 0.74	0.0101 ± 0.0014	0.55 ± 0.03	464.63 ± 59.17	212.95 ± 31.29	3.35 ± 0.56	2.99 ± 0.42	0.0121 ± 0.0019	0.53 ± 0.04	286.94 ± 32.11	135.36 ± 18.43
C9	8	5.8 ± 1.36	3.77 ± 1.05	0.0269 ± 0.0152	0.59 ± 0.09	953.74 ± 555.4	303.75 ± 122.51	4.6 ± 1.34	3.75 ± 0.87	0.0642 ± 0.0463	0.53 ± 0.1	1064.23 ± 641.8	261.46 ± 73.93
Cfb	2	5.23 ± 0.3	4.05 ± 1.88	0.0102 ± 0.0009	0.59 ± 0.11	454.3 ± 119.63	199.45 ± 97.14	3.44 ± 1.2	2.51 ± 0.36	0.0135 ± 0.0051	0.56 ± 0.05	291.51 ± 84.85	122.58 ± 21.45
Cp	5	4.89 ± 0.25	5.82 ± 0.86	0.0088 ± 0.0007	0.47 ± 0.05	480.5 ± 45.89	260.11 ± 39.99	2.8 ± 0.49	3.99 ± 0.26	0.0099 ± 0.0014	0.4 ± 0.06	303.33 ± 10.52	180.03 ± 15.94
Dnajb3	7	5.09 ± 0.84	4.98 ± 1.24	0.0111 ± 0.002	0.54 ± 0.09	537.07 ± 67.73	259.06 ± 58.67	2.7 ± 0.75	5.46 ± 0.94	0.011 ± 0.0026	0.34 ± 0.06	432.29 ± 54.57	295.4 ± 60.22
Dnase112	5	5.46 ± 1.23	5.8 ± 2.32	0.0105 ± 0.0022	0.52 ± 0.12	564.01 ± 121.8	301.25 ± 134.73	3.61 ± 2.33	2.86 ± 0.91	0.0133 ± 0.0092	0.5 ± 0.25	287.44 ± 86.06	121.51 ± 28.99
Epb4.114a	8	6.01 ± 1.04	6.3 ± 0.87	0.0113 ± 0.0017	0.48 ± 0.06	588.15 ± 58.11	306.54 ± 45.97	3.68 ± 1.04	6.25 ± 1.4	0.0142 ± 0.0047	0.37 ± 0.06	474.65 ± 130.68	296.99 ± 78.15
Far2	8	5.43 ± 0.91	7.41 ± 1.07	0.0097 ± 0.0016	0.43 ± 0.06	576.91 ± 42.01	333.41 ± 49.4	3.34 ± 0.63	4.24 ± 0.55	0.0119 ± 0.0022	0.44 ± 0.06	336.93 ± 27.67	188.62 ± 24.23
Gipc3	8	4.37 ± 0.57	3.68 ± 0.7	0.0077 ± 0.0011	0.56 ± 0.07	354.84 ± 35.05	161.49 ± 31.08	4.03 ± 0.55	3.89 ± 0.71	0.0143 ± 0.002	0.52 ± 0.06	347.17 ± 41.08	168.76 ± 30.73
Hdac10	7	5.14 ± 0.65	4.46 ± 0.83	0.0105 ± 0.0014	0.55 ± 0.07	489.89 ± 36	227.99 ± 43.73	3.15 ± 0.56	3.66 ± 0.76	0.0126 ± 0.0022	0.48 ± 0.09	347.32 ± 27.22	189.3 ± 44.14
Hspb2	8	4.11 ± 0.49	3.81 ± 1.02	0.0083 ± 0.0012	0.55 ± 0.08	397.34 ± 46.48	184.46 ± 47.87	3.37 ± 0.69	3.8 ± 0.56	0.0135 ± 0.0029	0.46 ± 0.05	359.03 ± 58.24	190.8 ± 35.84
Htr1a	8	2.91 ± 0.59	3.66 ± 0.52	0.0056 ± 0.0011	0.43 ± 0.08	316.8 ± 25.29	177.84 ± 26.96	2.53 ± 0.4	3.94 ± 0.58	0.0096 ± 0.0015	0.4 ± 0.07	308.55 ± 19.7	188.33 ± 25.52
Il12rb2	8	3.2 ± 0.74	4.89 ± 0.87	0.0061 ± 0.0014	0.4 ± 0.07	391.66 ± 57.63	239.06 ± 45.51	3.67 ± 1.52	5.24 ± 0.72	0.0137 ± 0.0053	0.36 ± 0.08	422.8 ± 84.39	252.15 ± 34.35
Lpar6	0	n.d.	n.d.	n.d.	n.d.	n.d.	n.d.	n.d.	n.d.	n.d.	n.d.	n.d.	n.d.
Parp8	9	3.99 ± 0.77	1.94 ± 0.23	0.0083 ± 0.0016	0.64 ± 0.05	308.78 ± 34.88	100.9 ± 11.41	3.03 ± 0.85	3.35 ± 0.56	0.0126 ± 0.0034	0.45 ± 0.09	333.03 ± 35.13	175.82 ± 30.62
Pitx3	8	5.28 ± 0.83	3.05 ± 0.64	0.0124 ± 0.0021	0.63 ± 0.07	488.08 ± 52.4	178.92 ± 36.17	3.19 ± 0.31	2.79 ± 0.29	0.0148 ± 0.0013	0.53 ± 0.04	347.67 ± 17.42	162.78 ± 17.03
Pnmt	0	n.d.	n.d.	n.d.	n.d.	n.d.	n.d.	n.d.	n.d.	n.d.	n.d.	n.d.	n.d.
Rilp12	8	4.49 ± 0.55	4.26 ± 0.39	0.0078 ± 0.0009	0.51 ± 0.04	378.28 ± 23.6	184.18 ± 17.26	3.04 ± 0.37	2.47 ± 0.34	0.0107 ± 0.0014	0.55 ± 0.06	239.27 ± 14.29	104.98 ± 11.78
**Male**													
C57BL/6NJ	31	5.37 ± 0.47	6.87 ± 2.31	0.0081 ± 0.0007	0.54 ± 0.04	454.45 ± 71.19	251.13 ± 75.56	3.6 ± 0.36	4.02 ± 0.44	0.0108 ± 0.0011	0.48 ± 0.05	290.3 ± 15.72	155.92 ± 17.62
Btg2	8	4.61 ± 0.8	4.68 ± 1.22	0.0076 ± 0.0014	0.53 ± 0.1	378.49 ± 33.95	188.35 ± 48.03	4.09 ± 0.61	3.78 ± 1.05	0.0132 ± 0.0019	0.55 ± 0.09	321.69 ± 33.21	156.14 ± 44.35
C1qa	8	6.57 ± 1.07	5.39 ± 1.03	0.0101 ± 0.0017	0.55 ± 0.06	460.64 ± 53.64	208.53 ± 41.99	2.1 ± 0.42	5.1 ± 0.63	0.0063 ± 0.0012	0.3 ± 0.06	276.28 ± 22.23	196.93 ± 25.89
C9	8	3.07 ± 0.53	4.81 ± 1.03	0.0051 ± 0.0008	0.42 ± 0.06	329.02 ± 43.91	202.13 ± 42.98	2.31 ± 0.59	3.65 ± 1.04	0.0077 ± 0.0018	0.45 ± 0.12	253.69 ± 27.17	156.86 ± 43.58
Cfb	2	7.81 ± 0.34	6.74 ± 4.14	0.013 ± 0.0015	0.59 ± 0.16	617.55 ± 228.9	292.06 ± 191.71	2.74 ± 0.06	3.69 ± 1.49	0.0091 ± 0.0009	0.45 ± 0.1	271.56 ± 83.22	157.65 ± 72.55
Cp	4	6.27 ± 1.34	4.56 ± 1.24	0.0093 ± 0.0021	0.58 ± 0.11	399.37 ± 29.1	166.27 ± 43.85	3 ± 0.61	2.08 ± 0.5	0.0106 ± 0.0007	0.59 ± 0.03	222.7 ± 21.34	90.75 ± 14.36
Dnajb3	7	3.69 ± 0.59	4.06 ± 0.62	0.0063 ± 0.0012	0.48 ± 0.07	322.89 ± 21.37	166.22 ± 24.74	2.74 ± 0.62	3.56 ± 0.49	0.0093 ± 0.0023	0.43 ± 0.08	263.35 ± 23.66	146.79 ± 18.86
Dnase112	5	4.46 ± 0.93	5.57 ± 0.42	0.007 ± 0.001	0.43 ± 0.07	416.58 ± 20.04	242.43 ± 38.26	3.2 ± 0.51	3.16 ± 0.53	0.0083 ± 0.0009	0.5 ± 0.08	207.93 ± 10.86	104.19 ± 22.36
Epb4.114a	8	4.89 ± 0.62	4.68 ± 0.92	0.0078 ± 0.0009	0.52 ± 0.04	388.92 ± 49.38	193.12 ± 38.25	3.29 ± 0.76	4.39 ± 1.18	0.0112 ± 0.0024	0.45 ± 0.12	336.42 ± 49.23	196.45 ± 50.56
Far2	8	4.56 ± 1.12	8.03 ± 1.32	0.0067 ± 0.0016	0.36 ± 0.07	471.48 ± 48.03	304.23 ± 51.97	3.66 ± 0.53	3.22 ± 0.37	0.011 ± 0.0015	0.53 ± 0.05	257.21 ± 21.91	120.09 ± 12.87
Gipc3	8	2.89 ± 0.53	5.2 ± 0.83	0.004 ± 0.0007	0.37 ± 0.07	282.62 ± 25.45	181.77 ± 29.19	2.69 ± 0.56	4.05 ± 0.48	0.0074 ± 0.0015	0.39 ± 0.06	235.76 ± 25.68	143.3 ± 18.69
Hdac10	8	3.44 ± 1.01	4.14 ± 0.92	0.0054 ± 0.0017	0.43 ± 0.11	290.85 ± 33.51	154.84 ± 36.79	2.54 ± 0.46	4.09 ± 0.7	0.0078 ± 0.0016	0.4 ± 0.07	254.48 ± 36.84	157.42 ± 29.81
Hspb2	6	3.09 ± 0.26	2.89 ± 0.92	0.0054 ± 0.0007	0.57 ± 0.09	257.7 ± 39.93	122.91 ± 38.32	2.96 ± 1.13	3.99 ± 1.03	0.0103 ± 0.0039	0.4 ± 0.11	299.95 ± 55.02	170.69 ± 44.42
Htr1a	8	3.11 ± 0.55	2.86 ± 0.47	0.0049 ± 0.0007	0.52 ± 0.06	237.58 ± 19.94	116.11 ± 20.01	2.31 ± 0.31	3.33 ± 0.64	0.0073 ± 0.0009	0.44 ± 0.07	225.76 ± 21.28	134.17 ± 25.84
Il12rb2	8	4.09 ± 1.34	4.51 ± 1.07	0.0068 ± 0.0025	0.48 ± 0.1	376.26 ± 73.32	186.21 ± 47.59	3.7 ± 0.97	3.98 ± 0.59	0.0123 ± 0.0036	0.45 ± 0.08	314.62 ± 46.83	160.83 ± 26.69
Lpar6	0	n.d.	n.d.	n.d.	n.d.	n.d.	n.d.	0 ± 0	0 ± 0	0 ± 0	0 ± 0	0 ± 0	0 ± 0
Parp8	7	4.34 ± 0.88	5.08 ± 1.46	0.0088 ± 0.0018	0.49 ± 0.1	482.27 ± 84.24	261.74 ± 79.75	4.05 ± 0.67	3.98 ± 0.96	0.0137 ± 0.002	0.52 ± 0.08	374.97 ± 52.87	203.54 ± 54.4
Pitx3	8	4.22 ± 0.53	3.73 ± 0.59	0.0085 ± 0.001	0.54 ± 0.05	401.78 ± 40.66	189.26 ± 29.51	3.57 ± 0.33	2.63 ± 0.4	0.0144 ± 0.0013	0.58 ± 0.05	313.83 ± 23.12	133.46 ± 22.31
Pnmt	0	n.d.	n.d.	n.d.	n.d.	n.d.	n.d.	0 ± 0	0 ± 0	0 ± 0	0 ± 0	0 ± 0	0 ± 0
Rilp12	7	5.34 ± 0.82	3.51 ± 0.6	0.0074 ± 0.0012	0.59 ± 0.08	305.9 ± 35.96	121.92 ± 23.39	2.13 ± 0.32	3.41 ± 0.58	0.0058 ± 0.0009	0.41 ± 0.08	189.89 ± 21.57	117.45 ± 22.4

Supplemental Table 12

Strain	Phenotype	Dose	Dose:Sex	Sex	Strain	Relative to Control	Dose	Dose:Sex	Sex	Strain
Btg2<tm1b(KOMP)Mbp/2J	DrugWeightEthanol	0.000	0.007	0.000	0.006	↑↓	169.24(3,265)	4.18(3,265)	28.47(1,89)	10.03(1,89)
Btg2<tm1b(KOMP)Mbp/2J	DrugWeightMeth	0.000	0.988	0.936	0.556		18.06(3,213)	0.07(3,213)	0.29(1,71)	1.91(1,71)
Btg2<tm1b(KOMP)Mbp/2J	DrugWeightNicotine	0.000	0.718	0.945	0.991		15.75(3,198)	0.72(3,198)	0.06(1,66)	0.2(1,66)
Btg2<tm1b(KOMP)Mbp/2J	PreferenceEthanol	0.000	0.700	0.001	0.005	↑	12.54(3,263)	1.39(3,263)	14.93(1,89)	10.49(1,89)
Btg2<tm1b(KOMP)Mbp/2J	PreferenceMeth	0.000	0.042	0.820	0.506		27.96(3,204)	3.23(3,204)	0.12(1,68)	1.27(1,68)
Btg2<tm1b(KOMP)Mbp/2J	PreferenceNicotine	1.000	1.000	1.000	1.000		1	1	1	1
Btg2<tm1b(KOMP)Mbp/2J	Total.DrinkingEthanol	0.000	0.905	0.000	0.304		18.62(3,263)	0.25(3,263)	34.58(1,89)	1.25(1,89)
Btg2<tm1b(KOMP)Mbp/2J	Total.DrinkingMeth	0.000	0.296	0.000	0.108		18.95(3,207)	1.45(3,207)	20.17(1,69)	4.31(1,69)
Btg2<tm1b(KOMP)Mbp/2J	Total.DrinkingNicotine	0.640	0.949	0.609	0.921		0.62(3,198)	0.12(3,198)	0.52(1,66)	1.31(1,66)
C1qa<tm1b(EUCOMM)Wtsi>/3J	DrugWeightEthanol	0.000	0.002	0.000	0.015	↑	190.75(3,265)	6.6(3,265)	36.84(1,89)	7.42(1,89)
C1qa<tm1b(EUCOMM)Wtsi>/3J	DrugWeightMeth	0.000	0.988	0.936	0.700		19.5(3,213)	0.07(3,213)	0.08(1,71)	0.21(1,71)
C1qa<tm1b(EUCOMM)Wtsi>/3J	DrugWeightNicotine	0.000	0.718	0.945	0.991		11.63(3,201)	2.02(3,201)	0.03(1,67)	0.02(1,67)
C1qa<tm1b(EUCOMM)Wtsi>/3J	PreferenceEthanol	0.000	0.763	0.001	0.396		13.33(3,262)	0.75(3,262)	13.98(1,89)	0.94(1,89)
C1qa<tm1b(EUCOMM)Wtsi>/3J	PreferenceMeth	0.000	0.024	0.820	0.562		27.54(3,204)	4.72(3,204)	0.15(1,68)	0.77(1,68)
C1qa<tm1b(EUCOMM)Wtsi>/3J	PreferenceNicotine	1.000	1.000	1.000	1.000		1	1	1	1
C1qa<tm1b(EUCOMM)Wtsi>/3J	Total.DrinkingEthanol	0.000	0.798	0.000	0.000	↑	21.07(3,262)	0.71(3,262)	42.99(1,89)	20.68(1,89)
C1qa<tm1b(EUCOMM)Wtsi>/3J	Total.DrinkingMeth	0.000	0.183	0.001	0.801		19.11(3,207)	2.02(3,207)	13.3(1,69)	0.19(1,69)
C1qa<tm1b(EUCOMM)Wtsi>/3J	Total.DrinkingNicotine	0.404	0.937	0.629	0.996		1.08(3,201)	0.3(3,201)	0.29(1,67)	0.02(1,67)
C9<tm1.1(KOMP)Vlcg>/J	DrugWeightMeth	0.000	0.988	0.936	0.556		19.97(3,207)	0.09(3,207)	0.18(1,69)	1.07(1,69)
C9<tm1.1(KOMP)Vlcg>/J	PreferenceMeth	0.000	0.024	0.820	0.088		27.35(3,195)	4.44(3,195)	0.6(1,65)	6.28(1,65)
C9<tm1.1(KOMP)Vlcg>/J	Total.DrinkingMeth	0.000	0.183	0.001	0.801		10.91(3,198)	2.56(3,198)	15.35(1,66)	0.46(1,66)
C9<-tm1.1(KOMP)Vlcg>/J	DrugWeightNicotine	0.000	0.718	0.817	0.991		64.64(3,198)	1.88(3,198)	4.16(1,66)	0.01(1,66)
C9<-tm1.1(KOMP)Vlcg>/J	PreferenceNicotine	1.000	1.000	1.000	1.000		1	1	1	1
C9<-tm1.1(KOMP)Vlcg>/J	Total.DrinkingNicotine	0.011	0.937	0.609	0.921		5.99(3,195)	0.34(3,195)	1.13(1,65)	2.11(1,65)
Cfb<tm1.1(KOMP)Wtsi>/J	DrugWeightEthanol	0.000	0.002	0.000	0.036	↓	169.64(3,267)	5.65(3,267)	33.44(1,90)	5.54(1,90)
Cfb<tm1.1(KOMP)Wtsi>/J	DrugWeightMeth	0.000	0.988	0.936	0.556		16.68(3,198)	0.08(3,198)	0.05(1,66)	1.04(1,66)
Cfb<tm1.1(KOMP)Wtsi>/J	DrugWeightNicotine	0.000	0.718	0.945	0.991		36.94(3,165)	0.61(3,165)	0.03(1,55)	0(1,55)
Cfb<tm1.1(KOMP)Wtsi>/J	PreferenceEthanol	0.000	0.700	0.001	0.703		8.75(3,265)	1.05(3,265)	15.09(1,90)	0.24(1,90)
Cfb<tm1.1(KOMP)Wtsi>/J	PreferenceMeth	0.000	0.024	0.820	0.506		23.33(3,189)	4.19(3,189)	0.65(1,63)	1.18(1,63)
Cfb<tm1.1(KOMP)Wtsi>/J	PreferenceNicotine	1.000	1.000	1.000	1.000		1	1	1	1
Cfb<tm1.1(KOMP)Wtsi>/J	Total.DrinkingEthanol	0.000	0.798	0.000	0.004	↓	18.21(3,265)	0.61(3,265)	36.14(1,90)	11.68(1,90)
Cfb<tm1.1(KOMP)Wtsi>/J	Total.DrinkingMeth	0.000	0.183	0.001	0.599		18.14(3,192)	2.21(3,192)	15.24(1,64)	0.95(1,64)
Cfb<tm1.1(KOMP)Wtsi>/J	Total.DrinkingNicotine	0.095	0.937	0.609	0.996		2.3(3,165)	0.22(3,165)	0.38(1,55)	0.01(1,55)
Cp<tm1b(KOMP)Wtsi>/J	DrugWeightEthanol	0.000	0.002	0.000	0.104		188.25(3,268)	6.26(3,268)	37.84(1,90)	3.09(1,90)
Cp<tm1b(KOMP)Wtsi>/J	DrugWeightMeth	0.000	0.988	0.991	0.556		23.16(3,234)	0.11(3,234)	0(1,78)	1.36(1,78)
Cp<tm1b(KOMP)Wtsi>/J	DrugWeightNicotine	0.000	0.718	0.945	0.991		39.14(3,180)	0.65(3,180)	0.03(1,60)	0.15(1,60)
Cp<tm1b(KOMP)Wtsi>/J	PreferenceEthanol	0.000	0.790	0.001	0.218		10.67(3,266)	0.64(3,266)	16.47(1,90)	2.12(1,90)
Cp<tm1b(KOMP)Wtsi>/J	PreferenceMeth	0.000	0.079	0.820	0.399		35.86(3,222)	2.39(3,222)	1.49(1,74)	2.11(1,74)
Cp<tm1b(KOMP)Wtsi>/J	PreferenceNicotine	1.000	1.000	1.000	1.000		1	1	1	1
Cp<tm1b(KOMP)Wtsi>/J	Total.DrinkingEthanol	0.000	0.798	0.000	0.177		23.53(3,266)	0.67(3,266)	44.76(1,90)	2.45(1,90)
Cp<tm1b(KOMP)Wtsi>/J	Total.DrinkingMeth	0.000	0.183	0.001	0.807		16.74(3,228)	2.3(3,228)	13.4(1,76)	0.07(1,76)
Cp<tm1b(KOMP)Wtsi>/J	Total.DrinkingNicotine	0.088	0.937	0.609	0.996		2.52(3,180)	0.24(3,180)	0.42(1,60)	0.65(1,60)
Dnajb3<tm1.1(KOMP)Vlcg>/J	DrugWeightEthanol	0.000	0.002	0.000	0.000	↑	222.65(3,268)	5.95(3,268)	35.57(1,90)	58.9(1,90)
Dnajb3<tm1.1(KOMP)Vlcg>/J	DrugWeightMeth	0.000	0.988	0.936	0.556		17.38(3,198)	0.04(3,198)	0.15(1,66)	0.78(1,66)
Dnajb3<tm1.1(KOMP)Vlcg>/J	DrugWeightNicotine	0.000	0.718	0.945	0.991		34.72(3,195)	0.58(3,195)	0.03(1,65)	0.14(1,65)
Dnajb3<tm1.1(KOMP)Vlcg>/J	PreferenceEthanol	0.000	0.790	0.001	0.000	↑	12.61(3,266)	0.49(3,266)	15.6(1,90)	31.41(1,90)
Dnajb3<tm1.1(KOMP)Vlcg>/J	PreferenceMeth	0.000	0.033	0.820	0.399		23.83(3,189)	3.49(3,189)	0.22(1,63)	2.24(1,63)
Dnajb3<tm1.1(KOMP)Vlcg>/J	PreferenceNicotine	1.000	1.000	1.000	1.000		1	1	1	1
Dnajb3<tm1.1(KOMP)Vlcg>/J	Total.DrinkingEthanol	0.000	0.798	0.000	0.163		20.07(3,266)	0.75(3,266)	40.9(1,90)	3.2(1,90)
Dnajb3<tm1.1(KOMP)Vlcg>/J	Total.DrinkingMeth	0.000	0.183	0.002	0.801		18.27(3,192)	1.98(3,192)	11.25(1,64)	0.14(1,64)
Dnajb3<tm1.1(KOMP)Vlcg>/J	Total.DrinkingNicotine	0.088	0.937	0.609	0.921		2.62(3,195)	0.25(3,195)	0.43(1,65)	1.49(1,65)
Dnase112<tm1.1(KOMP)Wtsi>/J	DrugWeightEthanol	0.000	0.002	0.000	0.767		178.73(3,267)	6.4(3,267)	31.51(1,90)	0.09(1,90)
Dnase112<tm1.1(KOMP)Wtsi>/J	DrugWeightMeth	0.000	0.988	0.969	0.556		17.79(3,216)	0.23(3,216)	0.01(1,72)	0.68(1,72)
Dnase112<tm1.1(KOMP)Wtsi>/J	DrugWeightNicotine	0.000	0.718	0.945	0.991		35(3,165)	0.58(3,165)	0.03(1,55)	0.01(1,55)
Dnase112<tm1.1(KOMP)Wtsi>/J	PreferenceEthanol	0.000	0.700	0.001	0.730		10.76(3,265)	2.82(3,265)	13.97(1,90)	0.16(1,90)
Dnase112<tm1.1(KOMP)Wtsi>/J	PreferenceMeth	0.000	0.116	0.851	0.481		16.89(3,207)	2.04(3,207)	0.06(1,69)	1.57(1,69)
Dnase112<tm1.1(KOMP)Wtsi>/J	PreferenceNicotine	1.000	1.000	1.000	1.000		1	1	1	1
Dnase112<tm1.1(KOMP)Wtsi>/J	Total.DrinkingEthanol	0.000	0.798	0.000	0.196		18.05(3,265)	1.39(3,265)	35.35(1,90)	2.06(1,90)
Dnase112<tm1.1(KOMP)Wtsi>/J	Total.DrinkingMeth	0.000	0.183	0.001	0.108		15.24(3,210)	1.96(3,210)	13.86(1,70)	4.29(1,70)
Dnase112<tm1.1(KOMP)Wtsi>/J	Total.DrinkingNicotine	0.095	0.937	0.609	0.996		2.3(3,165)	0.22(3,165)	0.38(1,55)	0.02(1,55)
Epb4.114a<tm1b(KOMP)Mbp>/2J	DrugWeightEthanol	0.000	0.007	0.000	0.171		183.11(3,268)	4.13(3,268)	21.03(1,90)	2.17(1,90)
Epb4.114a<tm1b(KOMP)Mbp>/2J	DrugWeightMeth	0.000	0.988	0.936	0.556		17.83(3,216)	0.66(3,216)	0.74(1,72)	2.35(1,72)
Epb4.114a<tm1b(KOMP)Mbp>/2J	DrugWeightNicotine	0.000	0.718	0.945	0.991		35.02(3,183)	0.58(3,183)	0.02(1,61)	0.13(1,61)
Epb4.114a<tm1b(KOMP)Mbp>/2J	PreferenceEthanol	0.000	0.790	0.002	0.200		10.99(3,266)	0.41(3,266)	9.83(1,90)	2.38(1,90)
Epb4.114a<tm1b(KOMP)Mbp>/2J	PreferenceMeth	0.000	0.044	0.820	0.562		28.39(3,207)	3.12(3,207)	1.76(1,69)	0.7(1,69)
Epb4.114a<tm1b(KOMP)Mbp>/2J	PreferenceNicotine	1.000	1.000	1.000	1.000		1	1	1	1
Epb4.114a<tm1b(KOMP)Mbp>/2J	Total.DrinkingEthanol	0.000	0.798	0.000	0.655		17.81(3,266)	1.08(3,266)	31.69(1,90)	0.2(1,90)
Epb4.114a<tm1b(KOMP)Mbp>/2J	Total.DrinkingMeth	0.000	0.335	0.001	0.308		11.21(3,210)	1.29(3,210)	13.6(1,70)	2.08(1,70)
Epb4.114a<tm1b(KOMP)Mbp>/2J	Total.DrinkingNicotine	0.088	0.937	0.609	0.996		2.47(3,183)	0.23(3,183)	0.4(1,61)	0.34(1,61)
Far2<tm2b(KOMP)Wtsi>/2J	DrugWeightEthanol	0.000	0.002	0.000	0.006	↓	155.92(3,259)	5.59(3,259)	31.47(1,87)	9.3(1,87)
Far2<tm2b(KOMP)Wtsi>/2J	DrugWeightMeth	0.000	0.988	0.936	0.022	↑	16.26(3,207)	0.2(3,207)	2.45(1,69)	10.59(1,69)
Far2<tm2b(KOMP)Wtsi>/2J	DrugWeightNicotine	0.000	0.718	0.945	0.991		38.65(3,198)	0.64(3,198)	0.03(1,66)	0(1,66)
Far2<tm2b(KOMP)Wtsi>/2J	PreferenceEthanol	0.000	0.700	0.001	0.003		9.76(3,256)	0.97(3,256)	14.13(1,87)	12.31(1,87)
Far2<tm2b(KOMP)Wtsi>/2J	PreferenceMeth	0.000	0.331	0.820	0.088		23.92(3,198)	1.15(3,198)	0.15(1,66)	6.72(1,66)
Far2<tm2b(KOMP)Wtsi>/2J	PreferenceNicotine	1.000	1.000	1.000	1.000		1	1	1	1
Far2<tm2b(KOMP)Wtsi>/2J	Total.DrinkingEthanol	0.000	0.798	0.000	0.304		18.3(3,256)	0.56(3,256)	34.03(1,87)	1.22(1,87)
Far2<tm2b(KOMP)Wtsi>/2J	Total.DrinkingMeth	0.000	0.183	0.001	0.003	↑	10.32(3,201)	2.13(3,201)	15.46(1,67)	15.69(1,67)
Far2<tm2b(KOMP)Wtsi>/2J	Total.DrinkingNicotine	0.088	0.937	0.609	0.996		2.74(3,198)	0.26(3,198)	0.45(1,66)	0(1,66)
Gipc3<tm1b(KOMP)Wtsi>/J	DrugWeightEthanol	0.000	0.002	0.000	0.484		156.82(3,263)	5.76(3,263)	29.98(1,90)	0.62(1,90)
Gipc3<tm1b(KOMP)Wtsi>/J	DrugWeightMeth	0.000	0.988	0.936	0.708		14.78(3,216)	0.67(3,216)	0.06(1,72)	0.14(1,72)

Gipc3<tm1b(KOMP)Wtsi>/J	DrugWeightNicotine	0.000	0.718	0.945	0.991		40.77(3,201)	0.68(3,201)	0.03(1,67)	0.09(1,67)
Gipc3<tm1b(KOMP)Wtsi>/J	PreferenceEthanol	0.000	0.790	0.001	0.840		8.12(3,260)	0.48(3,260)	14.31(1,90)	0.04(1,90)
Gipc3<tm1b(KOMP)Wtsi>/J	PreferenceMeth	0.000	0.052	0.820	0.562		26.32(3,207)	2.93(3,207)	0.84(1,69)	0.86(1,69)
Gipc3<tm1b(KOMP)Wtsi>/J	PreferenceNicotine	1.000	1.000	1.000	1.000		1	1	1	1
Gipc3<tm1b(KOMP)Wtsi>/J	Total.DrinkingEthanol	0.000	0.798	0.000	0.177		15.88(3,260)	0.8(3,260)	31.15(1,90)	2.56(1,90)
Gipc3<tm1b(KOMP)Wtsi>/J	Total.DrinkingMeth	0.003	0.940	0.011	0.801		4.9(3,210)	0.13(3,210)	6.85(1,70)	0.15(1,70)
Gipc3<tm1b(KOMP)Wtsi>/J	Total.DrinkingNicotine	0.088	0.937	0.609	0.996		2.78(3,201)	0.26(3,201)	0.46(1,67)	0.35(1,67)
Hdac10<tm1.1(KOMP)Mbp>/J	DrugWeightEthanol	0.000	0.005	0.000	0.006	↑	199.04(3,268)	4.61(3,268)	23.89(1,90)	9.82(1,90)
Hdac10<tm1.1(KOMP)Mbp>/J	DrugWeightMeth	0.000	0.988	0.936	0.556		20.51(3,213)	0.05(3,213)	0.05(1,71)	1.31(1,71)
Hdac10<tm1.1(KOMP)Mbp>/J	DrugWeightNicotine	0.000	0.718	0.945	0.991		38.88(3,195)	0.64(3,195)	0.03(1,65)	0.23(1,65)
Hdac10<tm1.1(KOMP)Mbp>/J	PreferenceEthanol	0.000	0.700	0.001	0.000	↑	11.6(3,266)	1.13(3,266)	11.26(1,90)	17.88(1,90)
Hdac10<tm1.1(KOMP)Mbp>/J	PreferenceMeth	0.000	0.062	0.820	0.399		34.03(3,204)	2.67(3,204)	1.08(1,68)	2.07(1,68)
Hdac10<tm1.1(KOMP)Mbp>/J	PreferenceNicotine	1.000	1.000	1.000	1.000		1	1	1	1
Hdac10<tm1.1(KOMP)Mbp>/J	Total.DrinkingEthanol	0.000	0.798	0.000	0.196		17.45(3,266)	0.98(3,266)	22.09(1,90)	2.12(1,90)
Hdac10<tm1.1(KOMP)Mbp>/J	Total.DrinkingMeth	0.000	0.183	0.000	0.801		11.53(3,207)	1.99(3,207)	18.66(1,69)	0.35(1,69)
Hdac10<tm1.1(KOMP)Mbp>/J	Total.DrinkingNicotine	0.088	0.937	0.609	0.921		2.65(3,195)	0.25(3,195)	0.44(1,65)	1.96(1,65)
Hspb2<tm1.1(KOMP)Vlcg>/J	DrugWeightEthanol	0.000	0.076	0.000	0.000	↑	183.02(3,253)	2.32(3,253)	19.5(1,85)	17.6(1,85)
Hspb2<tm1.1(KOMP)Vlcg>/J	DrugWeightMeth	0.000	0.988	0.936	0.556		20.19(3,213)	0.08(3,213)	0.05(1,71)	0.62(1,71)
Hspb2<tm1.1(KOMP)Vlcg>/J	DrugWeightNicotine	0.000	0.718	0.945	0.991		34.1(3,195)	0.57(3,195)	0.02(1,65)	0.83(1,65)
Hspb2<tm1.1(KOMP)Vlcg>/J	PreferenceEthanol	0.000	0.707	0.002	0.011	↑	12.25(3,251)	0.89(3,251)	9.79(1,85)	8.47(1,85)
Hspb2<tm1.1(KOMP)Vlcg>/J	PreferenceMeth	0.000	0.024	0.820	0.943		22.41(3,204)	4.26(3,204)	0.2(1,68)	0.01(1,68)
Hspb2<tm1.1(KOMP)Vlcg>/J	PreferenceNicotine	1.000	1.000	1.000	1.000		1	1	1	1
Hspb2<tm1.1(KOMP)Vlcg>/J	Total.DrinkingEthanol	0.000	0.905	0.000	0.165		14.9(3,251)	0.19(3,251)	31.44(1,85)	2.87(1,85)
Hspb2<tm1.1(KOMP)Vlcg>/J	Total.DrinkingMeth	0.000	0.183	0.002	0.045		19.54(3,207)	2.09(3,207)	11.01(1,69)	8.39(1,69)
Hspb2<tm1.1(KOMP)Vlcg>/J	Total.DrinkingNicotine	0.088	0.937	0.609	0.921		2.54(3,192)	0.24(3,192)	0.41(1,64)	2.22(1,64)
Htr1a<tm1.1(KOMP)Vlcg>/J	DrugWeightEthanol	0.000	0.002	0.000	0.000	↓	164.22(3,268)	5.7(3,268)	34.71(1,90)	16.85(1,90)
Htr1a<tm1.1(KOMP)Vlcg>/J	DrugWeightMeth	0.000	0.988	0.936	0.556		15.02(3,222)	0.67(3,222)	1.01(1,74)	0.75(1,74)
Htr1a<tm1.1(KOMP)Vlcg>/J	DrugWeightNicotine	0.000	0.718	0.945	0.991		42.12(3,201)	0.7(3,201)	0.03(1,67)	0.01(1,67)
Htr1a<tm1.1(KOMP)Vlcg>/J	PreferenceEthanol	0.000	0.790	0.000	0.064		12.79(3,265)	0.42(3,265)	21.05(1,90)	4.64(1,90)
Htr1a<tm1.1(KOMP)Vlcg>/J	PreferenceMeth	0.000	0.033	0.820	0.572		22.67(3,210)	3.54(3,210)	0.16(1,70)	0.51(1,70)
Htr1a<tm1.1(KOMP)Vlcg>/J	PreferenceNicotine	1.000	1.000	1.000	1.000		1	1	1	1
Htr1a<tm1.1(KOMP)Vlcg>/J	Total.DrinkingEthanol	0.000	0.798	0.000	0.000		14.99(3,265)	0.62(3,265)	32.99(1,90)	29.12(1,90)
Htr1a<tm1.1(KOMP)Vlcg>/J	Total.DrinkingMeth	0.000	0.602	0.001	0.801		7.53(3,216)	0.67(3,216)	13.53(1,72)	0.26(1,72)
Htr1a<tm1.1(KOMP)Vlcg>/J	Total.DrinkingNicotine	0.088	0.937	0.609	0.996		2.76(3,201)	0.26(3,201)	0.46(1,67)	0(1,67)
Il12rb2<tm1.1(KOMP)Vlcg>/J	DrugWeightEthanol	0.000	0.004	0.000	0.000	↑	247.77(3,266)	4.97(3,266)	22.2(1,90)	88.48(1,90)
Il12rb2<tm1.1(KOMP)Vlcg>/J	DrugWeightMeth	0.000	0.988	0.936	0.022	↑↓	23.64(3,213)	0.36(3,213)	0.39(1,71)	9.88(1,71)
Il12rb2<tm1.1(KOMP)Vlcg>/J	DrugWeightNicotine	0.000	0.770	0.945	0.991		26.36(3,201)	0.44(3,201)	0.02(1,67)	0.37(1,67)
Il12rb2<tm1.1(KOMP)Vlcg>/J	PreferenceEthanol	0.000	0.823	0.001	0.000	↑	13.33(3,264)	0.3(3,264)	11.2(1,90)	35.7(1,90)
Il12rb2<tm1.1(KOMP)Vlcg>/J	PreferenceMeth	0.000	0.079	0.820	0.187		29.82(3,204)	2.4(3,204)	0.19(1,68)	4.32(1,68)
Il12rb2<tm1.1(KOMP)Vlcg>/J	PreferenceNicotine	1.000	1.000	1.000	1.000		1	1	1	1
Il12rb2<tm1.1(KOMP)Vlcg>/J	Total.DrinkingEthanol	0.000	0.798	0.000	0.004	↑	20.73(3,264)	1.33(3,264)	25.53(1,90)	11.51(1,90)
Il12rb2<tm1.1(KOMP)Vlcg>/J	Total.DrinkingMeth	0.000	0.337	0.001	0.057		22.12(3,207)	1.23(3,207)	13.35(1,69)	6.96(1,69)
Il12rb2<tm1.1(KOMP)Vlcg>/J	Total.DrinkingNicotine	0.088	0.937	0.609	0.996		2.64(3,198)	0.25(3,198)	0.42(1,66)	0.79(1,66)
Lpar6<tm1.1(KOMP)Vlcg>/J	DrugWeightEthanol	0.000	0.005	0.000	0.053		148.98(3,236)	4.73(3,236)	29.87(1,80)	4.52(1,80)
Lpar6<tm1.1(KOMP)Vlcg>/J	PreferenceEthanol	0.000	0.700	0.001	0.005	↓	11.3(3,234)	1.28(3,234)	15.39(1,80)	10.49(1,80)
Lpar6<tm1.1(KOMP)Vlcg>/J	Total.DrinkingEthanol	0.000	0.798	0.000	0.655		14.76(3,234)	0.52(3,234)	32.07(1,80)	0.22(1,80)
Parp8<tm1.1(KOMP)Wtsi>/J	DrugWeightEthanol	0.000	0.002	0.000	0.001	↓	169.92(3,254)	5.57(3,254)	18.51(1,86)	13.82(1,86)
Parp8<tm1.1(KOMP)Wtsi>/J	DrugWeightMeth	0.000	0.988	0.936	0.700		19.35(3,216)	0.09(3,216)	0.65(1,72)	0.19(1,72)
Parp8<tm1.1(KOMP)Wtsi>/J	DrugWeightNicotine	0.000	0.718	0.945	0.991		36.11(3,195)	0.6(3,195)	0.02(1,65)	0.07(1,65)
Parp8<tm1.1(KOMP)Wtsi>/J	PreferenceEthanol	0.000	0.700	0.001	0.274		11.81(3,252)	1.52(3,252)	13.24(1,86)	1.55(1,86)
Parp8<tm1.1(KOMP)Wtsi>/J	PreferenceMeth	0.000	0.058	0.899	0.864		25.65(3,207)	2.79(3,207)	0.02(1,69)	0.06(1,69)
Parp8<tm1.1(KOMP)Wtsi>/J	PreferenceNicotine	1.000	1.000	1.000	1.000		1	1	1	1
Parp8<tm1.1(KOMP)Wtsi>/J	Total.DrinkingEthanol	0.000	0.798	0.000	0.032	↑	14.65(3,252)	1.42(3,252)	21.55(1,86)	6.39(1,86)
Parp8<tm1.1(KOMP)Wtsi>/J	Total.DrinkingMeth	0.000	0.183	0.000	0.308		14.08(3,210)	1.95(3,210)	20.07(1,70)	2.16(1,70)
Parp8<tm1.1(KOMP)Wtsi>/J	Total.DrinkingNicotine	0.088	0.937	0.609	0.996		2.68(3,195)	0.25(3,195)	0.43(1,65)	0.01(1,65)
Pitx3<tm1.1(KOMP)Vlcg>/J	DrugWeightEthanol	0.000	0.005	0.000	0.767		145.62(3,232)	4.49(3,232)	28.74(1,78)	0.09(1,78)
Pitx3<tm1.1(KOMP)Vlcg>/J	DrugWeightMeth	0.000	0.988	0.936	0.589		19.24(3,198)	0.12(3,198)	0.15(1,66)	0.48(1,66)
Pitx3<tm1.1(KOMP)Vlcg>/J	DrugWeightNicotine	0.000	0.718	0.945	0.991		40.7(3,201)	0.67(3,201)	0.03(1,67)	0.13(1,67)
Pitx3<tm1.1(KOMP)Vlcg>/J	PreferenceEthanol	0.000	0.700	0.001	0.271		9.35(3,230)	1.11(3,230)	13.87(1,78)	1.67(1,78)
Pitx3<tm1.1(KOMP)Vlcg>/J	PreferenceMeth	0.000	0.033	0.820	0.864		26.41(3,186)	3.68(3,186)	0.47(1,62)	0.05(1,62)
Pitx3<tm1.1(KOMP)Vlcg>/J	PreferenceNicotine	1.000	1.000	1.000	1.000		1	1	1	1
Pitx3<tm1.1(KOMP)Vlcg>/J	Total.DrinkingEthanol	0.000	0.798	0.000	0.028	↑	13.95(3,230)	0.61(3,230)	34.6(1,78)	6.92(1,78)
Pitx3<tm1.1(KOMP)Vlcg>/J	Total.DrinkingMeth	0.000	0.248	0.001	0.057		11.98(3,192)	1.65(3,192)	11.84(1,64)	6.59(1,64)
Pitx3<tm1.1(KOMP)Vlcg>/J	Total.DrinkingNicotine	0.088	0.937	0.609	0.996		2.73(3,201)	0.26(3,201)	0.45(1,67)	0.07(1,67)
Pnmt<-tm1.1(KOMP)Vlcg>/J	DrugWeightEthanol	0.000	0.005	0.000	0.046	↓	144.28(3,238)	4.53(3,238)	30.96(1,80)	4.94(1,80)
Pnmt<-tm1.1(KOMP)Vlcg>/J	PreferenceEthanol	0.000	0.700	0.001	0.099		9.58(3,236)	1.3(3,236)	17.44(1,80)	3.72(1,80)
Pnmt<-tm1.1(KOMP)Vlcg>/J	Total.DrinkingEthanol	0.000	0.836	0.000	0.165		14.96(3,236)	0.41(3,236)	34.41(1,80)	2.85(1,80)
Rilp12<tm1b (KOMP)Wtsi>/J	DrugWeightEthanol	0.000	0.002	0.000	0.087		191.09(3,268)	6.41(3,268)	25.4(1,90)	3.52(1,90)
Rilp12<tm1b (KOMP)Wtsi>/J	DrugWeightMeth	0.000	0.988	0.936	0.556		18.47(3,216)	0.08(3,216)	0.1(1,72)	2.19(1,72)
Rilp12<tm1b (KOMP)Wtsi>/J	DrugWeightNicotine	0.000	0.718	0.945	0.991		41.17(3,198)	0.68(3,198)	0.03(1,66)	0(1,66)
Rilp12<tm1b (KOMP)Wtsi>/J	PreferenceEthanol	0.000	0.700	0.002	0.003	↑	10.87(3,266)	1.29(3,266)	10.03(1,90)	11.76(1,90)
Rilp12<tm1b (KOMP)Wtsi>/J	PreferenceMeth	0.000	0.024	0.820	0.572		24.82(3,207)	4.56(3,207)	1.27(1,69)	0.55(1,69)
Rilp12<tm1b (KOMP)Wtsi>/J	PreferenceNicotine	1.000	1.000	1.000	1.000		1	1	1	1
Rilp12<tm1b (KOMP)Wtsi>/J	Total.DrinkingEthanol	0.000	0.798	0.000	0.004	↓	15.84(3,266)	1.46(3,266)	27.85(1,90)	11.09(1,90)
Rilp12<tm1b (KOMP)Wtsi>/J	Total.DrinkingMeth	0.000	0.183	0.000	0.073		21.12(3,210)	2.06(3,210)	23.41(1,70)	5.64(1,70)
Rilp12<tm1b (KOMP)Wtsi>/J	Total.DrinkingNicotine	0.088	0.937	0.609	0.996		2.77(3,198)	0.26(3,198)	0.46(1,66)	0.03(1,66)

Supplemental Table 13

Figure Abreviation	Phenotypic Value	Dim.1	Dim.2	Dim.3	Dim.4	Dim.5
HB_THP	HB_Parameters.Total.Hole.Pokes	0.59251	-0.37163	0.202082	0.435809	-0.04775
LD_LSTS	LD_Collected.Values.Left.Side.Time.Spent	0.465306	0.616421	-0.22466	-0.10912	-0.09043
OF_CPT	OFA_Outputs.Center.Permanence.Time	0.693669	0.173382	-0.23595	-0.22048	-0.11668
OF_DT5	OFA_Outputs.Distance.Traveled.First.Five.Min	0.035671	-0.15228	0.838258	-0.22682	-0.33378
OF_DTS	OFA_Outputs.Distance.Traveled.Slope	0.06745	0.048413	-0.76961	0.543343	0.089992
OF_DTT	OFA_Outputs.Distance.Traveled.Total	0.167815	-0.04638	0.834497	0.24625	-0.25573
OF_NRT	OFA_Outputs.Number.of.Rears.Total	0.616238	-0.19935	-0.01666	0.211267	-0.23027
SR_GPPIPCT	PPI_Startle.Amplitude.Percent.PPI.Global	-0.29198	0.395538	0.222919	0.236624	0.414243
TS_LI	TST_Results.Latency.to.immobility	0.336285	-0.21475	0.132426	-0.05221	-0.40279
TS_TI	TST_Results.Time.immobile	0.255836	-0.25457	-0.16867	0.041493	0.728604
E_DW	DrugWeightEthanol_Estimate	0.778008	0.190419	0.230541	-0.35864	0.348551
E_PREF	DrugWeightMeth_Estimate	0.870577	-0.0099	0.054508	-0.37188	0.126289
E_TOTD	DrugWeightNicotine_Estimate	0.038993	0.674089	0.404321	-0.20763	0.351923
E_DWATER	PreferenceEthanol_Estimate	-0.8452	0.20872	0.083229	0.297021	-0.1253
M_DW	PreferenceMeth_Estimate	-0.17753	0.610479	-0.19189	-0.535	-0.23682
M_PREF	PreferenceNicotine_Estimate	-0.2159	0.288397	0.086121	-0.57851	-0.06941
M_TOTD	Total.DrinkingEthanol_Estimate	-0.21715	0.846611	-0.28185	-0.21056	-0.11951
M_DWATER	Total.DrinkingMeth_Estimate	-0.31146	0.442664	-0.03202	0.269403	-0.10914
N_DW	Total.DrinkingNicotine_Estimate	0.012017	0.524387	0.622312	0.388775	0.144365
N_PREF	Water.DrinkingEthanol_Estimate	-0.46926	-0.28872	0.623245	-0.20906	0.367393
N_TOTD	Water.DrinkingMeth_Estimate	0.386015	0.648858	0.32357	0.445954	-0.02986
N_DWATER	Water.DrinkingNicotine_Estimate	0.477781	0.647638	0.011733	0.474565	-0.04933

Supplemental Table 14

Methamphetamine Preference Statitsics	F-Statistic									P-Values						
Genes	(Intercept)	Strain	Dose	Sex	Strain:Dose	Strain:Sex	Dose:Sex	Strain:Dose:Sex	(Intercept)	Strain	Dose	Sex	Strain:Dose	Strain:Sex	Dose:Sex	Strain:Dose:Sex
Irf8	268.06675 (11,40)	12.33555 (1,51)	27.64035 (3,140)	0.60256(1,51)	3.69117 (3,140)	0300336 (1,51)	0.20258 (3,140)	0.92638 (3,140)	<0.0001	0.0009	<0.0001	0.44	**0.01**	0.95	0.89	0.43
Dnmt3a	282.53368 (1, 142)	0.78618 (1,52)	36.37813 (3,142)	0.77900 (1,52)	6.87866 (3,142)	0.00140 (1,52)	0.87238 (3,142)	0.04695 (3, 142)	<0.0001	0.3796	<0.0001	0.38	**0.00**	0.97	0.46	0.99
Cp	251.38121 (1,141)	2.56083 (1,51)	32.69355 (3,141)	1.77242 (1,51)	4.94532 (3,141)	0.67250 (1,51)	1.90631 (3,141)	0.51557 (3,141)	<0.0001	0.1157	<0.0001	0.19	**0.00**	0.42	0.13	0.67
C3	266.68919 (1,139)	2.34730 (1,51)	20.84118 (3,139)	0.0045 (1,51)	0.86907 (3,139)	1.54889 (1,51)	0.76422 (3,139(	0.98245 (3,139)	<0.0001	0.1317	<0.0001	0.95	0.46	0.22	0.52	0.40
Dnaja4	227.02587 (1,129)	4.66353 (1,47)	24.63781 (3,129)	1.77117 (1,47)	3.37420 (3,129)	1.40046 (1,47)	0.38022 (3,129)	1.30536 (3,129)	<0.0001	0.0359	<0.0001	0.19	0.02	0.24	0.77	0.28
Lrrrc15	255.4054 (1,138)	0.03489 (1,51)	38.82401 (3,138)	0.47106 (1,51)	3.23438 (3,138)	0.08782 (1,51)	0.86871 (3,138)	0.47292 (3,138)	<0.0001	0.8526	<0.0001	0.50	0.02	0.77	0.46	0.70
Htr7	304.24163 (1,140)	10.12251 (1,51)	36.13401 (3,140)	0.28176 (1,51)	6.676159 (3,140)	0.26056 (1,51)	1.53015 (3,140)	0.76314 (3,140)	<0.0001	0.0025	<0.0001	0.60	**0.00**	0.61	0.21	0.52
Zbtb4	276.8275 (1,139)	6.91611 (1,51)	20.95922 (3,139)	5.96888 (1,51)	0.95507 (3,139)	6.32621 (1,51)	0.28086 (3,139)	2.57162 (3,139	<0.0001	0.0113	<0.0001	0.02	0.42	0.02	0.84	0.06
Tmod2	316.4013 (1,140)	15.2263 (1,51)	14.0085 (3,140)	1.3812 (1,51)	2.9199 (3,140)	0.2710 (1,51)	1.0462 (3,140)	1.0460 (3,140)	<0.0001	0.0003	<0.0001	0.25	0.04	0.60	0.37	0.37
Elof1	238.49957 (1,140)	2.3463 (1,51)	24.23174 (3,140)	0.06436 (1,51)	0.70776 (3,140)	0.65086 (1,51)	1.74759 (3,140)	1.00668 (3,140)	<0.0001	0.1318	<0.0001	0.80	0.55	0.42	0.16	0.39
Rap2b	355.9156 (1,140)	25.9923 (1,51)	21.4053 (3,140)	0.2859 (1,51)	1.4608 (3,140)	0.2255 (1,51)	1.4632 (3,140)	0.7517 (3,140)	<0.0001	0.0001	<0.0001	0.60	0.23	0.64	0.23	0.52
Myh10	293.42987 (1,142)	8.26428 (1,51)	28.39521 (3,142)	0.04393 (1,51)	3.71712 (3,142)	0.82874 (1,51)	0.54764 (3,142)	4.57756 (3,142)	<0.0001	0.0059	<0.0001	0.83	**0.01**	0.37	0.65	0.00
Stx19	255.56279 (1,139)	1.37726 (1,51)	27.98946 (3,139)	3.12581 (1,51)	1.65651 (3,139)	2.08730 (1,51)	0.90742 (3,139)	0.11991 (3,139)	<0.0001	0.246	<0.0001	0.08	0.18	0.15	0.44	0.95
Gpr142	262.89222 (1,138)	5.00088 (1,51)	24.63009 (3,138)	0.96521 (1,51)	1.67760 (3,138)	0.07004 (1,51)	1.54203 (3,138)	0.60844 (3,138)	<0.0001	0.0297	<0.0001	0.33	0.17	0.79	0.21	0.61
Stk36	264.76879 (1,139)	1.79136 (1,51)	28.08908 (3,139)	0.81239 (1,51)	2.29508 (3,139)	0.00777 (1,51)	0.56840 (3,139)	0.712385 (3,139)	<0.0001	0.1867	<0.0001	0.37	0.08	0.93	0.64	0.55

Supplemental Table 15

Methamphetamine Consumption Statistics	F-Statistic								P-values							
Genes	(Intercept)	Strain	Dose	Sex	Strain:Dose	Strain:Sex	Dose:Sex	Strain:Dose:Sex	(Intercept)	Strain	Dose	Sex	Strain:Dose	Strain:Sex	Dose:Sex	rain:Dose:Sex
**Irf8**	265.64367 (1,153)	7.72032 (1,51)	10.48720 (3,153)	3.37907 (1,51)	0.18140 (3,153)	0.44968 (1,51)	0.56015 (3,1530	1.12959 (3,153)	<0.0001	0.0088	<0.0001	0.0719	0.9089	0.5055	0.6421	0.339
Dnmt3a	248.91491 (1,156)	1.21778 (1,52)	16.44260 (3,156)	1.07802 (1,52)	1.75699 (3,156)	0.71724 (1,52)	0.29802 (3,156)	0.24484 (3,156)	<0.0001	0.2749	<0.0001	0.3039	1.58E-01	4.01E-01	0.8268	0.8649
Cp	199.37698 (1,153)	1.20225 (1,51)	16.22559 (3,153)	1.49986 (1,51)	3.07380 (3,153)	0.01331 (1,51)	0.53873 (3,153)	0.75860 (3,153)	<0.0001	0.278	<0.0001	0.2263	**0.0295**	0.9086	0.6565	0.59
C3	248.34618 (1,153)	1.03036 (1,51)	16.07924 (3,153)	1.87334 (1,51)	0.46015 (3,153)	0.02133 (1,51)	0.27673 (3,153)	0.59487 (3,1153)	<0.0001	0.3149	<0.0001	0.1771	0.7105	0.8845	0.8421	0.6193
Dnaja4	220.67885 (1,141)	0.89180 (1,47)	12.07828 (3,131)	0.29086 (1,47)	0.43013 (1,141)	2.51375 (1,47)	0.11890 (3,141)	1.24474 (3,141)	<0.0001	0.3498	<0.0001	0.5922	0.7317	0.1196	0.9489	0.2959
Lrrrc15	224.56918 (1,153)	0.27829 (1,51)	10.45722 (3,153)	1.21609 (1,51)	0.74345 (3,153)	0.24154 (1,51)	0.11556 (3,153)	0.58723 (3,153)	<0.0001	0.6001	<0.0001	0.2753	0.5277	0.6252	0.926	0.6243
Htr7	267.73890 (1,153)	5.84604 (1,51)	15.56964 (3,153)	3.66499 (1,51)	0.36237 (3,153)	0.56278 (1,51)	0.06769 (3,153)	0.25925 (3,153)	<0.0001	0.0192	<0.0001	0.0612	0.7803	0.4566	0.977	0.8546
Zbtb4	256.14946 (1,153)	3.23506 (1,51)	24.35146 (3,153)	0.03899 (1,51)	2.05738 (3,153)	3.74557 (1,51)	0.070477 (3,153)	0.94275 (3,153)	<0.0001	0.078	<0.0001	0.8442	0.1082	0.0585	0.9735	0.4216
**Tmod2**	244.93221 (1,153)	8.91677 (1,51)	23.11219 (3,153)	2.98431 (1,51)	4.70665 (3,153)	0.38573 (1,51)	0.31503 (3,153)	0.28056 (3,153)	<0.0001	0.0043	<0.0001	0.0901	0.0036	0.5373	0.8145	0.8394
Elof1	244.78816 (1,153)	0.49279 (1,51)	17.35514 (3,153)	1.83198 (1,51)	0.30793 (3,1530	0.03347 (1,51)	0.20289 (3,153)	0.19181 (3,153)	<0.0001	0.4859	<0.0001	0.1819	0.8196	0.8556	0.8943	0.9019
**Rap2b**	300.16776 (1,153)	15.13764 (1,51)	14.22713 (3,153)	3.05236 (1,51)	3.47943 (3,153)	0.93332 (1,51)	0.54341 (3,153)	0.69253 (3,153)	<0.0001	0.0003	<0.0001	0.0866	0.0175	0.3386	0.6533	0.5579
Myh10	250.98109 (1,153)	2.34176 (1,51)	14.45498 (3,153)	0.88131 (1,51)	1.07970 (3,153)	0.60809 (1,51)	0.87077 (3,153)	3.25148 (3,153)	<0.0001	0.1321	<0.0001	0.3523	**0.3595**	0.4391	0.4577	0.0235
Stx19	218.65442 (1,153)	0.06179 (1,51)	16.17481 (3,153)	0.69707 (1,51)	0.91736 (3,153)	0.82174 (1.51)	0.05449 (3,153)	0.56800 (3,153)	<0.0001	0.8047	<0.0001	0.4077	0.4341	0.3689	0.9832	0.6369
Gpr142	212.75657 (1,153)	2.82319 (1,51)	14.45989 (3,153)	2.73678 (1,51)	0.31082 (3,153)	0.29293 (1,51)	0.20204 (3,153)	0.14195 (3,153)	<0.0001	0.099	<0.0001	0.1042	0.8175	0.5907	0.8948	0.9347
Stk36	241.64398 (1,153)	0.08540 (1,51)	13.13474 (3,153)	1.26917 (1,51)	1.13661 (3,153)	0.38204 (1,51)	0.08157 (3,153)	0.55540 (3,153)	<0.0001	0.7713	<0.0001	0.2652	0.3362	0.5393	0.97	0.6453

Supplemental Table 16

DID Statistics	F-Statistic								P-values						
	**(Intercept)**	**Day**	**Day:Sex**	**Sex**	**Strain**	**Strain:Day**	**Strain:Day:Sex**	**Strain:Sex**	**Day:Sex**	**Sex**	**Strain**	**Strain:Day**	**Strain:Day**	**Strain:Sex**	**Relative to Control**
Irf8	698.25(1,142)	86.39(3,142)	16.09(3,142)	99.74(1,48)	13.38(1,48)	7.95(3,142)	2.18(3,142)	5.89(1,48)	5.25E-09	3.26E-13	0.00537	0.001049	0.728807	0.107794	↓
Dnmt3a	512.53(1,124)	91.37(3,124)	16.92(3,124)	83.77(1,42)	0.78(1,42)	0.6(3,124)	0.37(3,124)	0.06(1,42)	3.44E-09	1.57E-11	0.629825	0.970346	0.933077	0.805673	
Cp	862.08(1,142)	118.1(3,142)	20.47(3,142)	137.51(1,48)	0.25(1,48)	0.48(3,142)	0.52(3,142)	0.1(1,48)	8.35E-11	6.04E-15	0.812483	0.970346	0.933077	0.799234	
C3	737.53(1,141)	95.22(3,141)	20.72(3,141)	114.7(1,48)	0.04(1,48)	0.66(3,141)	0.39(3,141)	0.16(1,48)	7.48E-11	7.05E-14	0.847355	0.970346	0.933077	0.787906	
Dnaja4	350.17(1,139)	77.01(3,139)	16.75(3,139)	40.33(1,47)	6.46(1,47)	1.75(3,139)	0.56(3,139)	0.61(1,47)	3.15E-09	7.92E-08	0.061111	0.827719	0.933077	0.71871	
Lrrrc15	765.49(1,142)	125.66(3,142)	28.09(3,142)	108.96(1,48)	1.38(1,48)	0.46(3,142)	0.67(3,142)	0.29(1,48)	3.89E-13	1.04E-13	0.523132	0.970346	0.933077	0.71871	
Htr7	895.7(1,142)	115.01(3,142)	27.03(3,142)	135.66(1,48)	0.08(1,48)	0.08(3,142)	0.47(3,142)	0.48(1,48)	3.89E-13	6.04E-15	0.847355	0.973919	0.933077	0.71871	
Zbtb4	969.46(1,142)	108.38(3,142)	12.99(3,142)	113.68(1,48)	2.48(1,48)	0.99(3,142)	3.57(3,142)	11.72(1,48)	1.51E-07	7.05E-14	0.344545	0.848143	0.265901	0.021697	M↑F↓
Tmod2	733.8(1,139)	119.57(3,139)	15.32(3,139)	87.87(1,47)	1.93(1,47)	1.26(3,139)	1.92(3,139)	8.39(1,47)	1.22E-08	2.81E-12	0.416031	0.827719	0.728807	0.048432	M↑F↓
Elof1	832.32(1,142)	128.21(3,142)	26.56(3,142)	133.71(1,48)	7.6(1,48)	0.07(3,142)	0.3(3,142)	1.34(1,48)	4.53E-13	6.04E-15	0.046558	0.973919	0.933077	0.536126	
Rap2b	841.9(1,142)	119.85(3,142)	21.73(3,142)	99.64(1,48)	0.92(1,48)	1.05(3,142)	0.19(3,142)	4.54(1,48)	2.95E-11	3.26E-13	0.629825	0.848143	0.949105	0.162683	
Myh10	844.89(1,142)	109.06(3,142)	20.26(3,142)	112.4(1,48)	0.06(1,48)	1.38(3,142)	0.97(3,142)	2.3(1,48)	8.52E-11	7.05E-14	0.847355	0.827719	0.933077	0.461146	
Stx19	580.23(1,141)	119.5(3,141)	25.63(3,141)	104.45(1,48)	0.05(1,48)	0.42(3,141)	0.51(3,141)	0.53(1,48)	9.24E-13	1.91E-13	0.847355	0.970346	0.933077	0.71871	
Gpr142	732.7(1,142)	99.15(3,142)	18(3,142)	102.37(1,48)	0.47(1,48)	1.42(3,142)	0.62(3,142)	2(1,48)	8.36E-10	2.44E-13	0.705109	0.827719	0.933077	0.463931	
Stk36	615.37(1,140)	109.78(3,140)	20.31(3,140)	112.17(1,48)	0.7(1,48)	0.32(3,140)	0.37(3,140)	0.36(1,48)	8.52E-11	7.05E-14	0.629825	0.973919	0.933077	0.71871	

Supplemental Table 17

			Description of Geneset
Hdac10			
	GS233344	KEGG Geneset	"Alcoholism" pathway genes,
	GS233499	KEGG Geneset	"Alcoholism" pathway genes
	GS233931	KEGG Geneset	"Alcoholism" pathway genes
	GS86789	[DRG]	Table S1: Cocaine Regulation of H3 Acetylation. (provisional)
Lpar6			
	GS86977	[DRG]	Table S1: All transcripts significantly different in abundance between
	GS84277	(Published QTL)	METH responses for home cage activity
	GS84278	(Published QTL)	chronic alcohol withdrawal severity
	GS84278	(Published QTL)	chronic alcohol withdrawal severity
	GS84279	(Published QTL)	METH responses for climbing
C1qa			
	GS86977	[DRG]	Table S1: All transcripts significantly different in abundance between
	GS87058	[DRG]	Table S2: Cocaine Regulation of H4 Acetylation. (provisional)
	GS243385	[MeSH]	Dose-Response Relationship, Drug : D004305
	GS235349	[MeSH]	Physiological Effects of Drugs : D045505
	GS1243	Differential Expression	KCgamma wild-type expression changes due to chronic ethanol diet
	GS83985	(Published QTL)	cocaine related behavior 16
	GS14933	Differential Expression	Upregulated gene expression of PKC-gamma wild type mice due to
	GS84164	(Published QTL)	cocaine related behavior
	GS83998	(Published QTL)	cocaine and amphetamine-regulated transcript
Cp			
	GS87096	[DRG]	Table S2: List of Cocaine-Treated HDAC5 KO vs. Cocaine-Treated WT
	GS87041	[DRG]	Table S3: List of Cocaine-Treated HDAC5 KO vs. Saline-Treated
	GS243385	[MeSH]	Dose-Response Relationship, Drug : D004305
	GS235349	[MeSH]	Physiological Effects of Drugs : D045505
Btg2			
	GS86789	[DRG]	Table S1: Cocaine Regulation of H3 Acetylation. (provisional)
	GS243385	[MeSH]	Dose-Response Relationship, Drug : D004305
	GS235349	[MeSH]	Physiological Effects of Drugs : D045505
	GS1243	Differential Expression	KCgamma wild-type expression changes due to chronic ethanol diet
	GS14933	Differential Expression	Upregulated gene expression of PKC-gamma wild type mice due to
	GS37147	Differential Expression	Gene expression change in the nucleus accumbens, following
Cfb			
	GS87128	[DRG]	Table S1: Genes with significant alterations in expression following
	GS243385	[MeSH]	Dose-Response Relationship, Drug : D004305
	GS235349	[MeSH]	Physiological Effects of Drugs : D045505
	GS127417	Differential Expression	Chronic alcohol exposure induced gene expression changes in the
	GS84303	(Published QTL)	differences in cocaine responsiveness
	GS84300	(Published QTL)	METH responses for body temperature
	GS84303	(Published QTL)	differences in cocaine responsiveness
	GS213106	Differential Expression	Chronic Alcohol HepG2
	GS84301	(Published QTL)	ethanol conditioned taste aversion
	GS83968	(Published QTL)	cocaine induced activation 13
	GS239299	[MeSH]	Drug Interactions : D004347
	GS135650	(Published QTL)	cocaine induced activation 13
	GS84302	(Published QTL)	differences in cocaine responsiveness
Dnajb3			
	GS87011	[DRG]	Table S2: List of Cocaine-Treated HDAC5 KO vs. Cocaine-Treated WT
	GS75590	Differential Expression	Cocaine Regulation of Dimethyl-K9/K27 H3
	GS83978	(Published QTL)	cocaine related behavior 1
	GS83973	(Published QTL)	cocaine induced activation 5
	GS84103	(Published QTL)	chronic alcohol withdrawal severity Chr1 at D1Mit46
	GS135653	(Published QTL)	cocaine induced activation 5
	GS135293	(Published QTL)	alcohol withdrawal 5
Hspb2			
	GS87011	[DRG]	Table S2: List of Cocaine-Treated HDAC5 KO vs. Cocaine-Treated WT
	GS31782	Differential Expression	Gene Expression Correlations with Hippocampus Consortium M430v2
	GS235349	[MeSH]	Physiological Effects of Drugs : D045505

	GS84180	(Published QTL)	METH responses for body temperature
	GS84181	(Published QTL)	ethanol induced locomotion
	GS84182	(Published QTL)	METH responses for home cage activity
	GS84179	(Published QTL)	cocaine related behavior
	GS135789	(Published QTL)	ethanol induced locomotor activity 2
	GS243385	[MeSH]	Dose-Response Relationship, Drug : D004305
	GS83991	(Published QTL)	cocaine related behavior 7
	GS235349	[MeSH]	Physiological Effects of Drugs : D045505
	GS128167	Differential Expression	Table S1: Genes differentially expressed in Lewis vs. Fisher nucleus
	GS246373	Differential Expression	Differential Expression Hippocampus Human Alcoholic
	GS84300	(Published QTL)	METH responses for body temperature
	GS84301	(Published QTL)	ethanol conditioned taste aversion
	GS83968	(Published QTL)	cocaine induced activation 13
	GS83971	(Published QTL)	cocaine induced activation 3
	GS84298	(Published QTL)	cocaine induced activation
	GS135650	(Published QTL)	cocaine induced activation 13
	GS84302	(Published QTL)	differences in cocaine responsiveness
	GS36452	Differential Expression	Whole Brain Gene expression correlates of Morphine - Postural Effects
	GS36477	Differential Expression	Whole Brain Gene expression correlates of Morphine - Severity of
	GS36457	Differential Expression	Whole Brain Gene expression correlates of Morphine - Postural Effects
	GS243385	[MeSH]	Dose-Response Relationship, Drug : D004305
	GS235349	[MeSH]	Physiological Effects of Drugs : D045505
	GS242550	[MeSH]	Alcoholism : D000437
	GS324475	GO	0008144 drug binding
	GS332669	GO	0017144 drug metabolic process
	GS269429	GWAS	Catalog Data for alcohol and nicotine codependence in 818 European
	GS246373	Differential Expression	Differential Expression Hippocampus Human Alcoholic
	GS236764	[MeSH]	Drug-Related Side Effects and Adverse Reactions : D064420
	GS239299	[MeSH]	Drug Interactions : D004347
	GS242397	[MeSH]	Psychotropic Drugs : D011619
	GS135737	(Published QTL)	dopamine receptor binding 2
	GS136242	(Published QTL)	methamphetamine response QTL 1
	GS84174	(Published QTL)	METH responses for chewing
	GS135490	(Published QTL)	behavioral response to methamphetamines 3
	GS135655	(Published QTL)	cocaine induced activation 7
	GS83975	(Published QTL)	cocaine induced activation 7
	GS84173	(Published QTL)	differences in cocaine responsiveness
	GS84172	(Published QTL)	cocaine related behavior
	GS246375	Differential Expression	H3K4me3 CHiP Seq Hippocampus Human Alcoholics
	GS84175	(Published QTL)	METH responses for climbing
	GS84176	(Published QTL)	METH responses for climbing
	GS84189	(Published QTL)	ethanol conditioned taste aversion
	GS127346	Differential Expression	Transcripts differentially regulated in hippocampus of C57BL/6J mice
	GS84190	(Published QTL)	METH responses for body temperature
	GS235349	[MeSH]	Physiological Effects of Drugs : D045505
	GS136244	(Published QTL)	methamphetamine response QTL 3
	GS327758	GO	0035690 cellular response to drug
	GS318209	GO	0017144 drug metabolic process
	GS326077	GO	0042493 response to drug
	GS135823	(Published QTL)	ethanol conditioned taste aversion 9
	GS37188	(Published QTL)	Positional candidate on Chromosome 11 (30-110 Mb) for dominant
	GS87096	[DRG]	Table S2: List of Cocaine-Treated HDAC5 KO vs. Cocaine-Treated WT

Cp	GS87041	[DRG]	Table S3: List of Cocaine-Treated HDAC5 KO vs. Saline-Treated
	GS243385	[MeSH]	Dose-Response Relationship, Drug : D004305
	GS235349	[MeSH]	Physiological Effects of Drugs : D045505
	GS84144	(Published QTL)	METH responses for home cage activity (Published QTL, Chr 3)
	GS84146	(Published QTL)	METH responses for home cage activity (Published QTL, Chr 3)
	GS128161	Differential Expression	Nucleus accumbens Methamphetamine and reward
	GS35864	Differential Expression	Neocortex Gene expression correlates of Cocaine CPP - difference in
	GS243385:	[MeSH]	Dose-Response Relationship, Drug : D004305
	GS86932	[DRG]	Table S3: CORTEX 17K MICROARRAY
	GS86494	[DRG]	Table S3: CORTEX 17K MICROARRAY
	GS243385	[MeSH]	Dose-Response Relationship, Drug : D004305
	GS128199		Alcohol Preference union of 86 Gene Sets
	GS135133	Differential Expression	bHR vs bLR genes different in Hippocampus
	GS135132	Differential Expression	bHR vs bLR genes different in Nucleus Acumbens
Dnaja4	GS14888	Differential Expression	Differentially expressed genes modulated by nicotine in five combined
	GS135660	(Published QTL)	cocaine related behavior 8 (Cocrb8, Published QTL Chr 9)
	GS135821	(Published QTL)	ethanol consumption 3 (Etohc3, Published QTL Chr 9)
	GS135647	(Published QTL)	cocaine induced activation 10 (Cocia10, Published QTL Chr 9)
	GS84219	(Published QTL)	cocaine related behavior (Published QTL, Chr 9)
	GS84218	(Published QTL)	differences in cocaine responsiveness (Published QTL, Chr 9)
	GS84217	(Published QTL)	differences in cocaine responsiveness (Published QTL, Chr 9)
	GS84208	(Published QTL)	METH responses for home cage activity (Published QTL, Chr 9)
	GS14914	Differential Expression	Differentially expressed genes in morphine-treated vs. saline-treated
	GS128199		Alcohol Preference union of 86 Gene Sets
	GS135133	Differential Expression	bHR vs bLR genes different in Hippocampus
	GS135132	Differential Expression	bHR vs bLR genes different in Nucleus Acumbens
Dnmt3a	GS243385:	[MeSH]	Dose-Response Relationship, Drug : D004305
	GS86932	[DRG]	Table S3: CORTEX 17K MICROARRAY
	GS86494	[DRG]	Table S3: CORTEX 17K MICROARRAY
	GS243385	[MeSH]	Dose-Response Relationship, Drug : D004305
	GS34054	Differential Expression	Hippocampus Gene expression correlates of Open Field locomotion
	GS34322	Differential Expression	Hippocampus Gene expression correlates of Cocaine TOTAL
	GS34005	Differential Expression	Hippocampus Gene expression correlates of Open Field locomotion 15-
	GS34332	Differential Expression	Hippocampus Gene expression correlates of Cocaine TOTAL
	GS246394	Differential Expression	Human hippocampus chronically exposed to cocaine
	GS84261	(Published QTL)	ethanol withdrawal (Published QTL, Chr 12)
	GS86746	[DRG]	Table S5: List of Cocaine-Treated WT vs. Saline-Treated WT
	GS246374	Differential Expression	Differential Expression Hippocampus Human Cocaine Addicts
	GS128199		Alcohol Preference union of 86 Gene Sets
Htr7	GS243385:	[MeSH]	Dose-Response Relationship, Drug : D004305
	GS243385	[MeSH]	Dose-Response Relationship, Drug : D004305
	GS242397	[MeSH]	Psychotropic Drugs : D011619
	GS14914	Differential Expression	Differentially expressed genes in morphine-treated vs. saline-treated
	GS242550	[MeSH]	Alcoholism : D000437
	GS84314	(Published QTL)	METH responses for body temperature (Published QTL, Chr 19)
	GS236200	[MeSH]	Neurotransmitter Uptake Inhibitors : D014179
	GS246374	Differential Expression	Differential Expression Hippocampus Human Cocaine Addicts
	GS128199		Alcohol Preference union of 86 Gene Sets
	GS135133	Differential Expression	bHR vs bLR genes different in Hippocampus
	GS135132	Differential Expression	bHR vs bLR genes different in Nucleus Acumbens
Irf8	GS243385:	[MeSH]	Dose-Response Relationship, Drug : D004305
	GS86932	[DRG]	Table S3: CORTEX 17K MICROARRAY
	GS86494	[DRG]	Table S3: CORTEX 17K MICROARRAY
	GS243385	[MeSH]	Dose-Response Relationship, Drug : D004305
	GS1139	Differential Expression	Differential expression response 4 hr after 2g/kg ethanol in C57BL/6J
	GS246374	Differential Expression	Differential Expression Hippocampus Human Cocaine Addicts

	GS128199		Alcohol Preference union of 86 Gene Sets
	GS135133	Differential Expression	bHR vs bLR genes different in Hippocampus
	GS135132	Differential Expression	bHR vs bLR genes different in Nucleus Acumbens
Lrrc15	GS84293	(Published QTL)	METH responses for home cage activity (Published QTL, Chr 16)
	GS35781	Differential Expression	Cerebellum Gene expression correlates of CPP - Time (s) in drug-
Myh10	GS37188	(Published QTL)	Positional candidate on Chromosome 11 (30-110 Mb) for dominant
	GS84251	(Published QTL)	chronic alcohol withdrawal severity (Published QTL, Chr 11)
	GS243385:	[MeSH]	Dose-Response Relationship, Drug : D004305
	GS135823	(Published QTL)	ethanol conditioned taste aversion 9 (Etohcta9, Published QTL Chr 11)
	GS37187	(Published QTL)	Positional candidate on chromosome 11 (59-79Mb) for overdominant
	GS86932	[DRG]	Table S3: CORTEX 17K MICROARRAY
	GS86494	[DRG]	Table S3: CORTEX 17K MICROARRAY
	GS243385	[MeSH]	Dose-Response Relationship, Drug : D004305
	GS127342	Differential Expression	Transcripts differentially regulated in frontal cortex of C57BL/6J mice
	GS75588	Differential Expression	Cocaine Regulation of H3 Acetylation
	GS137407	Differential Expression	Supplementary Table 2. Overall results of WGCNA combined with
	GS313343	Gene Ontology	GO:0008144 drug binding
	GS36154	Differential Expression	Neocortex Gene expression correlates of Locomotor response of 10
	GS75588	Differential Expression	Cocaine Regulation of H3 Acetylation
	GS128199		Alcohol Preference union of 86 Gene Sets
	GS135133	Differential Expression	bHR vs bLR genes different in Hippocampus
	GS135132	Differential Expression	bHR vs bLR genes different in Nucleus Acumbens
Rap2b	GS84146	(Published QTL)	METH responses for home cage activity (Published QTL, Chr 3)
	GS243385:	[MeSH]	Dose-Response Relationship, Drug : D004305
	GS84147	(Published QTL)	ethanol conditioned taste aversion (Published QTL, Chr 3)
	GS86932	[DRG]	Table S3: CORTEX 17K MICROARRAY
	GS86494	[DRG]	Table S3: CORTEX 17K MICROARRAY
	GS243385	[MeSH]	Dose-Response Relationship, Drug : D004305
	GS246373	Differential Expression	Differential Expression Hippocampus Human Alcoholic
	GS246394	Differential Expression	Human hippocampus chronically exposed to cocaine
	GS128199		Alcohol Preference union of 86 Gene Sets
Tmod2	GS35864	Differential Expression	Neocortex Gene expression correlates of Cocaine CPP - difference in
	GS14917	Differential Expression	Upregulation of gene expression in the lateral hypothalamus of Wild
	GS135660	(Published QTL)	cocaine related behavior 8 (Cocrb8, Published QTL Chr 9)
	GS135821	(Published QTL)	ethanol consumption 3 (Etohc3, Published QTL Chr 9)
	GS14916	Differential Expression	Mu opioid receptor-dependent genes regulated by chronic morphine in
	GS86932	[DRG]	Table S3: CORTEX 17K MICROARRAY
	GS86494	[DRG]	Table S3: CORTEX 17K MICROARRAY
	GS75567	Differential Expression	Genes that were significantly different in the nucleus accumbens of iP
	GS14929	Differential Expression	Ethanol-dependence genes in the nucleus accumbens (NA) of inbred
	GS75589	Differential Expression	Cocaine Regulation of H4 Acetylation
	GS137562	Differential Expression	Genes significantly differentially expressed in P7 selected High-
	GS128223	Differential Expression	Proteins found to be modified by at least two drugs of abuse
	GS135822	(Published QTL)	ethanol conditioned taste aversion 8 (Etohcta8, Published QTL Chr 9)
	GS84219	(Published QTL)	cocaine related behavior (Published QTL, Chr 9)
	GS84218	(Published QTL)	differences in cocaine responsiveness (Published QTL, Chr 9)
	GS84217	(Published QTL)	differences in cocaine responsiveness (Published QTL, Chr 9)
	GS246374	Differential Expression	Differential Expression Hippocampus Human Cocaine Addicts
	GS128199		Alcohol Preference union of 86 Gene Sets
	GS135133	Differential Expression	bHR vs bLR genes different in Hippocampus
	GS135132	Differential Expression	bHR vs bLR genes different in Nucleus Acumbens
Zbtb4	GS37188	(Published QTL)	Positional candidate on Chromosome 11 (30-110 Mb) for dominant
	GS84251	(Published QTL)	chronic alcohol withdrawal severity (Published QTL, Chr 11)
	GS135823	(Published QTL)	ethanol conditioned taste aversion 9 (Etohcta9, Published QTL Chr 11)
	GS37187	(Published QTL)	Positional candidate on chromosome 11 (59-79Mb) for overdominant

	GS137413	Differential Expression	Supplementary Table 2. CNA Overall results of WGCNA combined with
	GS246376	Differential Expression	H3K4me3 CHiP Seq Hippocampus Human Cocaine Addicts
	GS246376	Differential Expression	H3K4me3 CHiP Seq Hippocampus Human Cocaine Addicts
	GS246373	Differential Expression	Differential Expression Hippocampus Human Alcoholic

Supplemental Table 18

Multiple Members Biological Process		
**GO ID**	**GO Term Definition**	**Genes**
GO:0000122	negative regulation of transcription from RNA polymerase II promoter	*Btg2*<br>*Dnase1l2*<br>*Hdac10*
GO:0006351	transcription, DNA-templated	*Btg2*<br>*Dnase1l2*<br>*Hdac10*<br>*Pitx3*
GO:0006355	regulation of transcription, DNA-templated	*Btg2*<br>*Dnase1l2*<br>*Hdac10*<br>*Pitx3*
GO:0006508	proteolysis	*Cfb*<br>*C9*
GO:0006629	lipid metabolic process	*Far2*<br>*Pnmt*<br>*C9*
GO:0006810	transport	*Cp*<br>*Rilpl2*
GO:0006957	complement activation, alternative pathway	*Cfb*<br>*C9*
GO:0006958	complement activation, classical pathway	*C1qa*<br>*C9*
GO:0007186	G-protein coupled receptor signaling pathway	*Htr1a*<br>*Lpar6*
GO:0008283	cell proliferation	*Cfb*<br>*Htr1a*
GO:0008285	negative regulation of cell proliferation	*Btg2*<br>*Pnmt*
GO:0010468	regulation of gene expression	*Pitx3*<br>*Dnase1l2*

SUPPLEMENTAL REFERNECES

1. Kuniishi H, Ichisaka S, Yamamoto M, et al. Early deprivation increases high-leaning behavior, a novel anxiety-like behavior, in the open field test in rats. In. *Neuroscience Research.* Vol 123: Elsevier Ireland Ltd and Japan Neuroscience Society; 2017:27-35.
2. Crawley JN, Belknap JK, Collins A, et al. Behavioral phenotypes of inbred mouse strains: implications and recommendations for molecular studies. *Psychopharmacology (Berl).* 1997;132(2):107-124.
3. Dickson PE, Ndukum J, Wilcox T, et al. cocaine self-administration in Diversity Outbred mice. In. Vol 2322016:1011-1024.
4. Salomons AR, Bronkers G, Kirchhoff S, Arndt SS, Ohl F. Behavioural habituation to novelty and brain area specific immediate early gene expression in female mice of two inbred strains. *Behav Brain Res.* 2010;215(1):95-101.
5. Prut L, Belzung C. The open field as a paradigm to measure the effects of drugs on anxiety-like behaviors: a review. *Eur J Pharmacol.* 2003;463(1-3):3-33.
6. Wingo T, Nesil T, Choi JS, Li MD. Novelty Seeking and Drug Addiction in Humans and Animals: From Behavior to Molecules. *J Neuroimmune Pharmacol.* 2016;11(3):456-470.
7. Bourin M, Hascoet M. The mouse light/dark box test. *Eur J Pharmacol.* 2003;463(1-3):55-65.
8. Abdi H, Williams LJ. Principal component analysis. *WIREs Computational Statistics.* 2010;2(4):433-459.
9. Trullas R, Jackson B, Skolnick P. Genetic differences in a tail suspension test for evaluating antidepressant activity. In. *Psychopharmacology.* Vol 991989:287-288.
10. Ng E, Browne CJ, Samsom JN, Wong AHC. Depression and substance use comorbidity: What we have learned from animal studies. In. *American Journal of Drug and Alcohol Abuse.* Vol 43: Taylor & Francis; 2017:456-474.
11. Gu Z, Chu L, Han Y. Therapeutic effect of resveratrol on mice with depression. *Exp Ther Med.* 2019;17(4):3061-3064.
12. Song J, Ma W, Gu X, et al. Metabolomic signatures and microbial community profiling of depressive rat model induced by adrenocorticotrophic hormone. *J Transl Med.* 2019;17(1):224.
